# Catalyst and Medium Control over Rebound Pathways in Manganese-Catalyzed Methylenic C–H Bond Oxidation

**DOI:** 10.1021/jacs.3c11555

**Published:** 2024-03-20

**Authors:** Marco Galeotti, Massimo Bietti, Miquel Costas

**Affiliations:** † QBIS Research Group, Institut de Química Computacional i Catàlisi (IQCC) and Departament de Química, Universitat de Girona, Campus Montilivi, Girona E-17071, Catalonia, Spain; ‡ Dipartimento di Scienze e Tecnologie Chimiche, Università “Tor Vergata”, Via della Ricerca Scientifica, 1, I-00133 Rome, Italy

## Abstract

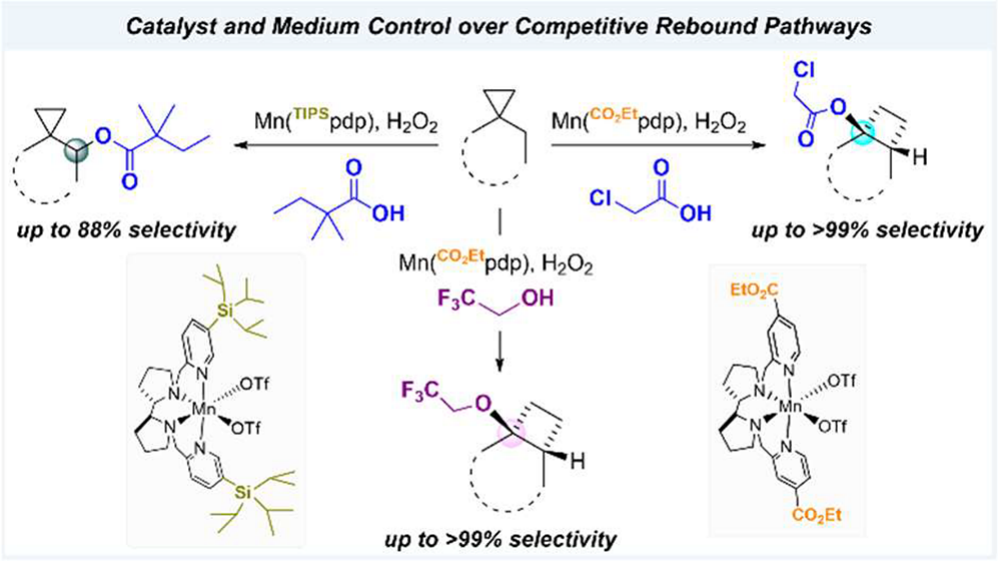

The C(sp^3^)–H bond oxygenation of a variety of cyclopropane containing hydrocarbons with hydrogen peroxide catalyzed by manganese complexes containing aminopyridine tetradentate ligands was carried out. Oxidations were performed in 1,1,1,3,3,3-hexafluoro-2-propanol (HFIP) and 2,2,2-trifluoroethanol (TFE) using different manganese catalysts and carboxylic acid co-ligands, where steric and electronic properties were systematically modified. Functionalization selectively occurs at the most activated C–H bonds that are α- to cyclopropane, providing access to carboxylate or 2,2,2-trifluoroethanolate transfer products, with no competition, in favorable cases, from the generally dominant hydroxylation reaction. The formation of mixtures of unrearranged and rearranged esters (oxidation in HFIP in the presence of a carboxylic acid) and ethers (oxidation in TFE) with full control over diastereoselectivity was observed, confirming the involvement of delocalized cationic intermediates in these transformations. Despite such a complex mechanistic scenario, by fine-tuning of catalyst and carboxylic acid sterics and electronics and leveraging on the relative contribution of cationic pathways to the reaction mechanism, control over product chemoselectivity could be systematically achieved. Taken together, the results reported herein provide powerful catalytic tools to rationally manipulate ligand transfer pathways in C–H oxidations of cyclopropane containing hydrocarbons, delivering novel products in good yields and, in some cases, outstanding selectivities, expanding the available toolbox for the development of synthetically useful C–H functionalization procedures.

## Introduction

**Scheme 1:**
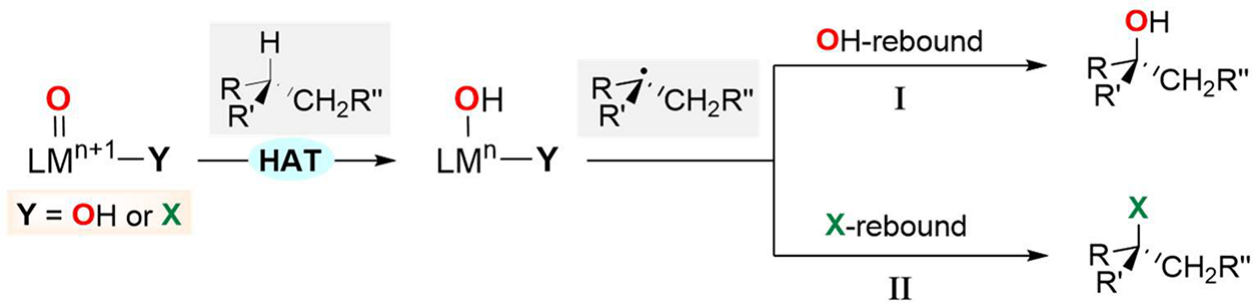
Competitive Rebound Pathways in C–H Bond Oxidation Promoted by Enzymes and Bioinspired Catalysts (X = Halogen or Pseudohalogen)

The ubiquity of oxidized aliphatic frameworks in molecules of biological and pharmaceutical interest makes the conversion of C(*sp*^3^)–H into C(*sp*^3^)–O bonds a preferential transformation in modern synthetic organic chemistry.^1,2^ Among the numerous complexes able to perform catalytic C–H bond oxidations, homogeneous catalysts based on first-row transition metals, that in the presence of hydrogen peroxide mimic the mode of action of oxygenases, represent an efficient way to perform these transformations.^1a,2,3^ C–H bond oxidations executed by enzymes and bioinspired catalysts proceed through well-established radical mechanisms, where a high-valent metal–oxo species engages in hydrogen atom transfer (HAT) from a substrate C–H bond to give a carbon radical that is then most commonly trapped by hydroxyl ligand transfer (OH rebound) to form the hydroxylated product (Scheme 1, path I).^4−6^

Monoiron-dependent non-heme enzymes can display also alternative rebound mechanisms, where halide and pseudohalide ligands are transferred instead of hydroxyl.^7−9^ For example, in the catalytic C–H oxidation promoted by non-heme iron, O_2_- and α-ketoglutarate-dependent halogenases, formation of halogenated products was observed, suggesting that the structural versatility of the non-heme metal coordination sphere enables alternative reactivity patterns (Scheme 1, path II).^8^

**Figure 1:**
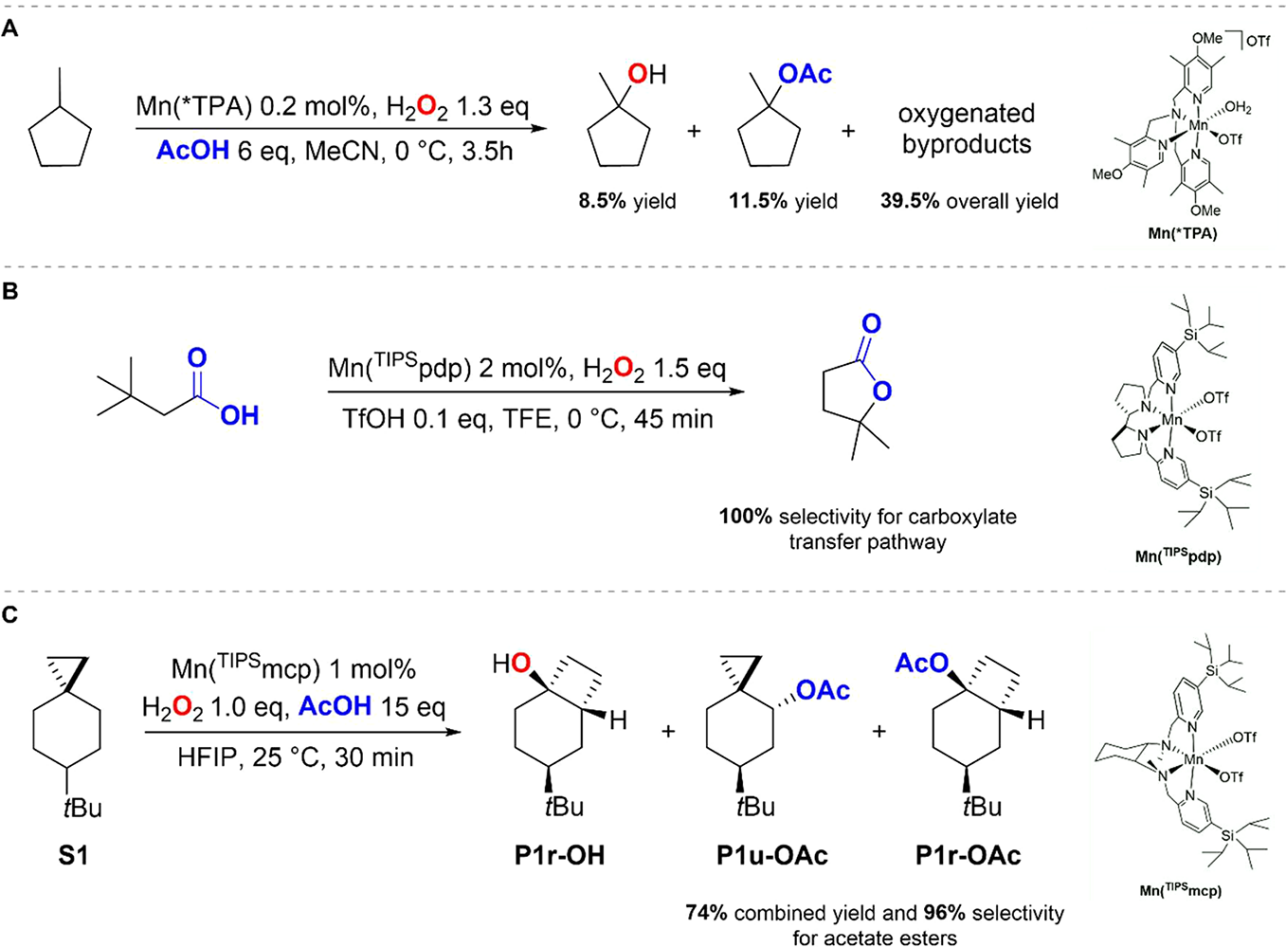
State of the art for manganese-catalyzed C(sp^3^)–H oxidation via carboxylate rebound: (A) ref (9), (B) ref (15)a, and (C) ref (16).

Iron and manganese complexes containing tetradentate aminopyridine ligands have been shown to promote catalytic C–H oxygenation via an enzymatic-like HAT/OH-rebound mechanism.^3b−3d,5,6,10^ C–H functionalization products derived from competitive ligand transfer pathways are seldom observed, and when they operate, the canonical hydroxylation reaction typically prevails (Figure 1).^11−13^ For example, Bryliakov and co-workers have recently reported the results of a mechanistic study on undirected C–H bond oxidation with H_2_O_2_ in the presence of bioinspired catalysts and carboxylic acid co-ligands, showing the occurrence of two competitive rebound pathways (−OH and −O_2_CR), which are responsible for the formation of alcohol and ester product mixtures.^9,14^ Tertiary C–H bond oxidation of a variety of hydrocarbons was performed with [Mn(OTf)(*TPA)(OH_2_)](OTf), Mn(*TPA), and AcOH. In the reaction of methylcyclopentane, the tertiary alcohol and acetate ester products were formed in 8.5% and 11.5% yield, respectively, and overall 34% selectivity over the other oxygenated products (Figure 1A).^9^ Isotopic labeling analyses have also shown the involvement of a carboxylate transfer step in the directed C(*sp*^3^)–H bond γ-lactonization of alkanoic acids catalyzed by [Mn(OTf)_2_(^TIPS^pdp)], Mn(^TIPS^pdp).^15^ When dealing in particular with primary C–H bonds, the carboxylate transfer pathway is largely dominant, an observation that was further substantiated by DFT studies which indicated that in these reactions carboxylate transfer is kinetically favored over hydroxyl transfer (Figure 1B).^15a^

Very recently, we have performed a detailed mechanistic study on the oxidation of 6-*tert*-butylspiro[2.5]octane (**S1**) with H_2_O_2_ catalyzed by [Mn(OTf)_2_(^TIPS^mcp)] (from now on indicated as Mn(^TIPS^mcp)) (Figure 1C).^16^ By using AcOH as co-ligand and 1,1,1,3,3,3-hexafluoro-2-propanol (HFIP) as the solvent, stereospecific formation of the unrearranged and rearranged acetate esters (**P1u-OAc** and **P1r-OAc**, respectively) in 74% combined yield and 96% selectivity over the rearranged alcohol product (**P1r-OH**) was observed, pointing toward carboxylate transfer as the main rebound pathway under these experimental conditions. Very interestingly, the formation of rearranged alcohol and ester products provides conclusive evidence that with this substrate stereospecific C(sp^3^)–H oxidation can take place via a delocalized cationic intermediate, accessible because of the characteristic structural and bonding features of the cyclopropyl moiety.^17−19^

**Figure 2:**
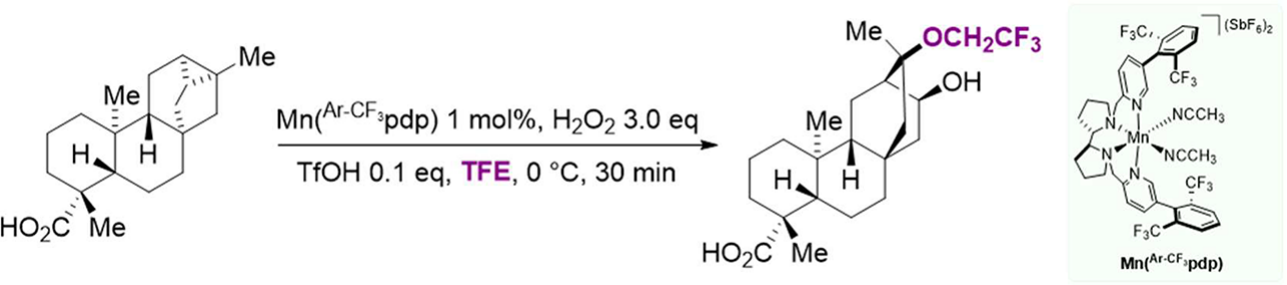
Oxidation of *ent*-trachyloban-19-oate with H_2_O_2_ catalyzed by Mn(^Ar–CF_3_^pdp).

Surprisingly, Magauer reported the oxidation of a polycyclic carboxylic acid substrate bearing a cyclopropyl group (*ent*-trachyloban-19-oate) with H_2_O_2_ catalyzed by [Mn(CH_3_CN)_2_(^Ar–CF_3_^pdp)](SbF_6_)_2_ (Mn(^Ar–CF_3_^pdp)), in 2,2,2-trifluoroethanol (TFE) as the solvent, where formation of a rearranged hydroxylated product containing a 2,2,2-trifluoroethoxy group was observed in 30% isolated yield (Figure 2).^20^

Although the reaction mechanism was not elucidated, the formation of this product was proposed to occur by TFE transfer, with the presence of the cyclopropyl group that plays again a major role in governing product selectivity.

With these concepts in mind, we sought to develop catalytic C(*sp*^3^)–H oxidation methodologies where alternative pathways can prevail over the hydroxylation reaction, providing access to differently functionalized products. For this purpose, the product selectivity was studied in the oxidation of **S1** and of a variety of cyclopropane containing hydrocarbons with H_2_O_2_ using different manganese catalysts and carboxylic acid co-ligands, where steric and electronic properties have been systematically modified. We find that by fine-tuning of catalyst structure and reaction conditions, the formation of carboxylate- and TFE-rebound products is accomplished in good yields and outstanding selectivities, overriding the generally dominant competitive hydroxylation reaction.

## Results and Discussion

### Reaction Development

**Scheme 2:**
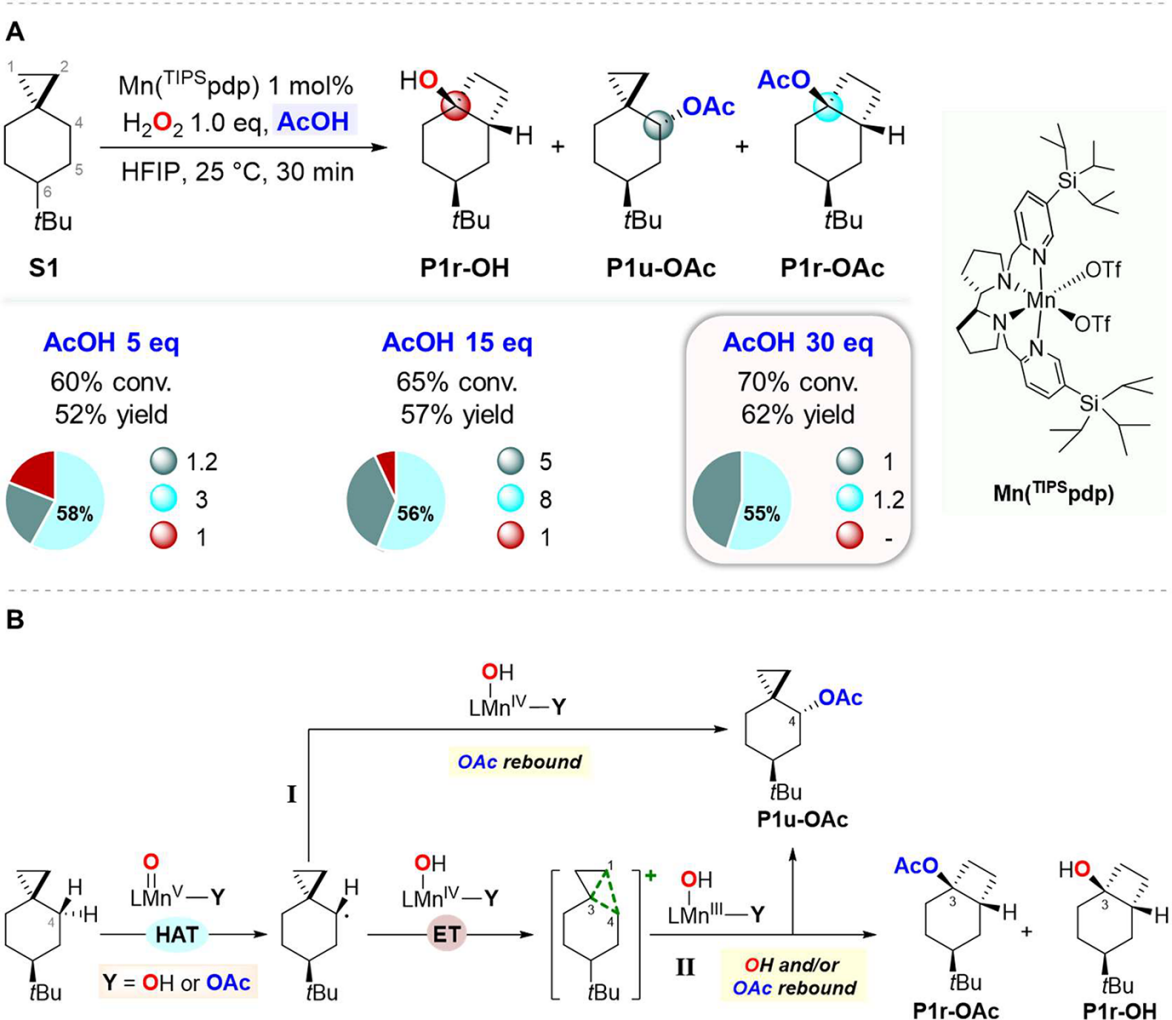
(A) Effect of Acetic Acid on the Oxidation of **S1**; (B) Proposed Mechanistic Pathways for Carboxylate and Hydroxyl Transfer in the Oxidation of **S1** Pie charts refer to product selectivities while adjacent small circles to normalized product ratios.

As reported in our previous work, the oxidation of **S1** performed using 1 mol % of Mn(^TIPS^mcp) and 1.5 equiv of H_2_O_2_ in the presence of 15 equiv of AcOH in HFIP at 25 °C provided the unrearranged (*trans*-6-*tert*-butylspiro[2.5]octan-4-yl acetate, **P1u-OAc**) and rearranged (*cis*-4-(*tert*-butyl)bicyclo[4.2.0]octan-1-yl acetate, **P1r-OAc**) esters in 74% combined yield (**P1u-OAc/P1r-OAc** = 1.2) and 96% selectivity over the rearranged alcohol (*cis*-4-(*tert*-butyl)bicyclo[4.2.0]octan-1-ol, **P1r–OH**) (see Figure 1C).^16^ Similar results were obtained when Mn(^TIPS^pdp) (**1**) was employed as the catalyst in place of Mn(^TIPS^mcp) (see Table S1). In the present work, **1** was chosen as the reference catalyst because of the availability of a wider library of pdp-based catalysts compared to the mcp ones. The oxidation of **S1** was then performed using 1 mol % of **1** and 1.0 equiv of H_2_O_2_ delivered over 30 min using a syringe pump in HFIP (0.125 M substrate concentration) at 25 °C, in the presence of different amounts of AcOH (Scheme 2A) (see Table S2).

With 5 equiv of AcOH, the formation of **P1r-OAc** and **P1u-OAc** in 30% and 12% yield, respectively, was observed, accompanied by **P1r-OH** in 10% yield, resulting in 81% selectivity for carboxylate over hydroxyl rebound products. As previously reported, no products deriving from C–H bond oxidation at C-1, C-2, C-5, and C-6 were observed.^16^ When the same reaction was performed in the presence of 15 and 30 equiv of AcOH, the esters were obtained in 93% and 100% combined selectivity, respectively, providing in the latter case only the two esters in 62% total yield (**P1r-OAc**/**P1u-OAc** = 1.2:1). The increase in selectivity for the esters observed with increasing AcOH loading (from 5 to 30 equiv) is in line with the proposed competition between carboxylate and hydroxyl transfer (Scheme 2B). With 30 equiv of AcOH, largely predominant formation of the Mn^V^-oxo carboxylato over the Mn^V^-oxo hydroxo species can be envisaged, resulting in the exclusive transfer of the carboxylate group promoted by a Mn^IV^-(OH)(carboxylato) species (for the formation of **P1u-OAc**) or Mn^III^-(OH)(carboxylato) species (for the formation of **P1u-OAc** and **P1r-OAc**) after the HAT (Scheme 2B, path I,) or the HAT/ET processes (Scheme 2B, path II), respectively. This result deserves special attention because it indicates that under these reaction conditions, the hydroxyl rebound pathway is completely suppressed.^21^ Support in favor of the formation of a short-lived delocalized cationic intermediate is also provided by the results obtained in the experiments performed in the presence of external nucleophiles (Cl^–^, Br^–^, HSO_4_^–^, H_2_PO_4_^–^, AcO^–^, CN^–^, and N_3_^–^), where no evidence for nucleophile incorporation was obtained (see Table S3 for full details).

### Catalyst Control over Carboxylate Transfer

**Scheme 3:**
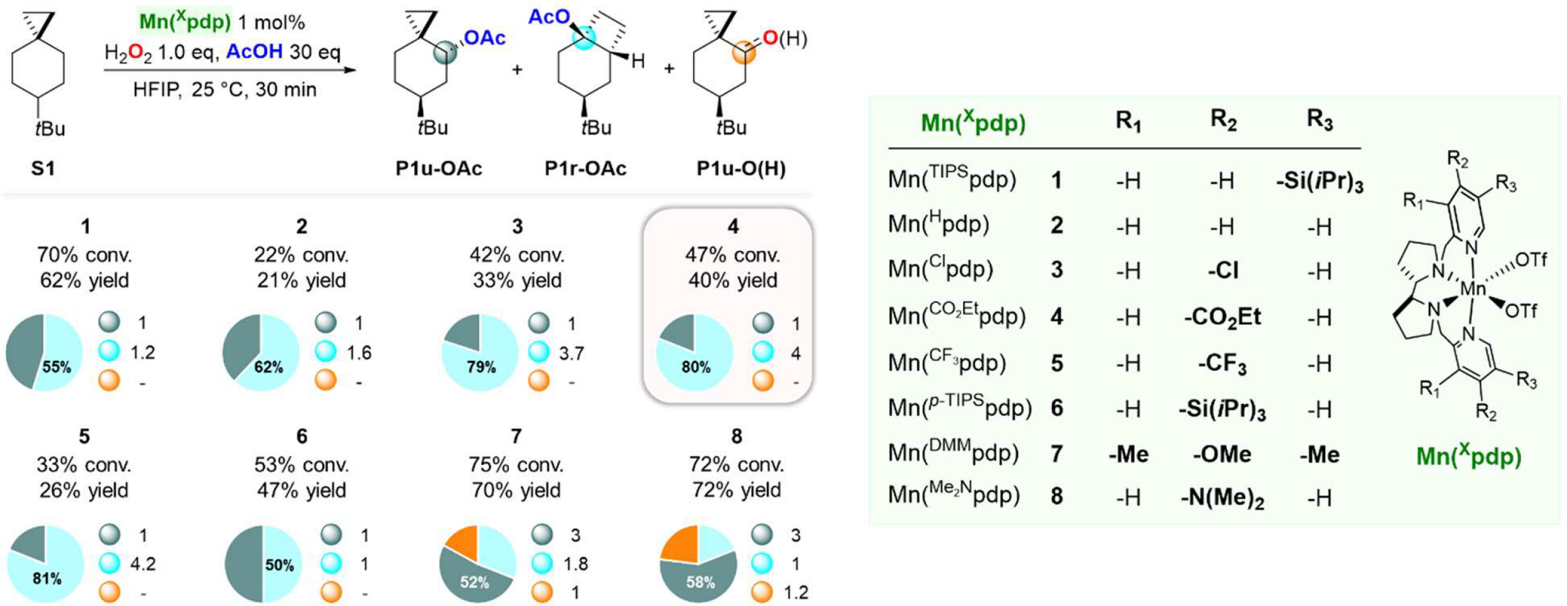
Effect of Catalyst Structure on the Oxidation of **S1** Pie charts refer to product selectivities while adjacent small circles to normalized product ratios.

With these results in hand, the role of the electronic and steric properties of the manganese catalysts was investigated. For this purpose, the oxidation of **S1** was performed using 1.0 equiv of H_2_O_2_ and 30 equiv of AcOH in HFIP at 25 °C in the presence of 1 mol % of a series of catalysts (Scheme 3). In order to obtain mechanistic information about the electronics, the catalysts were mainly modified by substitution of a C-4 hydrogen of the pyridine group of the Mn(pdp) complex (**2**) with electron withdrawing and releasing groups, namely Cl (**3**), CO_2_Et (**4**), CF_3_ (**5**), OMe (**7**), and Me_2_N (**8**). Mn(^*p*-TIPS^pdp) (**6**) was also considered for testing catalyst sterics by comparison with **1** (see Table S4).

In the oxidation of **S1** with catalysts **1**–**5**, **P1r-OAc** was in all cases the major product, accompanied by varying amounts of **P1u-OAc**. Similar **P1r-OAc/P1u-OAc** ratios were obtained when **1** and **2** were used (**P1r-OAc/P1u-OAc** = 1.2 and 1.6, respectively), associated however with a significant decrease in yield when employing the latter catalyst (62% and 21% combined yield for oxidations catalyzed by **1** and **2**, respectively). Very interestingly, when electron-poor catalysts **3**, **4**, and **5** were used, **P1r-OAc** was obtained in significantly higher selectivity (79–81%) over **P1u-OAc** (**P1r-OAc**/**P1u-OAc** = 3.7, 4.0, and 4.2, respectively). With **6**, **P1r-OAc** and **P1u-OAc** were obtained in 47% combined yield and a 1:1 ratio, whereas a significant change in selectivity was observed when the same reaction was performed with catalysts containing electron releasing groups such as OMe (**7**) and NMe_2_ (**8**). In particular with the latter, **P1r-OAc** was observed as a minor product (13% yield), accompanied by **P1u-OAc** in 42% yield and by sizable amounts of products arising from hydroxyl transfer (**P1u-O(H)**, 16% combined yield). From a mechanistic perspective these results are noteworthy. The presence of an EWG at C-4 of the pyridine group (as in catalysts **3**–**5**) increases the electrophilicity and oxidizing power of the catalyst, strongly favoring the formation of rearranged ester **P1r-OAc**. The observed selectivity can be explained in terms of an increase in the relative contribution of the cationic pathway to the overall mechanism (path II, Scheme 2B), where carboxylate transfer to the tertiary site of the delocalized cation (C-3) is favored over that to the secondary one (C-4). On the other hand, no significant influence on product distribution was observed when a bulky group (TIPS) was installed at C-4 of the pyridine (**6**), indicating that catalyst sterics play a minor role in governing chemoselectivity. The oxidation of **S1** promoted by **7** and **8** supports the proposed model, where the weaker oxidizing ability of these electron rich catalysts decreases the relative importance of the cationic pathway, increasing at the same time that of the hydroxyl rebound process. Overall, the results reported herein point toward fine control over product selectivity in C–H bond oxidations driven by manganese catalyst electronics.

**Scheme 4:**
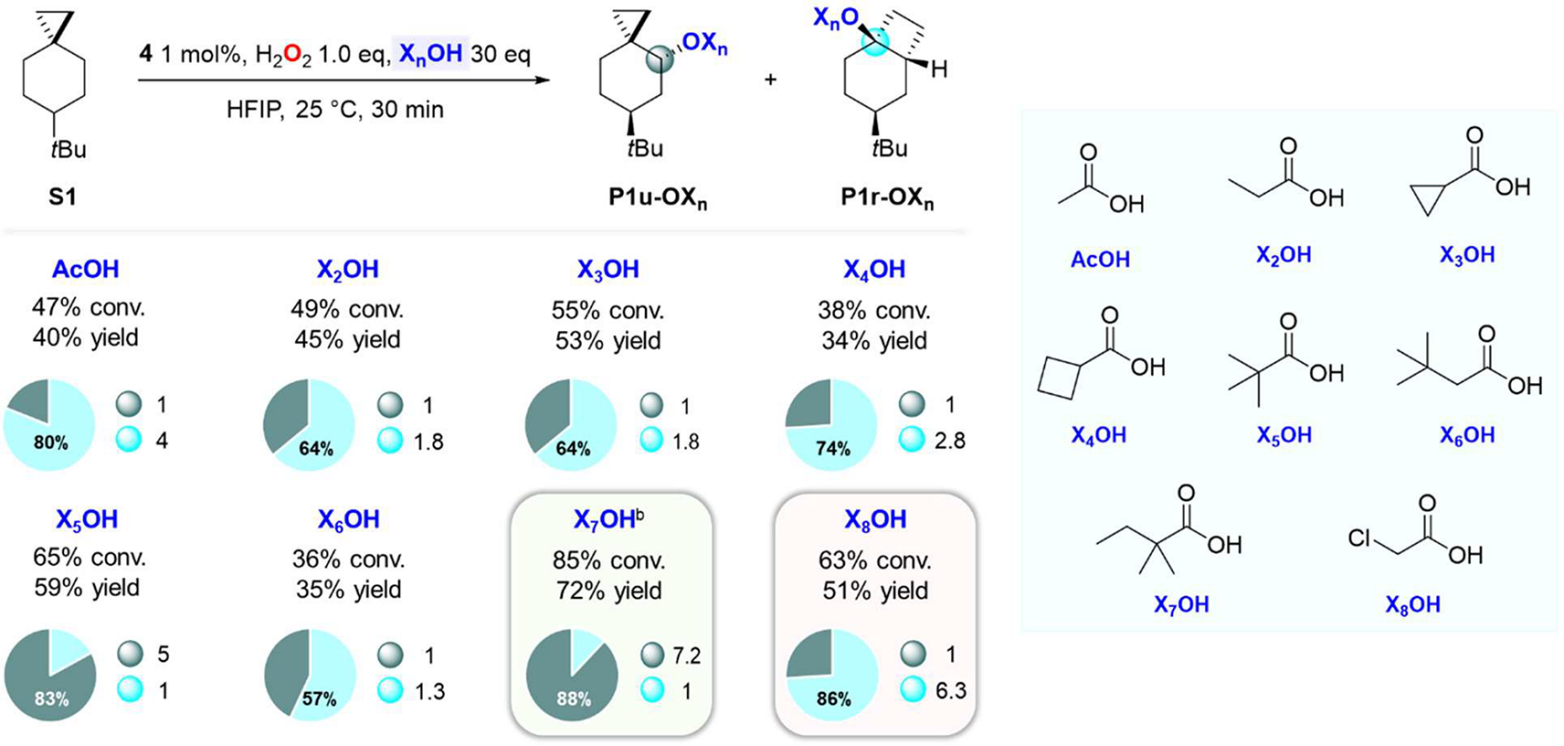
Effect of the Carboxylic Acid (X_*n*_OH) on the Oxidation of **S1**, Pie charts refer to product selectivities while adjacent small circles to normalized product ratios. Mn(^TIPS^pdp) 1 mol % and H_2_O_2_ 3.0 equiv were used.

At this point, we envisioned the possibility that the carboxylic acid co-ligand could have a major role in governing ester product selectivity, being the Mn(OH)(carboxylato) species responsible for carboxylate transfer. Taking as reference the oxidation of **S1** catalyzed by the electron-poor Mn(^CO_2_Et^pdp) catalyst (**4**), the effect of the carboxylic acid structure was then studied. The oxidation of **S1** was performed with 1 mol % of **4** and 1.0 equiv of H_2_O_2_ in HFIP at 25 °C in the presence of 30 equiv of different carboxylic acids X_*n*_OH, leading in all cases to the exclusive formation of the unrearranged (**P1u-OX**_***n***_) and rearranged (**P1r-OX**_***n***_) ester products (Scheme 4) (see Table S5). The consistent lack of alcohols among the reaction products is notable, pointing toward catalyst control over the rebound pathway as a general feature of these reactions.

**Scheme 5:**
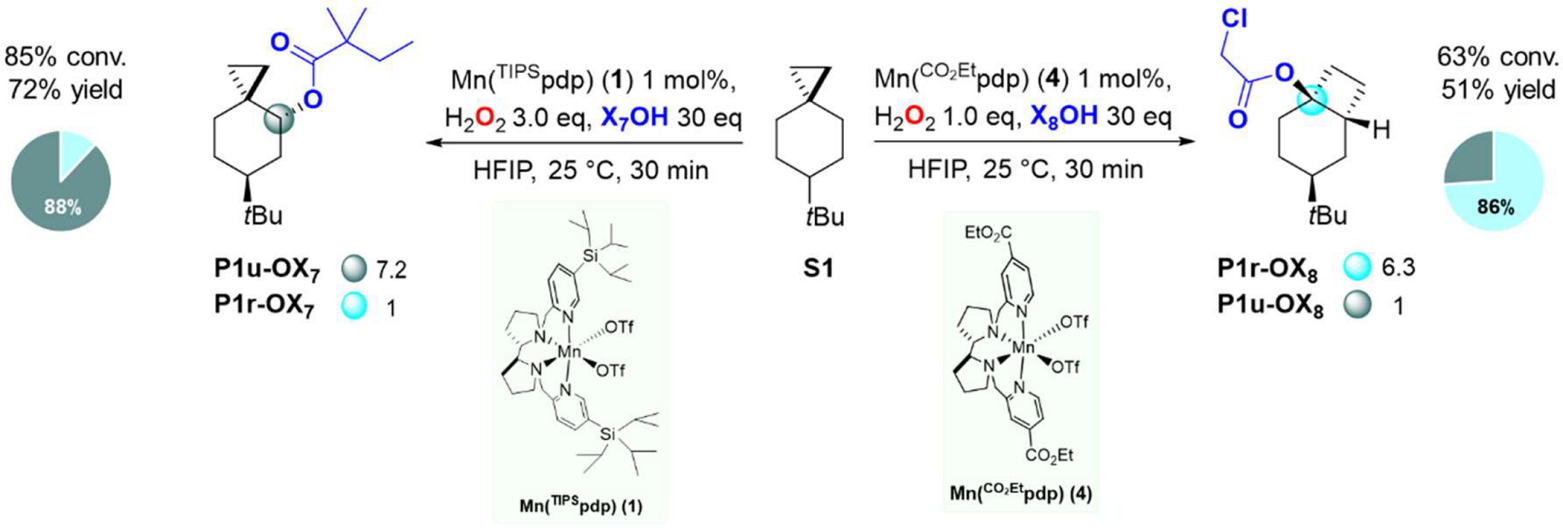
Steric and Electronic Effects on the Oxidation of **S1** Pie charts refer to product selectivities while adjacent small circles to normalized product ratios.

Compared to AcOH, the **P1r-OX**_***n***_/**P1u-OX**_***n***_ ratio was observed to decrease when propanoic (**X**_**2**_**OH**), cyclopropanecarboxylic (**X**_**3**_**OH**), and cyclobutanecarboxylic acid (**X**_**4**_**OH**) were used, providing **P1r-OX**_***n***_ in 64% (**P1r-OX**_**2**_/**P1u-OX**_**2**_ = 1.8), 64% (**P1r-OX**_**3**_/**P1u-OX**_**3**_ = 1.8), and 74% selectivity (**P1r-OX**_**4**_/**P1u-OX**_**4**_ = 2.8), respectively. These results point toward a modest effect on product distribution determined by substitution of one or two hydrogens on the α carbon of the acid with a methyl, cyclopropyl, or cyclobutyl group. On the other hand, when the oxidation of **S1** was performed in the presence of pivalic acid (**X**_**5**_**OH**), predominant formation of the unrearranged pivalate ester (**P1u-X**_**5**_) in 49% yield was observed accompanied by **P1r-OX**_**5**_ in 10% yield (**P1r-OX**_**5**_/**P1u-OX**_**5**_ = 0.2). The drastic decrease in selectivity for **P1r-OX**_***n***_ observed under these conditions (17%) compared to the value obtained with AcOH (80%) can be explained on steric grounds, where substitution of all hydrogens on the α carbon with methyl groups promotes carboxylate transfer toward the less sterically hindered site (C-4, Scheme 2) of the delocalized cationic intermediate. To support this hypothesis, when the oxidation reaction was performed in the presence of 3,3-dimethylbutanoic acid (**X**_**6**_**OH**), a 6.5-fold increase in the **P1r-OX**_**6**_/**P1u-OX**_**6**_ ratio (1.3) compared to that obtained with **X**_**5**_**OH** (0.2) was observed, suggesting that the presence of alkyl groups at the β carbon of the acid has no strong influence on the selectivity of the carboxylate rebound pathway. By combining this evidence and optimizing the reaction conditions, the oxidation of **S1** was performed with **1** in the presence of 2,2-dimethylbutanoic acid (**X**_**7**_**OH**) and 63% yield of the unrearranged ester **P1u-OX**_**7**_ in 88% selectivity over **P1r-OX**_**7**_ was obtained (**P1r-OX**_**7**_/**P1u-OX**_**7**_ = 0.14). Most interestingly, when an unhindered and electron poor acid such as chloroacetic acid (**X**_**8**_**OH**) was used in combination with catalyst **4**, a drastic change in product selectivity was observed with **P1r-OX**_**8**_/**P1u-OX**_**8**_ = 6.3 (86% selectivity for **P1r-OX**_**8**_) in a combined 51% yield. Taken together, these results show that the synergistic combination of catalyst electronics and carboxylic acid sterics and electronics determines an outstanding 45-fold increase in selectivity for **P1r-OX**_**8**_ compared to **P1r-OX**_**7**_, when the oxidation of **S1** was performed with **X**_**8**_**OH** (in combination with **4**) and **X**_**7**_**OH** (in combination with **1**), respectively (Scheme 5). These results clearly indicate that by leveraging the formation of a delocalized cationic intermediate, the selectivities of the carboxylate rebound products are strongly affected by the nature of the catalyst and can be rationally manipulated.

### Solvent Transfer

**Scheme 6:**
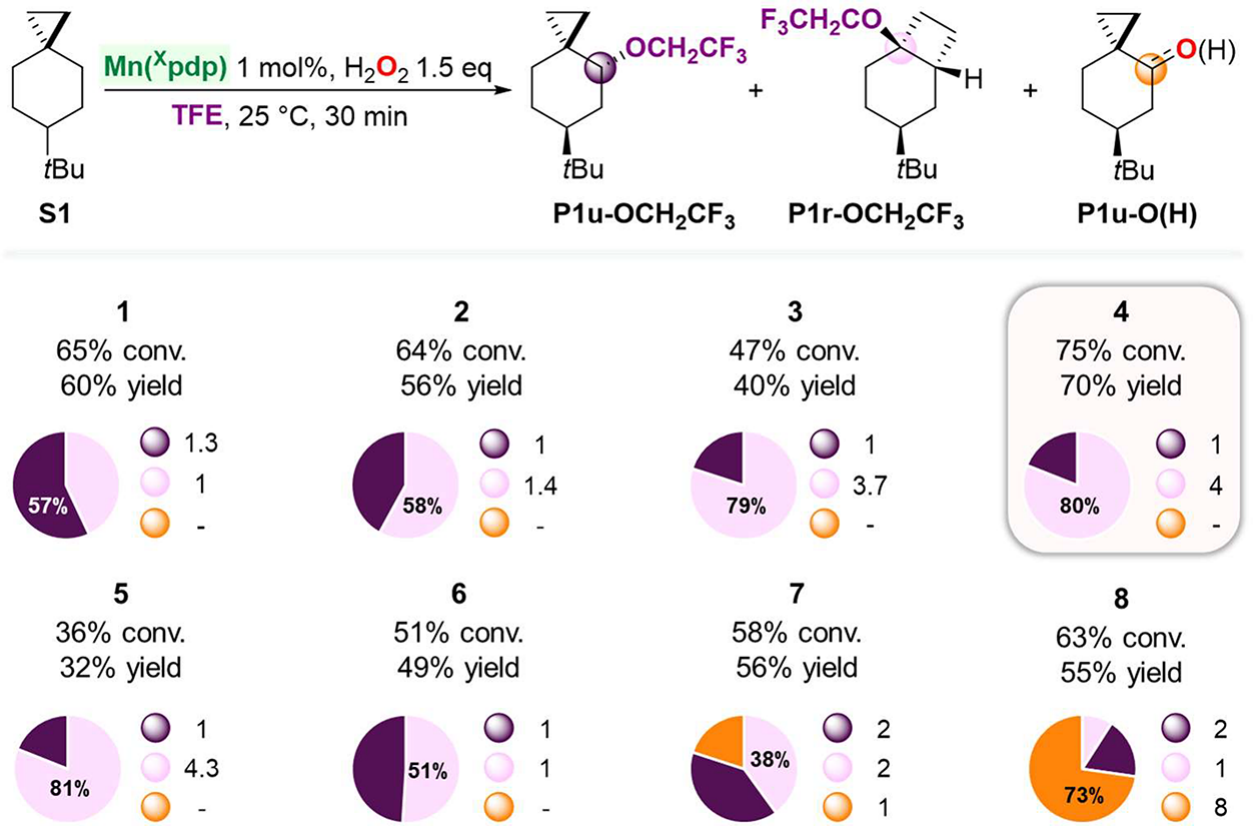
Oxidation of **S1** with H_2_O_2_ in 2,2,2-Trifluoroethanol (TFE) Catalyzed by Complexes **1**–**8**, the Structures for Which Are Displayed in Scheme 3 Pie charts refer to product selectivities while adjacent small circles to normalized product ratios.

As mentioned in the Introduction, previous work by Magauer and co-workers suggested the involvement of a TFE transfer in Mn-catalyzed C(*sp*^3^)–H oxidations (Figure 2).^20^ However, in all the experiments described above, C–H functionalization products deriving from HFIP or nonafluoro tert-butyl alcohol (NFTBA) transfer were never detected, with the role of these fluorinated alcohol solvents that appears to be mainly related to increase the reactivity of the manganese catalysts, presumably via hydrogen bonding^16^ and, when employing a carboxylic acid co-ligand, promote the exclusive formation of ester products. Along this line, in order to probe whether the proposed model for carboxylate transfer also holds for TFE, we have studied the oxidation of **S1** in this solvent. Gratifyingly, when this reaction was performed employing 1 mol % of **1** and 1.5 equiv of H_2_O_2_, in TFE at 25 °C and in the absence of carboxylic acid, exclusive formation of the rearranged **P1r-OCH**_**2**_**CF**_**3**_ and unrearranged **P1u-OCH**_**2**_**CF**_**3**_ ether products deriving from TFE transfer were observed in 26% and 34% yield, respectively (**P1r-OCH**_**2**_**CF**_**3**_/**P1u-OCH**_**2**_**CF**_**3**_ = 0.77) (Scheme 6). Reactions performed in 2-fluoroethanol and 2,2-difluoroethanol delivered reduced amounts of solvent transfer products (see Scheme S6 for full details). For comparison, in HFIP the same reaction led to the exclusive formation of hydroxylation and ketonization products (**P1u-OH**, **P1-O**, and **P1r-OH**), whereas HFIP-derived ethers were not detected among the reaction products.^16^ The singular transfer of TFE can be reasonably explained on the basis of the weaker nucleophilicity and greater steric demand of HFIP compared to TFE.

The oxidation of **S1** was then performed employing catalysts **2**–**8**. The product distributions deriving from the TFE rebound pathways depend again on catalyst electronics, showing trends that parallel those observed in carboxylic acid transfer. As previously reported in Scheme 3 for the formation of the acetate esters, compared to **1**, with the Mn(pdp) and Mn(^*p*-TIPS^pdp) catalysts (**2** and **6**, respectively) similar combined yields (56% and 49% respectively) and ratios (**P1r-OCH**_**2**_**CF**_**3**_/**P1u-OCH**_**2**_**CF**_**3**_ = 1.4 and 1.0, respectively) were observed (see Table S6). However, when the oxidation of **S1** was performed with the electron-poor catalysts (**3**–**5**) an increase in selectivity up to 79–81% for **P1r-OCH**_**2**_**CF**_**3**_ over **P1u-OCH**_**2**_**CF**_**3**_ was observed, confirming that also in this case the relative importance of the cationic pathway is increased by the use of more electrophilic and oxidizing catalysts. The best result was obtained when employing **4**, providing **P1r-OCH**_**2**_**CF**_**3**_ in 56% yield, accompanied by **P1u-OCH**_**2**_**CF**_**3**_ in 14% yield (**P1r-OCH**_**2**_**CF**_**3**_/**P1u-OCH**_**2**_**CF**_**3**_ = 4.0). When the oxidation was performed employing the electron-rich catalysts (**7**, **8**), formation of hydroxyl and TFE rebound product mixtures was obtained, again in line with the trends observed for the corresponding reactions performed in HFIP in the presence of AcOH. In particular, the oxidation of **S1** catalyzed by **8** delivered the alcohol and ketone products (**P1u-OH** and **P1-O**) in 40% combined yield, accompanied by 10% of **P1u-OCH**_**2**_**CF**_**3**_ and 5% of **P1r-OCH**_**2**_**CF**_**3**_, confirming that the formation of the cationic intermediate is disfavored by the use of such catalyst. Most importantly, the observed ether products are formed as single diastereoisomers, suggesting common mechanistic features for TFE and carboxylate rebound, indicating that also TFE can bind to the metal center acting by all means as a co-ligand.^16,22^ Accordingly, we propose that the 2,2,2-trifluoroethoxy group in the rearranged alcohol product observed in the oxidation of *ent*-trachyloban-19-oate in TFE (Figure 2) also derives from the transfer of the OCH_2_CF_3_ to a cationic intermediate.

### Substrate Generality

**Scheme 7:**
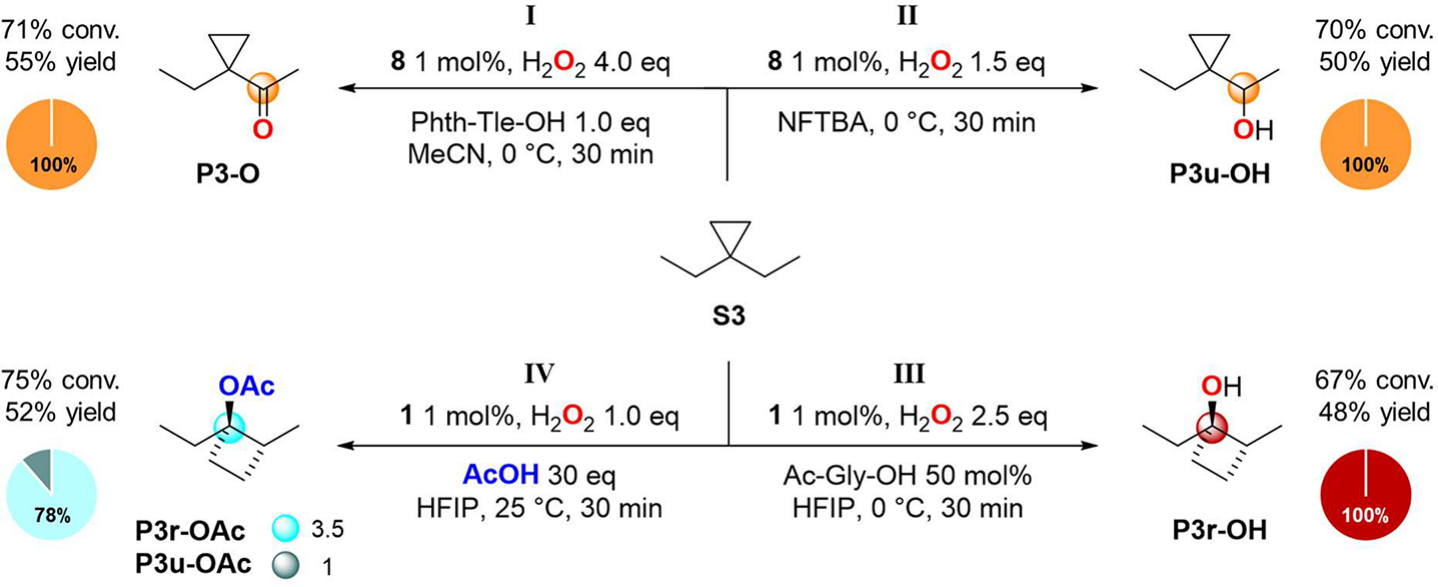
Oxidation of **S3** Pie charts refer to product selectivities while adjacent small circles to normalized product ratios.

Because **S1** is characterized by a very rigid structure with the *t*Bu group at C-6 that fixes the chair conformation, we investigated the generality of the trends observed in the oxidation of this substrate. To this end, we studied the possibility to promote alternative rebound products also in C(*sp*^3^)–H oxidations of 6-ethylspiro[2.5]octane (**S2**) and 1,1-diethylcyclopropane (**S3**), where an ethyl group at C-6 and an open structure, respectively, obviate this constraint. In order to probe the applicability of these concepts, we initially performed the oxidation of **S3** under the same reaction conditions reported in our previous work (Scheme 7).^16^

Pleasantly, we observed that the previously described conditions (catalyst, carboxylic acid co-ligand, solvent, and temperature) for governing chemoselectivity in the oxidation of **S1** translate in a consistent manner when applied to **S3**. Indeed, the unrearranged ketone (**P3-O**) and alcohol (**P3u-OH**) products derived from the hydroxyl transfer were obtained in 55% and 50% yield, respectively, without formation of products deriving from primary C–H bond oxidation (paths I and II, respectively). On the other hand, by performing the oxidation of **S3** in HFIP using **1** as catalyst and *N*-acetylglycine (Ac-Gly-OH) as co-ligand, the formation of 1-ethyl-2-methylcyclobutan-1-ol (**P3r-OH**) was observed in 48% yield as single product (path III). Very interestingly, the formation of **P3r-OH** clearly indicates that under mild reaction conditions C–H oxidation can proceed exclusively via the cationic pathway, expanding the applicability of this procedure to conformationally free cyclopropane derivatives. By replacing Ac-Gly-OH with AcOH, predominant formation of 1-ethyl-2-methylcyclobutyl acetate (**P3r-OAc**) in 41% yield and 78% selectivity was observed, accompanied by 1-(1-ethylcyclopropyl)ethyl acetate (**P3u-OAc**) in 11% yield (path IV). The ∼3-fold increase in acetate ester ratio observed going from **S1** (**P1r-OAc**/**P1u-OAc** = 1.2) to **S3** (**P3r-OAc**/**P3u-OAc** = 3.7) suggests that substrate rigidity impacts the carboxylate rebound pathway. Most interestingly, under these reaction conditions, hydroxylation products were not observed, evidencing that also in the oxidation of **S3** carboxylate transfer is solely responsible for product formation. The same behavior was observed when conditions **I**–**IV** were applied to the oxidation of 1,1-dipropylcyclopropane (**S4**) and spiro[2.5]heptane (**S5**) (see Schemes S7 and S8), showing the general applicability of these concepts to 1,1-dialkylcyclopropane and spiro[2.5]heptane derivatives.

**Scheme 8:**
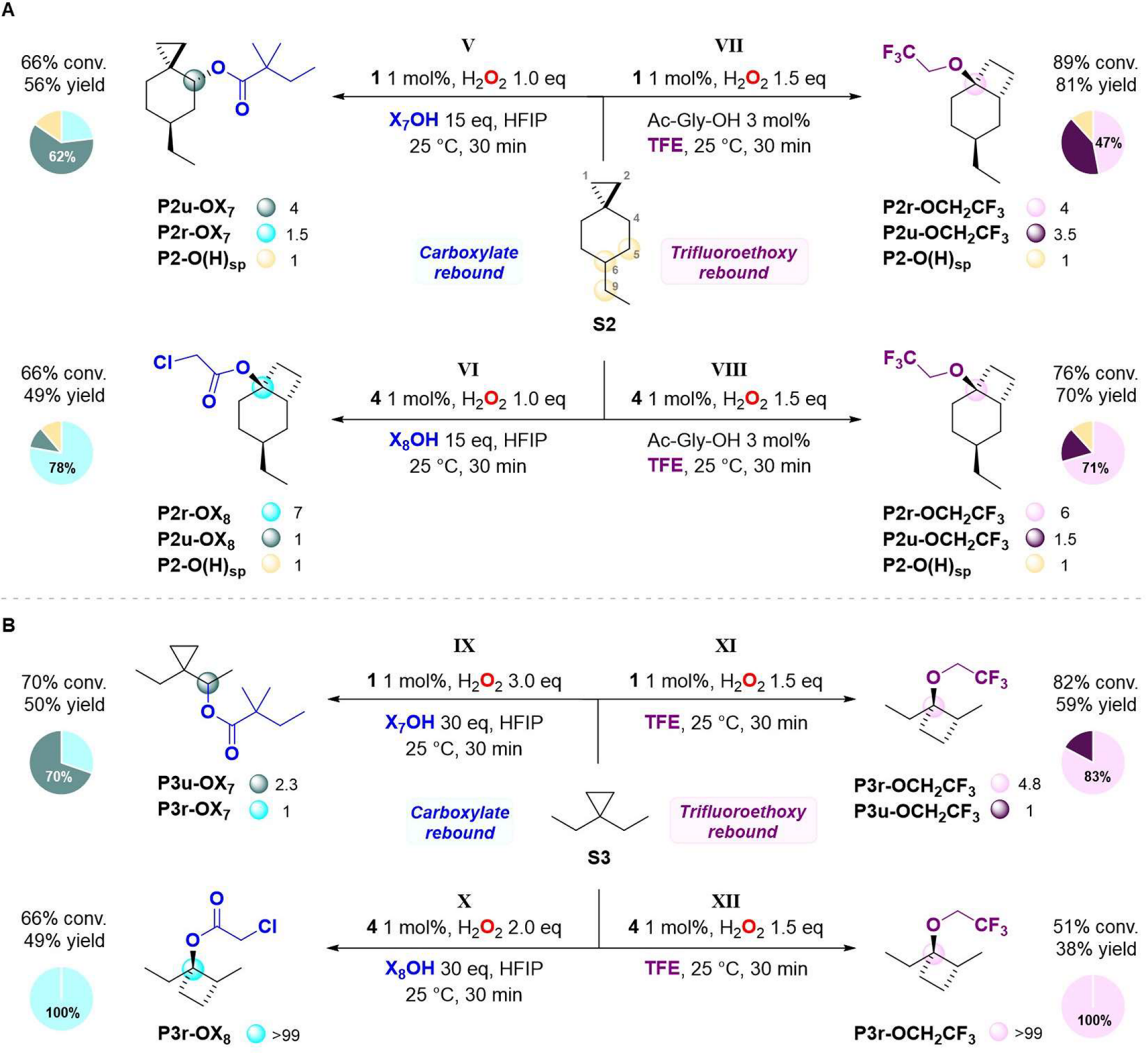
Oxidation of (A) **S2** and (B) **S3** The structures of the oxygenation products at C-5, C-6, and C-9 of **S3** (sp = side products) are reported in Tables S6 and S7. Pie charts refer to product selectivities while adjacent small circles refer to normalized product ratios.

Employing the optimal catalyst and reaction medium composition determined for **S1** (Schemes 5 and 6), carboxylate and TFE transfer in **S2** and **S3** were then demonstrated (Scheme 8) (see Tables S7–S10).

The oxidation of **S2** with **1** and **X**_**7**_**OH** in HFIP provided the unrearranged ester **P2u-OX**_**7**_ as the major product in 35% yield and 62% selectivity, accompanied by rearranged ester **P2r-OX**_**7**_ in 14% yield and oxygenation products at C-5, C-6, and C-9 (**P2-O(H)**_**sp**_) in 7% combined yield (Scheme 8A, path V). On the other hand, when the same reaction was performed with **4** and **X**_**8**_**OH**, predominant formation of the rearranged chloroacetate ester **P2r-OX**_**8**_ in 38% yield and 78% selectivity was observed (path VI). A similar outcome was observed when the oxidation of **S2** was performed in TFE and in the presence of 1 mol % of **1** and **4** (paths VII and VIII). With **1**, rearranged ether **P2r-OCH**_**2**_**CF**_**3**_ was obtained in 38% yield and 47% selectivity over **P2u-OCH**_**2**_**CF**_**3**_ (33% yield) and **P2-O(H)**_**sp**_ (10% yield). The oxidation of **S2** performed in the presence of **4** led to an increase in yield up to 50% and 71% selectivity for **P2r-OCH**_**2**_**CF**_**3**_. These results point toward the generality of these concepts, where access to alternative rebound pathways in C(*sp*^3^)–H oxidation requires the presence of an adjacent cyclopropyl group but is independent of the alkyl substituent at C-6 of the cyclohexane ring.

These optimized reaction conditions were then applied to the oxidation of **S3** (Scheme 8B). When the oxidation of **S3** was performed with **1** and **X**_**7**_**OH** in HFIP, predominant formation of **P3u-OX**_**7**_ in 35% yield and 70% selectivity over **P3r-OX**_**7**_ (15% yield) was observed (path IX). Instead, the oxidation performed with **4** and **X**_**8**_**OH** in HFIP led to the formation of the rearranged chloroacetate ester (**P3r-OX**_**8**_) in 49% yield, with no other oxygenation product being detected (path X). Very interestingly, this result indicates that manganese-catalyzed C(*sp*^3^)–H oxidation of **S3** can occur exclusively via cationic paths. When the oxidation of **S3** was performed using **1** in TFE, predominant formation of **P3r-OCH**_**2**_**CF**_**3**_ in 49% yield and 83% selectivity was observed, accompanied by the formation of **P3u-OCH**_**2**_**CF**_**3**_ in 10% yield (path XI). By replacement of catalyst **1** with **4**, the oxidation of **S3** led to the formation of **P3r-OCH**_**2**_**CF**_**3**_ in 38% yield as a single product (path XII), in parallel with the behavior observed for the chloroacetate rebound.

**Scheme 9:**
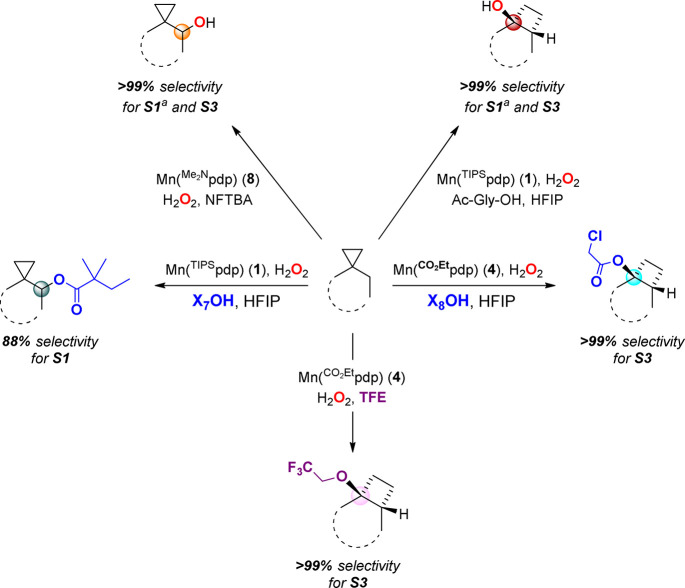
Summary of the Competitive Rebound Pathways Observed in the Oxidation of Substrates **S1**–**S3** under Different Experimental Conditions Reference (16).

From a synthetic perspective the selective formation of cyclobutyl chloroacetate ester **P3r-OX**_**8**_ and 2,2,2-trifluoroethyl ether **P3r-OCH**_**2**_**CF**_**3**_, which can be easily modified by reported methodologies,^23^ represents a breakthrough, providing straightforward access to relevant structures from commercially available linear alkyl ketones. Most importantly, the single step access to rearranged alcohols, esters, and ethers from cyclopropane containing substrates **S1**–**S3**, summarized in Scheme 9, expands the family of reactions that combine C–H and C–C bond cleavage.^24^ The synthetic value of the reaction is best evidenced by the expedient access they provide to complex structures with complete stereocontrol.^23,25^

## Conclusions

Taken together, the catalytic methodology described herein for C(*sp*^3^)–H bond oxidation of **S1** with H_2_O_2_ promoted by manganese complexes provides access to alternative rebound pathways (carboxylate and solvent (TFE)) enabling, for the first time, full divergence from the canonical hydroxylation reaction. By carefully tuning catalyst electronics and carboxylic acid co-ligand sterics and electronics, selectivity toward the unrearranged and rearranged carboxylate ester products could be modulated. The use of an electron-poor catalyst such as Mn(^CO_2_Et^pdp) (**4**) favors the formation of rearranged esters, increasing the relative importance of cationic pathways in governing product formation. On the change of carboxylic acid structure, selectivity toward the rearranged ester product was observed to increase by a factor 45 on going from 2,2-dimethylbutanoic to chloroacetic acid, suggesting that the use of an unhindered acid promotes transfer to the more sterically congested center of the delocalized cationic intermediate. In TFE, oxidation of **S1** leads to the formation of unrearranged and rearranged TFE rebound ether products with complete control over diastereoselectivity, indicating that also this solvent can act as a co-ligand that is transferred with the same mechanistic features observed for the carboxylic acids. Finally, the optimal conditions developed for **S1** could be extended to the oxidation of cyclohexane, cyclopentane, and conformationally free cyclopropyl derivatives (**S2**–**S5**), pointing toward the generality of these findings and highlighting the important role of cyclopropyl groups in activating adjacent C–H bonds and promoting selective functionalization at these sites via cationic pathways. Along this line, the observation that in the oxidation of 1,1-diethylcyclopropane (**S3**), rearranged chloroacetate ester and 2,2,2-trifluoroethyl ether were obtained as single products points toward reactions that proceed exclusively through a cationic intermediate. These results deserve attention also from a synthetically oriented perspective. Considering that cyclopropane moieties can be easily installed on ubiquitous carbonyl groups and that, where available, this transformation determines an inversion in the polarity of the α-C(*sp*^3^)–H bonds, these oxidative transformations, initiated by polarity matched HAT from these bonds, can provide straightforward access to relevant cyclobutyl structures.

## Supporting Information Available

Experimental details for the preparation of metal complexes and substrates, catalytic reactions, and details on isolation and characterization of the reaction products (PDF)
