# Supplementary material for: Catalyst and Medium Control over Rebound Pathways in Manganese-Catalyzed Methylenic C–H Bond Oxidation
Source: J Am Chem Soc. 2024 Mar 20;146(13):8904–14. doi: 10.1021/jacs.3c11555 (PMC10996012; doi:10.1021/jacs.3c11555)
Supplement: Supplementary file 1 — ja3c11555_si_001.pdf [file ja3c11555_si_001.pdf]

Supporting information

Catalyst and Medium Control Over Rebound Pathways in Manganese-Catalyzed Methylenic C-H Bond Oxidation

Marco Galeotti,[1] Massimo Bietti,[2,*] and Miquel Costas[1,*]

E-mail: bietti@uniroma2.it, miquel.costas@udg.edu

[1] *QBIS Research Group, Institut de Química Computacional i Catàlisi (IQCC) and Departament de Química, Universitat de Girona, Campus Montilivi, Girona E-17071, Catalonia, Spain*

[2] *Dipartimento di Scienze e Tecnologie Chimiche, Università "Tor Vergata", Via della Ricerca Scientifica, 1 I-00133 Rome, Italy.*

CONTENTS

1. Experimental Section S3
  1.1. Instrumentation S3
  1.2. Materials S3

2. Synthesis of the substrates S4

3. Synthesis of the complexes S7
  3.1. Synthesis of the ligands S7
  3.2. Synthesis of the manganese catalysts S9

4. Oxidation with H_2O_2 catalyzed by Mn complexes S11
  4.1. General procedure S11
  4.2. Optimization of the reaction conditions S11
  4.2. Oxidation of S2-S5 .... S16

5. Isolation and characterization of the oxidation products S19
  5.1. Acylation of alcohols P1u-OH and P1r-OH S19
  5.2. Scale-up oxidation of S1-S3 .... S23

6. NMR spectra S30
  6.1. NMR spectra of the substrates S30
  6.2. NMR spectra of the ligands S32
  6.3. NMR spectra of the isolated products S38

7. HRMS spectra of the catalysts S59

8. References S61

1. Experimental section

1.1. Instrumentation

Gas-chromatographic analyses were carried out for the oxidation reaction with H_2O_2 catalyzed by manganese-oxo complexes using an Agilent 7820A gas chromatograph equipped with an HP-5 capillary column 30m x 0.32 mm x 0.25 μm and a flame ionization detector. GC-MS analyses are performed on an Agilent 7890A gas chromatograph equipped with an HP-5MS capillary column (30 m x 0.25 mm x 0.25 μm) interfaced with an Agilent 5975X mass spectrometer. NH_3 was used as the ionization gas. NMR spectra were taken on a Bruker Ultrashield AVANCE III400 or on a Bruker Ultrashield ASCEND Nanobay spectrometer using standard conditions. Spectra were referenced to the residual proton solvents peaks or TMS (tetramethylsilane). High resolution mass spectra (HRMS) were recorded on a Bruker MicroTOF-Q IITM instrument with an ESI source and a quadrupole analyzer at Serveis Tècnics of the University of Girona. Samples were introduced into the mass spectrometer ion source by direct infusion through a syringe pump and were externally calibrated using sodium formate.

1.2. Materials

Reagents and solvents used are of commercially available reagent quality unless stated otherwise and are purchased from SDS, Aldrich, Scharlab and Fluorochem. Sigma-Aldrich HPLC-grade acetonitrile was employed for oxidation catalysis.

The hydrogen peroxide solutions employed in the oxidation reactions were prepared by diluting commercially available hydrogen peroxide (30% H_2O_2 solution in water, Aldrich) in MeCN and in fluorinated solvents to achieve a ~1.0 M final concentration. All reagents used are of the highest commercial quality available unless stated otherwise.

2. Synthesis of the substrates

6-*tert*-butylspiro[2.5]octane (**S1**) and 6-ethylspiro[2.5]octane (**S2**) was synthetized over two steps (Wittig olefination and Simmons-Smith cyclopropanation) according to reported procedures (**Scheme S1**).[1] 1,1-diethylcyclopropane (**S3**), 1,1-dipropylcyclopropane (**S4**) and spiro[2.4]heptane (**S5**) was synthetized via Simmons-Smith cyclopropanation of the commercially available alkenes.

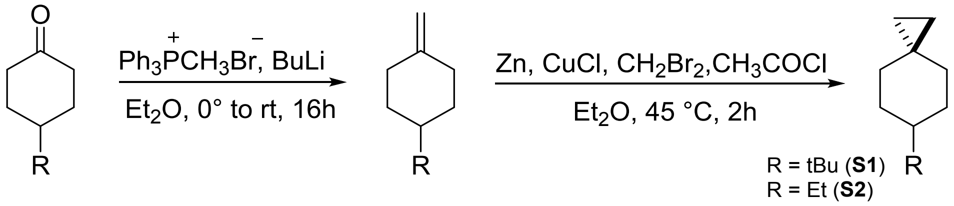

Scheme S1. Synthesis of 6-alkylspiro[2.5]octane (**S1** and **S2**).

<u>*Wittig olefination of 4-R-butylcyclohexanone*</u> (**R = *t*Bu, Et**)
13.7 g of methyltriphenylphosphonium bromide (0.0384 mol, 1.2 equiv) and 100 mL of anhydrous diethyl ether were introduced in a 250 mL three-necked round-bottom flask equipped with a pressure-equalizing dropping funnel and kept under nitrogen. The reaction mixture was cooled at 0°C and 23 mL of 1.6 M *n*-butyllithium solution in hexane (0.0368 mol, 1.15 equiv) were added dropwise in 15 minutes under vigorous stirring. When the solution becomes orange 5 g of 4-*tert*-butylcyclohexanone (0.032 mol, 1.0 equiv) or 4 g 4-ethylcyclohexanone (0.032 mol, 1.0 equiv) diluted in 10 mL of anhydrous diethyl ether, were added dropwise and the reaction mixture was kept stirring under a nitrogen atmosphere for 16 hours at 25 °C. After complete substrate conversion (monitored by TLC or GC analysis), the reaction mixture was cooled at 0°C, quenched with 100 mL of a saturated ammonium chloride aqueous solution and extracted with two portions of diethyl ether (2 x 75 mL). The combined organic phases are dried over anhydrous sodium sulfate, the solvent is removed at reduced pressure and the product is purified by flash chromatography over silica gel in pure *n*-hexane.
4.0 g of 4-*tert*-butylmethylenecyclohexane (0.026 mol, 81% yield) were collected as colorless liquid. Spectroscopic data match those previously reported.[1] ^{1}H-NMR (400 MHz, $CDCl_3$) δ, ppm: 4.58 (t, 2H), 2.36 – 2.30 (m, 2H), 1.99 (t, 2H), 1.87 (d, 2H), 1.17 –1.00 (m, 3H), 0.85 (s, 9H).
3.0 g of 4-ethylmethylenecyclohexane (0.024 mol, 75% yield) were collected as colorless liquid. Spectroscopic data match those previously reported.[2] ^{1}H-NMR (400 MHz, $CDCl_3$) δ, ppm: 4.61 (t, 2H), 2.36 – 2.26 (m, 2H), 2.09 – 1.97 (m, 2H), 1.88 – 1.80 (m, 2H), 1.37 – 1.20 (m, 3H), 1.08 – 0.92 (m, 2H), 0.91 (t, 3H).

<u>*Simmons-Smith cyclopropanation of 4-alkyl-methylenecyclohexane*</u> (**R = *t*Bu, Et**)
6.8 g of zinc (0.104 mol, 4 equiv), 1.03 g of copper (I) chloride (0.0104 mol, 0.4 equiv), 1.8 mL of dibromomethane (0.026 mol, 1.0 equiv) and 15 mL of diethyl ether were introduced into a 100 mL three necked round-bottom flask, equipped with a condenser system and a pressure-equalizing dropping funnel. After addition of 148 µL of acetyl chloride (0.00208 mol, 0.08 equiv), the flask was placed in a pre-heated oil bath (45-50 °C) under vigorous reflux and stirring. When the solution was turned dark (about 10 minutes), 4 g of 4-*tert*-butylmethylenecyclohexane (0.026 mol, 1.0 equiv) or 3.2 g of 4-ethylmethylenecyclohexane (0.026 mol, 1.0 equiv) diluted in 5 mL of diethyl ether, was added dropwise for 15 minutes. Additional 3.7 mL of dibromomethane (0.052 mol, 2.0 equiv) diluted

in 5 mL of diethyl ether were added dropwise for 30 minutes under manageable reflux. When substrate was fully converted (generally 2 hours after the addition of all the reagents), the flask was cooled in an ice bath and 25 mL of a saturated ammonium chloride aqueous solution were added dropwise (the reaction is strongly exothermic) through the dropping funnel. The residual solid zinc was removed by vacuum filtration and the aqueous layer of the filtrate was washed twice with *n*-pentane (2 x 25 ml). The combined organic layers were washed twice with a 2.5 M sodium hydroxide aqueous solution (2 x 50 mL), one time with brine (50 mL) and were dried over anhydrous sodium sulfate. Due to product volatility *n*-pentane and diethyl ether were carefully removed by Hempel fractional distillation and a controlled vacuum distillation of the remaining oil gave the pure cyclopropyl derivative.

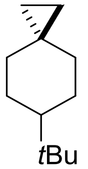

S1

2.0 g of 6-*tert*-butylspiro[2.5]octane (**S1**, 0.012 mol, 46% yield) were collected as colorless liquid. Spectroscopic data match those previously reported.[1] ^{1}H-NMR (400 MHz, $CDCl_3$) δ, ppm: 1.80 – 1.68 (m, 4H), 1.22 – 1.08 (m, 2H), 1.07 – 0.95 (m, 1H), 0.89 (m, 11H), 0.32 – 0.23 (m, 2H), 0.23 – 0.12 (m, 2H).

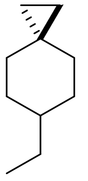

S2

1.91 g of 6-ethylspiro[2.5]octane (**S2**, 0.014 mol, 54% yield) were collected as colorless liquid. Spectroscopic data match those previously reported.[3] ^{1}H-NMR (400 MHz, $CDCl_3$) δ, ppm: 1.75 – 1.66 (m, 4H), 1.32 – 1.23 (m, 2H), 1.21 – 1.05 (m, 3H), 0.94 – 0.85 (m, 5H), 0.30 – 0.23 (m, 2H), 0.21 – 0.15 (m, 2H).

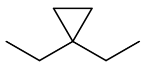

S3

1,1-diethylcyclopropane (**S3**) was synthetized via Simmons-Smith cyclopropanation of 3-methylenepentane according to the procedure reported above (0.074 mol scale). 2.0 g of **S3** (0.020 mol, 27% yield) were collected as colorless liquid. Spectroscopic data match those previously reported.[4] ^{1}H-NMR (400 MHz, $CDCl_3$) δ, ppm: 1.28 (q, J = 7.4 Hz, 4H), 0.89 (t, J = 7.4 Hz, 6H), 0.21 (s, 4H).

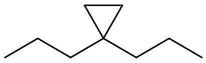

S4

1,1-dipropylcyclopropane (**S4**) was synthetized via Simmons-Smith cyclopropanation of 3-methyleneheptane according to the procedure reported above (0.040 mol scale). 1.6 g of **S4** (0.013 mol, 33% yield) were collected as colorless liquid. ^{1}H-NMR (400 MHz, $CDCl_3$) δ, ppm: 1.42 – 1.31 (m, 4H), 1.24 – 1.18 (m, 4H), 0.90 (t, J = 7.3 Hz, 6H), 0.21 (s, 4H). $^{13}C\{^{1}H\}$-NMR (400 MHz, $CDCl_3$) δ, ppm: 38.33, 19.74, 19.02, 14.47, 11.85.

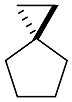

S5

Spiro[2.4]heptane (**S5**) was synthetized via Simmons-Smith cyclopropanation of methylenecyclopentane according to the procedure reported above (0.047 mol scale). 1.1 g of **S5** (0.012 mol, 26% yield) were collected as colorless liquid. ^{1}H-NMR (400 MHz, $CDCl_3$) δ, ppm: 1.76 – 1.69 (m, 4H), 1.55 – 1.48 (m, 4H), 0.44 (s, 4H). $^{13}C\{^{1}H\}$-NMR (400 MHz, $CDCl_3$) δ, ppm: 35.88, 26.47, 22.45, 12.92.

3. Synthesis of the complexes

The complexes Mn(^{TIPS}mcp),[5] Mn(^{TIPS}pdp) (**1**),[5] Mn(^{H}pdp) (**2**),[6] Mn(^{Cl}pdp) (**3**),[7] Mn(^{CO_2Et}pdp) (**4**),[7] Mn(^{DMM}pdp) (**7**),[7] Mn(^{Me_2N}pdp)[7] (**8**) were prepared according to the reported procedures (**Figure S1**). The synthesis of Mn(^{CF_3}pdp) (**5**), Mn($^{p\text{-}TIPS}$pdp) (**6**), is reported below.

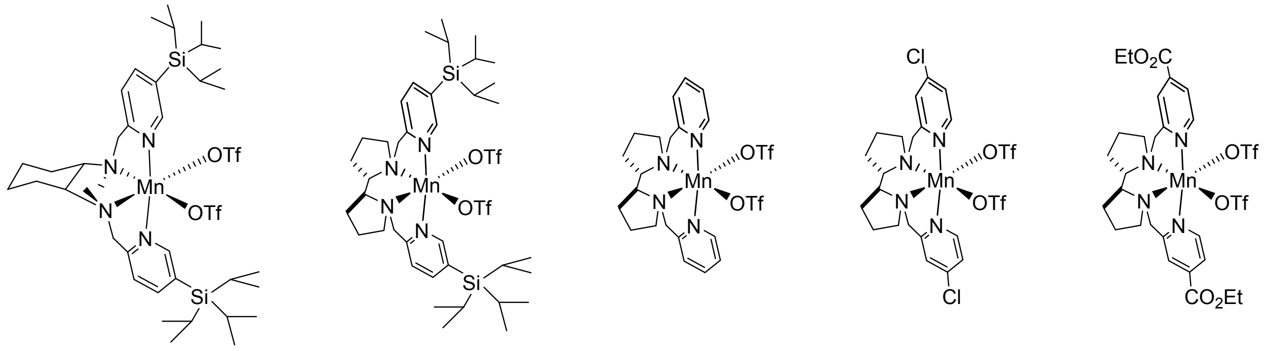

Mn(^{TIPS}mcp) **Mn(^{TIPS}pdp) (1)** **Mn(^{H}pdp) (2)** **Mn(^{Cl}pdp) (3)** **Mn(^{CO_2Et}pdp) (4)**

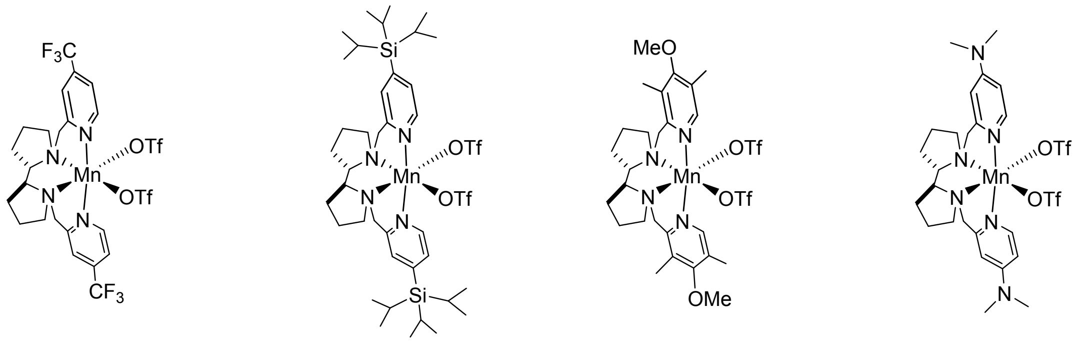

Mn(^{CF_3}pdp) (5) **Mn($^{p\text{-}TIPS}$pdp) (6)** **Mn(^{dMM}pdp) (7)** **Mn(^{Me_2N}pdp) (8)**

Figure S1. Structures of the manganese catalysts employed in this work.

3.1 Synthesis of the ligands

1. Synthesis of (*R*,*R*)-^{CF_3}pdp. (*R*,*R*)-^{CF_3}pdp was prepared according to slightly modification of reported procedures (**Scheme S2**).[5]

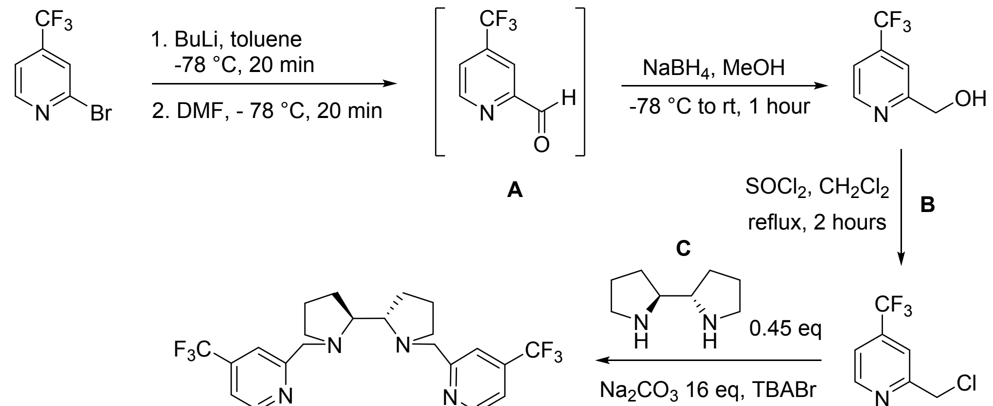

(*R*,*R*)-^{CF_3}pdp

Scheme S2. Synthesis of (*R*,*R*)-^{CF_3}pdp.

A. To a solution of 2-bromo-4-(trifluoromethyl)pyridine (1.5 g, 6.64 mmol) in dry toluene (20 ml), at -78 °C, was added n-butyllithium (5.2 ml, 8.3 mmol, 1.6 M) dropwise under nitrogen. After the addition, the reaction was stirred at -78 °C for 10 minutes and *N*,*N*-dimethylformamide (0.77 ml, 9.95 mmol) was added dropwise at -78 °C and stirred for 10 minutes at -78 °C before sodium borohydride (0.5 g, 13.3 mmol) and methanol (3.75 ml) were added. The reaction was warmed to room temperature and stirred for 1 h. The reaction was quenched with aqueous ammonium chloride solution and extracted with ethyl acetate (30 ml x 2). The combined organic phases were washed with brine (30 ml), dried over $MgSO_4$, filtered and concentrated to give (4-(trifluoromethyl)pyridin-2-yl)methanol (962 mg, 5.43 mmol, 82% yield) as a pale yellow solid. ^{1}H-NMR (400 MHz, $CDCl_3$) δ, ppm: 8.73 (d, J = 5.1 Hz, 1H), 7.59 (d, J = 1.7 Hz, 1H), 7.43 (dd, J = 5.2, 1.6 Hz, 1H), 4.86 (s, 2H), 4.05 (bs, 1H). ^{13}C{^{1}H}-NMR (400 MHz, $CDCl_3$) δ, ppm: 161.38, 149.64, 139.11, 122.72, 118.01, 116.37, 64.31. ^{19}F-NMR (400 MHz, $CDCl_3$) δ, ppm: -65.94. HRMS (ESI-MS) *m/z* calculated for $C_7H_6F_3NO$ $[M+H]^+$ 178.0474, found 178.0479.

B. To a solution of (4-(trifluoromethyl)pyridin-2-yl)methanol (870.5 mg, 4.91 mmol) in dichloromethane (20 mL) was added thionyl chloride (1.8 ml, 24.6 mmol) dropwise at room temperature. The reaction was stirred at 60 °C for 2 h and was concentrated (reflux). At this point, the solvent was removed under reduced pressure to provide 2-(chloromethyl)-4-(trifluoromethyl)pyridine hydrochloride (921 mg, 3.97 mmol, 81% yield) as a yellow solid. ^{1}H-NMR (400 MHz, CD_3OD) δ, ppm: 8.88 (d, J = 5.3 Hz, 1H), 8.03 (s, 1H), 7.83 (d, J = 5.4 Hz, 1H), 4.88 (s, 2H). ^{13}C{^{1}H}-NMR (400 MHz, CD_3OD) δ, ppm: 157.54, 149.06, 141.02, 127.39, 122.28, 119.91, 43.77. ^{19}F-NMR (400 MHz, CD_3OD) δ, ppm: -67.46. HRMS (ESI-MS) *m/z* calculated for $C_7H_5F_3NCl$ $[M+H]^+$–[HCl] 196.0135, found 196.0139.

C. (2*R*,2'*R*)-2,2'-bipyrrolidine (81.4 mg, 0.58 mmol), 2-(chloromethyl)-4-(trifluoromethyl)pyridine hydrochloride (300 mg, 1.29 mmol), Na_2CO_3 (2.2 g, 20.6 mmol) and TBABr (10 mg) was dissolved in anhydrous CH_3CN (20 ml). The reaction mixture was refluxed overnight under N_2. At this point, the crude reaction was filtered and the solvent was evaporated under reduced pressure. NaOH 2M (15 ml) was added and the organic layer was separated from the basic aqueous layer. The aqueous layer was extracted with CH_2Cl_2 (3 x 15 ml) and the organic layer were combined and dried over $MgSO_4$. The obtained brown oil was purified by silica column (CH_2Cl_2:MeOH:NH_3 95:4:1) and the collected fractions were removed under reduced pressure to provide 220.7 mg (0.481 mmol, 83% yield) of a yellow oil. ^{1}H-NMR (400 MHz, $CDCl_3$) δ, ppm: 8.67 (d, J = 5.1 Hz, 2H), 7.63 (s, 2H), 7.41 – 7.29 (m, 2H), 4.30 (d, J = 15.1 Hz, 2H), 3.66 (d, J = 15.1 Hz, 2H), 3.03 (dt, J = 9.5, 4.6 Hz, 2H), 2.82 (d, J = 7.3 Hz, 2H), 2.28 (q, J = 8.2 Hz, 2H), 1.97 – 1.62 (m, 8H). ^{13}C{^{1}H}-NMR (400 MHz, $CDCl_3$) δ, ppm: 162.45, 149.28 138.60, 122.87, 118.03, 117.23, 66.23, 61.11, 55.34, 26.46, 23.74. ^{19}F-NMR (400 MHz, CD_3OD) δ, ppm: -65.83. HRMS (ESI-MS) *m/z* calculated for $C_{22}H_{24}F_6N_4$ $[M+H]^+$ 459.1978, found 459.1982.

2. Synthesis of (*R*,*R*)-$^{p\text{-TIPS}}$pdp. 2-(chloromethyl)-4-(triisopropylsilyl)pyridine hydrochloride was prepared according to reported procedures.[5] The synthesis of (*R*,*R*)-$^{p\text{-TIPS}}$pdp is reported below (**Scheme S3**).

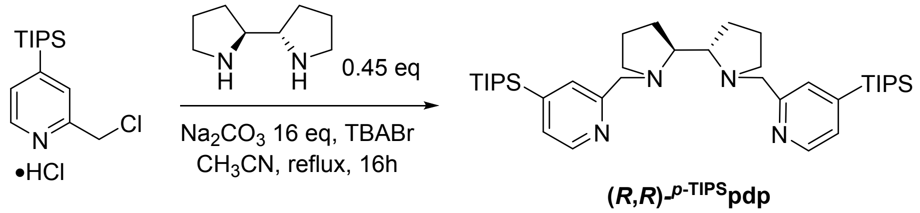

Scheme S3. Synthesis of (*R*,*R*)-$^{p\text{-TIPS}}$pdp.

(2*R*,2'*R*)-2,2'-bipyrrolidine (59 mg, 0.421 mmol), 2-(chloromethyl)-4-(triisopropylsilyl)pyridine hydrochloride (300 mg, 0.936 mmol), Na_2CO_3 (992 mg, 9.36 mmol) and TBABr (10 mg) was dissolved in anhydrous CH_3CN (15 ml). The reaction mixture was refluxed overnight under N_2. At this point, the crude reaction was filtered and the solvent was evaporated under reduced pressure. NaOH 2M (15 ml) was added and the organic layer was separated from the basic aqueous layer. The aqueous layer was extracted with CH_2Cl_2 (3 x 15 ml) and the organic layer were combined and dried over $MgSO_4$. The obtained brown oil was purified by silica column (CH_2Cl_2:MeOH:NH_3 95:4:1) and the collected fractions were removed under reduced pressure to provide 142 mg (0.224 mmol, 53% yield) of a yellow oil. ^{1}H-NMR (400 MHz, CD_3OD) δ, ppm: 8.41 (d, J = 4.9 Hz, 2H), 7.68 (s, 2H), 7.39 (dd, J = 4.9, 1.1 Hz, 2H), 4.35 (d, J = 14.4 Hz, 2H), 3.59 (d, J = 14.1 Hz, 2H), 2.91 (d, J = 43.1 Hz, 4H), 2.28 (s, 2H), 1.81 (d, J = 63.3 Hz, 8H), 1.48 – 1.39 (m, 6H), 1.08 (m, 36H). ^{13}C{^{1}H}-NMR (400 MHz, CD_3OD) δ, ppm: 146.98, 146.40, 129.64, 128.80, 128.50, 66.26, 60.66, 54.89, 26.64, 23.29, 17.45, 10.30. HRMS (ESI-MS) *m/z* calculated for $C_{38}H_{66}N_4Si_2$ $[M+H]^+$ 635.4899, found 635.4890.

3.2 Synthesis of the manganese catalysts

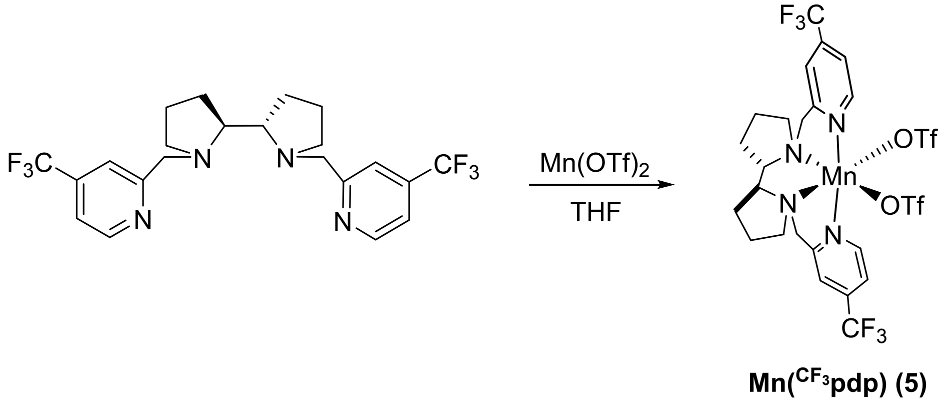

Scheme S4. Synthesis of Mn(^{CF_3}pdp) (**5**).

Under N_2 atmosphere, $Mn(CF_3SO_3)_2$ (77 mg, 0.218 mmol) was added to a vigorously stirred solution of (*R*,*R*)-^{CF_3}pdp (100 mg, 0.218 mmol) in THF (2 mL). After a few seconds the solution became cloudy and a white precipitate appeared. After stirring for 4 hours the solution was filtered off and the resultant white solid dried under vacuum. This solid was dissolved in CH_2Cl_2 (3 mL) and the solution filtered off through Celite©. The solid was crystallized by layering CH_2Cl_2 solution of the complex with hexane to yield the desired white complex (114 mg, 0.14 mmol, 64% yield). Analysis calculated for $C_{24}H_{24}F_{12}MnN_4O_6S_2$: C, 37.09; H, 2.99; N, 5.19 %. Found: C, 37.29; H, 3.28; N, 6.30

%. HRMS (ESI-MS) *m/z* calculated for $C_{23}H_{24}F_9MnN_4O_3S$ [M-OTf]$^+$: 662.0800, found: 662.0815 (HMRS spectrum is shown in section **7**).

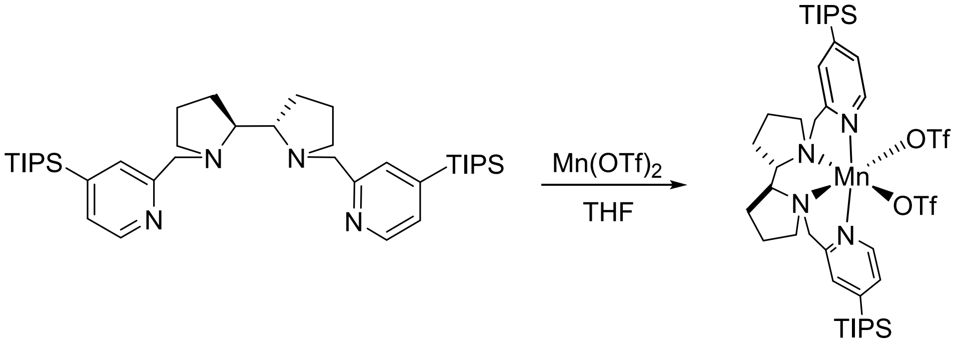

Mn($^{p\text{-TIPS}}$pdp) (6)

Scheme S5. Synthesis of Mn($^{p\text{-TIPS}}$pdp) (**5**).

Under N_2 atmosphere, $Mn(CF_3SO_3)_2$ (66 mg, 0.187 mmol) was added to a vigorously stirred solution of (*R*,*R*)-^{TIPS}pdp (119 mg, 0.187 mmol) in THF (2 mL). After a few seconds the solution became cloudy and a white precipitate appeared. After stirring for 4 hours the solution was filtered off and the resultant white solid dried under vacuum. This solid was dissolved in CH_2Cl_2 (3 mL) and the solution filtered off through Celite©. The solid was crystallized by layering CH_2Cl_2 solution of the complex with hexane to yield the desired white complex (84 mg, 0.085 mmol, 45% yield). Analysis calculated for $C_{40}H_{66}F_6MnN_4O_6S_2Si_2$: C, 49.93; H, 6.75; N, 4.26 %. Found: C, 46.37; H, 5.88; N, 4.32 %. HRMS (ESI-MS) *m/z* calculated for $C_{39}H_{66}F_3MnN_4O_3S_1Si_2$ [M-OTf]$^+$: 838.3721, found: 838.3724 (HMRS spectrum is shown in section **7**).

4. Oxidation with H_2O_2 catalyzed by Mn complexes

4.1. General procedure

The following procedure was used for the aliphatic C-H bond oxidations of **S1**-**S5** with H_2O_2 catalyzed by Mn catalysts unless otherwise noted.
A MeCN, TFE (2,2,2-trifluoroethanol) HFIP (1,1,1,3,3,3-hexafluoro-2-propanol) or NFTBA (nonafluoro-tert-butyl alcohol) solution (400 µL, 0.125 M) of the substrate (50 µmol, 1.0 equiv) and the Mn catalyst (0.5 µmol, 1 mol%) was prepared in a 10 mL vial equipped with a stirring bar and thermostated at T = 0 °C (ice bath) or 25 °C. The carboxylic acid (2-15 equiv) was added directly to the solution. Then 1.0 M H_2O_2 solution in the pertinent solvent was directly added by syringe pump over 30 minutes. At this point 0.5 equiv of internal standard (biphenyl) was added and the solution was quickly filtered through a small silica and basic alumina plug, which was subsequently rinsed with 2 x 1 mL of EtOAc. GC analysis of the solution afforded the substrate conversions and product yields relative to the internal standard integration. Calibration curves were obtained using an authentic sample of the pure compounds prepared by scale-up catalysis or alternative procedures.

4.2. Optimization of the reaction conditions

Unless otherwise noted, the optimization experiments for the oxidation of **S1** were done following the general procedure reported above. Catalyst enantiomers were used interchangeably.

Table S1. Oxidation of **S1** with Mn(^{TIPS}mcp) and Mn(^{TIPS}pdp) (**1**).[a]

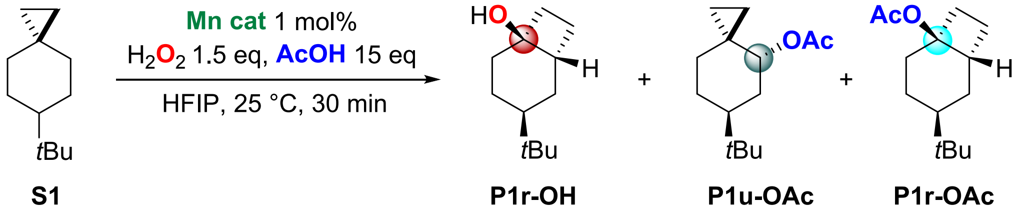

Entry	Mn cat	% conv	% product yields		
			P1r-OH	P1u-OAc	P1r-OAc
1[b]	Mn(^{TIPS}mcp)	90	3	41	33
2[c]	Mn(^{TIPS}pdp)	65	4	21	32

[a]Conversion and product yields were determined by GC and were averaged over at least two independent experiments. [b]Ref. 1. [c]1.0 equiv. of H_2O_2 were used.

Table S2. Screening of the carboxylic acid additive amount.[a]

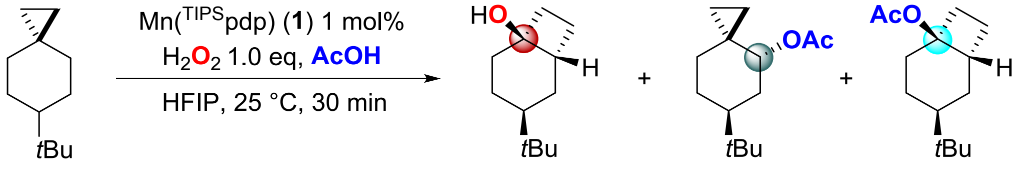

S1 **P1r-OH** **P1u-OAc** **P1r-OAc**

Entry	AcOH equiv	% conv	% product yields		
			P1r-OH	P1u-OAc	P1r-OAc
**1**	2	62	20	10	24
**2**	5	60	10	10	32
**3**	10	67	6	16	36
**4**	15	65	4	21	32
**5**	30	70	-	28	34

[a]Conversion and product yields were determined by GC and were averaged over at least two independent experiments.

Table S3. Screening of nucleophile additives.[a]

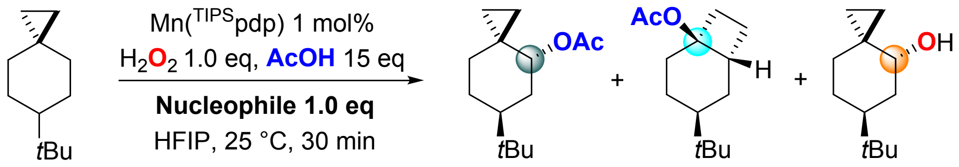

S1 **P1u-OAc** **P1r-OAc** **P1u-OH**

Entry	Nucleophile	% conv	% product yields		
			P1u-OAc	P1r-OAc	P1u-OH
**1**[b]	-	65	21	33	-
**2**	Bu_4NCl	19	3	-	5
**3**	Bu_4NBr	42	9	-	10
**4**	Bu_4NHSO_4	47	3		4
**5**	$Bu_4NH_2PO_4$	-	-	-	-
**6**	Bu_4NOAc	70	29	6	30
**7**	Bu_4NCN	96	36	5	36
**8**	Bu_4NN_3	94	44	5	35

[a]Conversion and product yields were determined by GC and were averaged over at least two independent experiments. [b]Additional 4% yield of P1r-OH (see Table S2, entry 4)

Table S4. Screening of catalyst electronics in HFIP.[a]

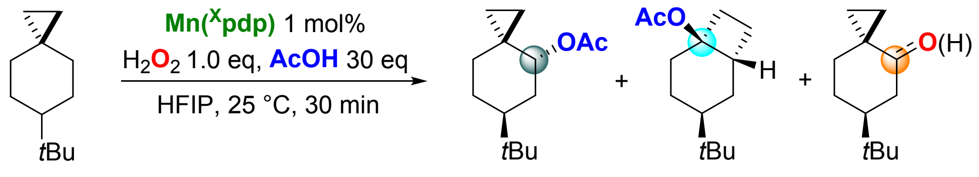

Entry	Mn(^{X}pdp)	% conv	% product yields		
			P1u-OAc	P1r-OAc	P1u-O(H)
**1**	Mn(^{TIPS}pdp) (**1**)	70	28	34	-
**2**	Mn(^{H}pdp) (**2**)	22	8	13	-
**3**	Mn(^{Cl}pdp) (**3**)	42	7	26	-
**4**	Mn(^{CO_2Et}pdp) (**4**)	47	8	32	-
**5**	Mn(^{CF_3}pdp) (**5**)	33	5	21	-
**6**	Mn($^{p\text{-}TIPS}$pdp) (**6**)	53	23	24	-
**7**	Mn(^{DMM}pdp) (**7**)	75	36	22	12
**8**	Mn(^{Me_2N}pdp) (**8**)	72	42	14	16

[a]Conversion and product yields were determined by GC and were averaged over at least two independent experiments.

Table S5. Screening of carboxylic acid co-ligands.[a]

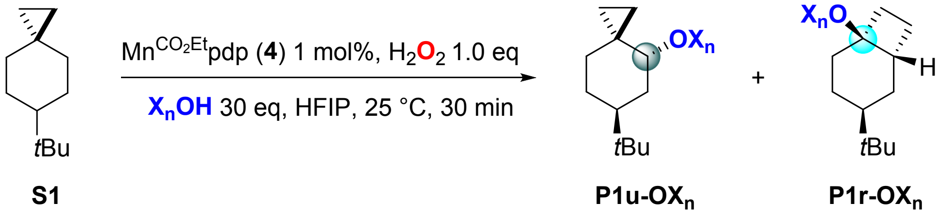

Entry	X_nOH	% conv	% product yields: P1u-OX_n	% product yields: P1r-OX_n
1	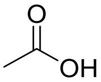	47	8	32
2	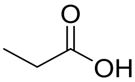	49	16	29
3	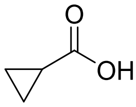	55	19	34
4	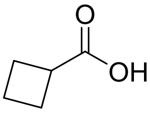	38	9	25
5	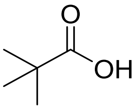	65	50	9
6	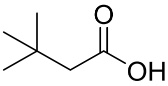	36	15	20
7[b]	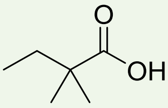	85	63	9
8	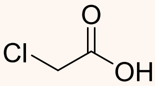	63	7	44

[a]Conversion and product yields were determined by GC and were averaged over at least two independent experiments. [b]Mn(^{TIPS}pdp) 1 mol% and H_2O_2 3.0 equiv were used.

Table S6. Screening of catalyst electronics in TFE.[a]

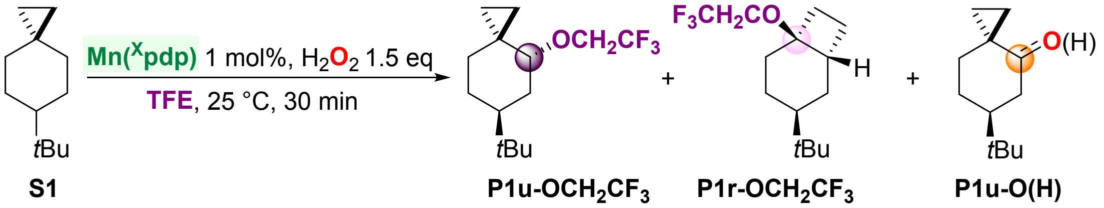

Entry	Mn(^{X}pdp)	% conv	% product yields		
			P1u-OCH$_2$CF$_3$	P1r-OCH$_2$CF$_3$	P1-O(H)
**1**	Mn(^{TIPS}pdp) (**1**)	65	34	26	-
**2**	Mn(^{H}pdp) (**2**)	64	24	32	-
**3**	Mn(^{Cl}pdp) (**3**)	47	8	32	-
**4**	Mn(^{CO_2Et}pdp) (**4**)	75	14	56	-
**5**	Mn(^{CF_3}pdp) (**5**)	36	6	26	-
**6**	Mn($^{p\text{-}TIPS}$pdp) (**6**)	51	24	25	-
**7**	Mn(^{DMM}pdp) (**7**)	58	22	22	12
**8**	Mn(^{Me_2N}pdp) (**8**)	63	10	5	40

[a]Conversion and product yields were determined by GC and were averaged over at least two independent experiments.

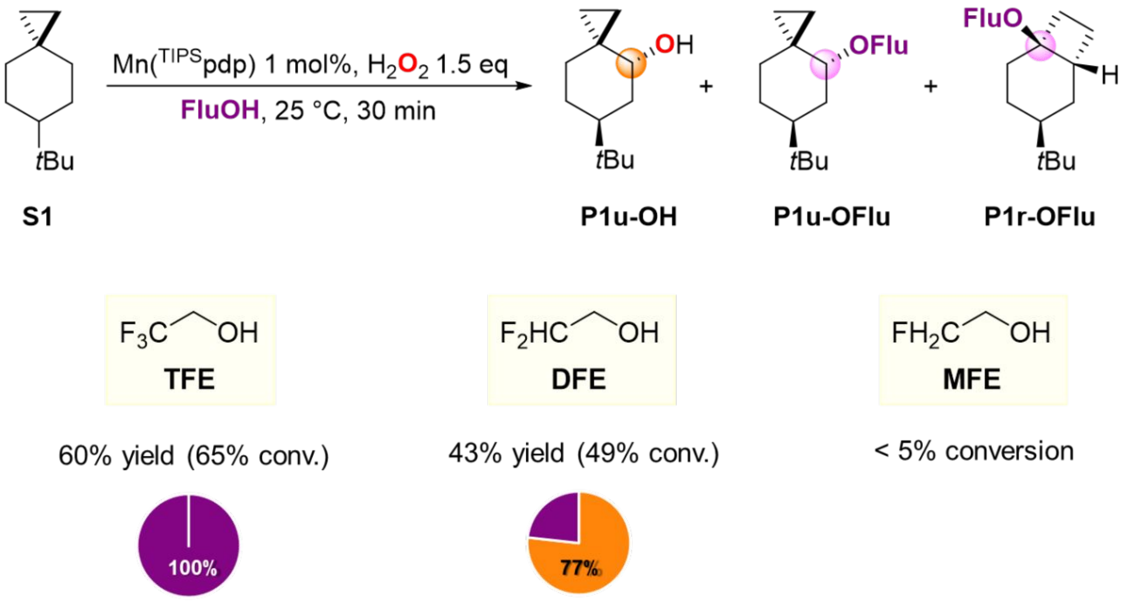

Scheme S6. Oxidation of **S1** with different fluorinated alcohol solvents.

4.3. Oxidation of S2-S5

Table S7. Oxidation of 6-ethylspiro[2.5]octane (**S2**) *via* carboxylate rebound.[a]

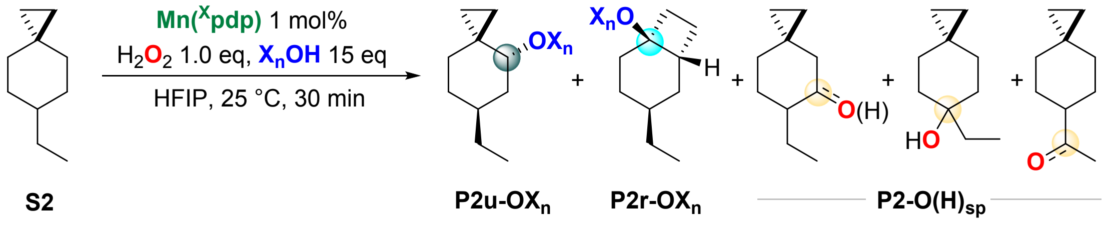

Entry	Mn(Xpdp)	X_nOH	% conv	% product yields		
				P2u-OXn	P2r-OXn	P2-O(H)$_{sp}$
1	1[b]	X_7OH[c]	66	35	13	8
2	4[d]	X_8OH[e]	66	5	38	6

[a]Conversion and product yields were determined by GC and were averaged over at least two independent experiments. [b]**1** = Mn(^{TIPS}pdp). [c]**X_7OH** = 2,2-dimethylbutanoic acid. [d]**4** = Mn(^{CO_2Et}pdp). [e]**X_8OH** = chloroacetic acid.

Table S8. Oxidation of 6-ethylspiro[2.5]octane (**S2**) *via* TFE rebound.[a]

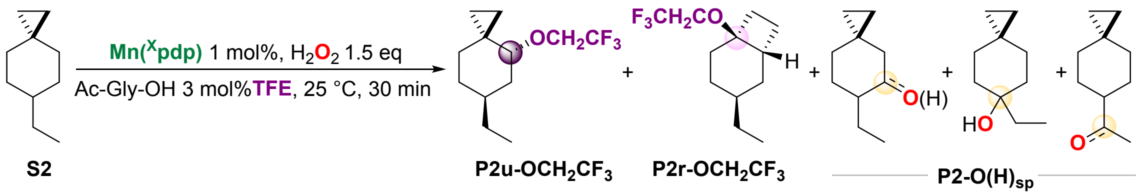

Entry	Mn(Xpdp)	% conv	% product yields		
			P2u-OXn	P2r-OXn	P2-O(H)$_{sp}$
1	1[b]	89	33	38	10
2	4[d]	76	12	50	8

[a]Conversion and product yields were determined by GC and were averaged over at least two independent experiments. [b]**1** = Mn(^{TIPS}pdp). [d]**4** = Mn(^{CO_2Et}pdp).

Table S9. Oxidation of 1,1-diethylcyclopropane (**S3**) *via* carboxylate rebound.[a]

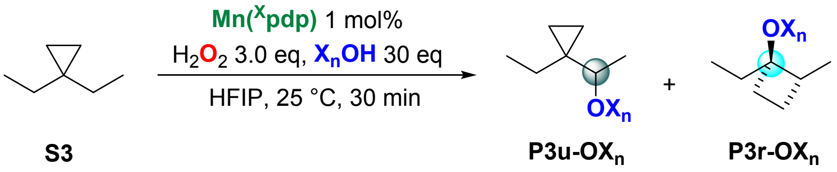

Entry	Mn(Xpdp)	X_nOH	% conv	% product yields	
				P3u-OXn	P3r-OXn
1	**1**[b]	X_7OH[c]	70	35	15
2[d]	**4**[e]	X_8OH[f]	66	-	49

[a]Conversion and product yields were determined by GC and were averaged over at least two independent experiments. [b]**1** = Mn(^{TIPS}pdp). [c]X_7OH = 2,2-dimethylbutanoic acid. [d]2.0 equiv. of H_2O_2 were used. [e]**4** = Mn(^{CO_2Et}pdp). [f]X_8OH = chloroacetic acid.

Table S10. Oxidation of 1,1-diethylcyclopropane (**S3**) *via* TFE rebound.[a]

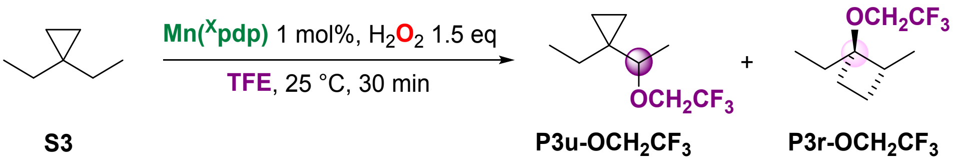

Entry	Mn(Xpdp)	% conv	% product yields	
			P3u-OCH2CF3	P3r-OCH2CF3
1	**1**[b]	82	10	49
2[d]	**4**[e]	51	-	38

[a]Conversion and product yields were determined by GC and were averaged over at least two independent experiments. [b]**1** = Mn(^{TIPS}pdp). [e]**4** = Mn(^{CO_2Et}pdp).

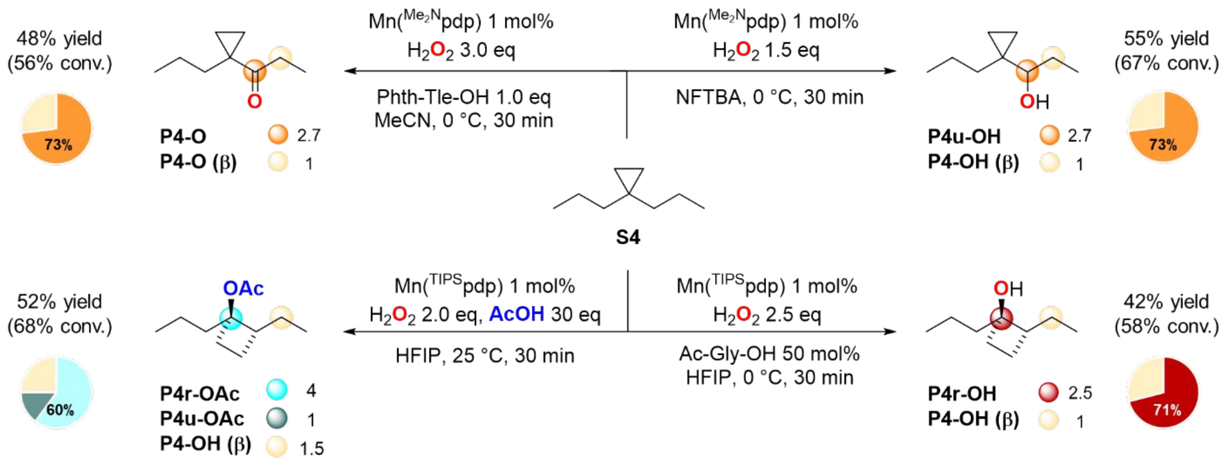

Scheme S7. Oxidation of 1,1-dipropylcyclopropane (**S4**).

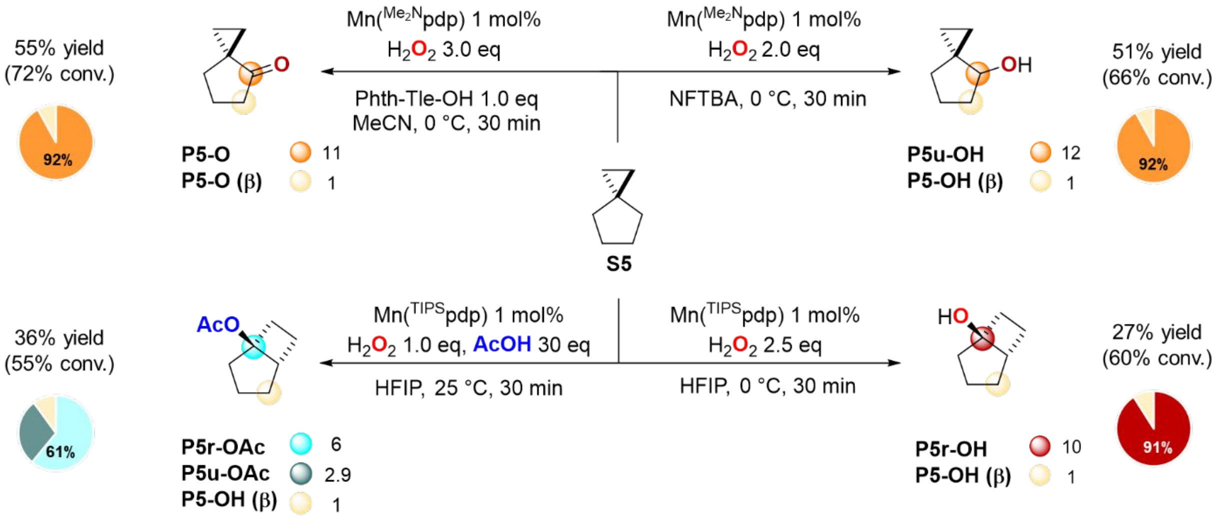

Scheme S8. Oxidation of spiro[2.4]heptane (**S5**).

5. Isolation and characterization of the oxidation products

Trans-6-*tert*-butylspiro[2.5]octan-4-ol (**P1u-OH**), *cis*-4-(*tert*-butyl)-bicyclo[4.2.0]octan-1-ol (**P1u-OH**) and 6-*tert*-butylspiro[2.5]octan-2-one (**P1-O**), *trans*-6-*tert*-butylspiro[2.5]octan-4-yl acetate (**P1u-OX$_1$**), *trans*-6-*tert*-butylspiro[2.5]octan-4-yl propionate (**P1u-OX$_2$**), *trans*-6-*tert*-butylspiro[2.5]octan-4-yl cyclopropanecarboxylate (**P1u-OX$_3$**), *trans*-6-*tert*-butylspiro[2.5]octan-4-yl pivalate (**P1u-OX$_5$**), *trans*-6-*tert*-butylspiro[2.5]octan-4-yl 2,2-dimethylbutanoate (**P1u-OX$_7$**), *cis*-4-*tert*-butylbicyclo[4.2.0]octan-1-yl acetate (**P1r-OX$_1$**), c*is*-4-*tert*-butylbicyclo[4.2.0]octan-1-yl cyclopropanecarboxylate (**P1r-OX$_3$**), *cis*-4-*tert*-butylbicyclo[4.2.0]octan-1-yl pivalate (**P1r-OX$_5$**) were prepared according to reported procedures.[1] The other ester products derived by oxidation of **S1** were prepared by esterification of the alcohols **P1u-OH** and **P1r-OH**. The ether products (**P1u-OCH$_2$CF$_3$** and **P1r-OCH$_2$CF$_3$**) were isolated and characterized after scale-up oxidation of **S1**.
1-(1-ethylcyclopropyl)ethan-1-ol (**P3u-OH**), 1-ethyl-2-methylcyclobutan-1-ol (**P3r-OH**), 1-ethyl-2-methylcyclobutyl acetate (**P3r-OAc**) were isolated and characterized after scale-up oxidation of **S2**. The ester and ether products derived from oxidation of **S2** and **S3** via carboxylate- and TFE-rebound (see Scheme 8 in the manuscript) were prepared by scale-up oxidation of the respective substrates. The other oxidation products were characterized by GC-MS.

General procedure for acylation of alcohols P1u-OH and P1r-OH.[1] 0.15 mmol of the alcohols (1.0 equiv), 1.5 equiv of triethylamine, 5 mol% of 4-dimethylaminopyridine and 2.5 ml of CH_2Cl_2 were introduced in a 10 mL vial and kept under nitrogen. 1.5 equiv of the corresponding acyl chloride were then added dropwise to the reaction mixture at 0 °C under magnetic stirring. After the addition, the solution was brought at room temperature and kept under stirring for 4 hours. The reaction mixture was then quenched with 2.5 mL of a 2 M HCl solution, and the organic layer was separated. Thus 2.5 mL of saturated $NaHCO_3$ aqueous solution were then added and the solution was kept under stirring for 30 min to remove the excess of the acyl chloride. At this point, the organic phase was extracted with 2 x 2.5 mL of saturated $NaHCO_3$ aqueous solution, 2 x 2.5 mL of brine and then dried over $MgSO_4$. After filtration, the solution was concentrated under vacuum and the crude was purified by flash chromatography on silica gel. The characterization of the products was accomplished by ^{1}H-NMR, ^{13}C-NMR and HMRS.

General procedure for scale-up oxidation of S1-S5. A MeCN, TFE, HFIP or NFTBA (volume range 2.5-4 mL) solution of substrate (1.0 equiv, 0.125 M), Mn catalyst (1 mol%) was prepared in a 25 mL round bottom flask equipped with a magnetic stirring bar. When used, the carboxylic acid (15 equiv) or the amino acid (range 0.03 – 1.0 equiv) were added and the resulting mixture was cooled at 0 °C (ice bath) or 25 °C. Then ~1.0 M H_2O_2 solution in the pertinent solvent (range 1.0 – 3.5 equiv) was directly added by syringe pump over 30 minutes. Then the solvent was evaporated at reduced pressure and the crude was rinsed with 5 mL of CH_2Cl_2. The resulting organic solution was extracted two times with 2 x 5 mL saturated $NaHCO_3$ aqueous solution and one time with 5 mL of brine. The reunited organic phases were dried with $MgSO_4$, filtered and then evaporated at reduced pressure. The resulting residue was purified by flash chromatography on silica gel and the spectroscopic data of the pure products were compared with those reported in literature. The characterization of the unknown products was performed by ^{1}H-NMR, ^{13}C-NMR and HRMS.

5.1 Acylation of alcohols P1u-OH and P1r-OH

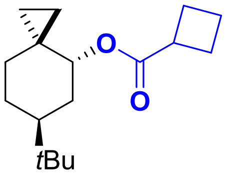

***Trans*-6-(*tert*-butyl)spiro[2.5]octan-4-yl cyclobutanecarboxylate (P1u-OX$_4$)**. Purification by flash chromatography (pentane-diethyl ether 50:1) afforded 16.4 mg of *trans*-6-(*tert*-butyl)spiro[2.5]octan-4-yl cyclobutanecarboxylate (**P1u-OX$_4$**, 0.06 mmol, 40% yield) as colorless liquid. ^{1}H-NMR (400 MHz, $CDCl_3$) δ, ppm: 4.30 (p, J = 1.6 Hz, 1H), 3.23 – 3.13 (m, 1H), 2.35 – 2.22 (m, 4H), 2.09 (tdd, J = 13.3, 3.8, 1.6 Hz, 1H), 2.01 – 1.90 (m, 3H), 1.81 – 1.73 (m, 1H), 1.43 – 1.36 (m, 2H), 1.20 (td, J = 12.3, 3.8 Hz, 1H), 0.87 (s, 9H), 0.80 (dtd, J = 13.3, 3.5, 1.3 Hz, 1H), 0.57 – 0.52 (m, 1H), 0.40 – 0.30 (m, 3H). ^{13}C{^{1}H}-NMR (400 MHz, $CDCl_3$) δ, ppm: 175.20, 77.75, 41.61, 38.68, 32.04, 31.44, 30.97, 27.48, 25.80, 25.50, 25.21, 21.42, 18.52, 11.71, 10.91. HRMS (ESI-MS) *m/z* calculated for $C_{17}H_{28}O_2$ $[M+Na]^+$ 287.1982, found 287.1979.

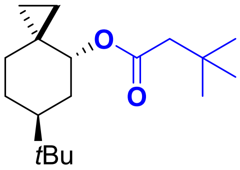

***Trans*-6-(*tert*-butyl)spiro[2.5]octan-4-yl 3,3-dimethylbutanoate (P1u-OX$_6$)**. Purification by flash chromatography (pentane-diethyl ether 50:1) afforded 19.8 mg of *trans*-6-(*tert*-butyl)spiro[2.5]octan-4-yl 3,3-dimethylbutanoate (**P1u-OX$_6$**, 0.07 mmol, 47% yield) as colorless liquid. ^{1}H-NMR (400 MHz, $CDCl_3$) δ, ppm: δ 4.35 (s, 1H), 2.24 (s, 2H), 2.13 – 2.03 (m, 1H), 1.95 – 1.88 (m, 1H), 1.81 – 1.73 (m, 1H), 1.48 – 1.34 (m, 2H), 1.22 – 1.16 (m, 1H), 1.08 (s, 9H), 0.87 (s, 9H), 0.79 (dtd, J = 13.3, 3.7, 1.3 Hz, 1H), 0.57 – 0.53 (m, 1H), 0.41 – 0.36 (m, 1H), 0.34 – 0.31 (m, 2H). ^{13}C{^{1}H}-NMR (400 MHz, $CDCl_3$) δ, ppm: 172.09, 77.73, 48.70, 41.59, 32.12, 31.49, 31.06, 30.76, 29.77, 27.45, 25.78, 21.52, 11.92, 11.06. HRMS (ESI-MS) *m/z* calculated for $C_{18}H_{32}O_2$ $[M+Na]^+$ 303.2295, found 303.2299.

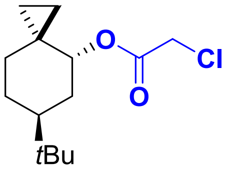

***Trans*-6-(*tert*-butyl)spiro[2.5]octan-4-yl 2-chloroacetate (P1u-OX$_8$)**. Purification by flash chromatography (pentane-diethyl ether 50:1) afforded 22.4 mg of *trans*-6-(*tert*-butyl)spiro[2.5]octan-4-yl 2-chloroacetate (**P1u-OX$_8$**, 0.09 mmol, 60% yield) as colorless liquid. ^{1}H-NMR (400 MHz, $CDCl_3$) δ, ppm: 4.42 (dt, J = 3.1, 1.7 Hz, 1H), 4.11 (s, 2H), 2.08 (tdd, J = 13.3, 3.7, 1.5 Hz, 1H), 2.02 – 1.92 (m, 1H), 1.77 (t, J = 3.2 Hz, 1H), 1.50 – 1.37 (m, 2H), 1.26 – 1.14 (m, 1H), 0.88 (s, 9H), 0.81 (dtd, J = 13.4, 3.5, 1.4 Hz, 1H), 0.64 – 0.55 (m, 1H), 0.43 – 0.32 (m, 3H). ^{13}C{^{1}H}-NMR (400 MHz, $CDCl_3$) δ, ppm: 167.02, 80.82, 41.44, 32.07, 31.18, 30.89, 27.43, 25.66, 21.39, 11.80, 11.16. HRMS (ESI-MS) *m/z* calculated for $C_{14}H_{23}ClO_2$ $[M+Na]^+$ 281.1279, found 281.1276.

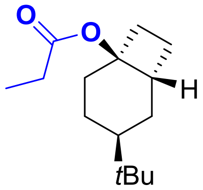

***Cis*-4-*tert*-butylbicyclo[4.2.0]octan-1-yl propionate (P1r-OX_2)**. Purification by flash chromatography (pentane-diethyl ether 50:1) afforded 13.2 mg of *cis*-4-*tert*-butylbicyclo[4.2.0]octan-1-yl propionate (**P1r-OX_2**, 0.055 mmol, 37% yield) as colorless liquid. ^{1}H NMR (400 MHz, $CDCl_3$) δ, ppm: 2.58 (q, J = 9.8 Hz, 1H), 2.37 – 2.25 (m, 3H), 2.23 – 2.15 (m, 1H), 2.03 (q, J = 10.5, 10.0 Hz, 1H), 1.78 – 1.65 (m, 2H), 1.57 – 1.44 (m, 3H), 1.33 (td, J = 13.1, 6.2 Hz, 1H), 1.23 – 1.17 (m, 1H), 1.13 (t, J = 7.6 Hz, 3H), 1.02 – 0.93 (m, 1H), 0.87 (s, 9H). ^{13}C{^{1}H}-NMR (400 MHz, $CDCl_3$) δ, ppm: 173.48, 78.05, 43.37, 41.35, 34.07, 32.80, 32.33, 28.20, 27.43, 25.41, 22.33, 17.97, 9.15. HRMS (ESI-MS) *m/z* calculated for $C_{15}H_{26}O_2$ $[M+Na]^+$ 261.1825, found 261.1825.

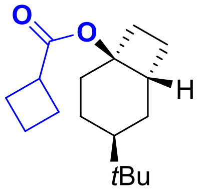

***Cis*-4-*tert*-butylbicyclo[4.2.0]octan-1-yl cyclobutanecarboxylate (P1r-OX_4)**. Purification by flash chromatography (pentane-diethyl ether 50:1) afforded 15.4 mg of *cis*-4-*tert*-butylbicyclo[4.2.0]octan-1-yl cyclobutanecarboxylate (**P1r-OX_4**, 0.058 mmol, 39% yield) as colorless liquid. ^{1}H NMR (400 MHz, $CDCl_3$) δ, ppm: 3.08 (pd, J = 8.6, 1.1 Hz, 1H), 2.62 – 2.54 (m, 1H), 2.36 – 2.29 (m, 1H), 2.27 – 2.20 (m, 2H), 2.19 – 2.16 (m, 1H), 2.05 – 1.88 (m, 3H), 1.77 – 1.67 (m, 2H), 1.58 – 1.44 (m, 4H), 1.39 – 1.27 (m, 2H), 1.24 – 1.13 (m, 1H), 1.00 – 0.90 (m, 1H), 0.87 (s, 9H). ^{13}C{^{1}H}-NMR (400 MHz, $CDCl_3$) δ, ppm: 174.51, 77.93, 43.39, 41.31, 38.58, 34.09, 32.83, 32.33, 27.43, 25.43, 25.09, 25.05, 22.35, 18.31, 17.96. HRMS (ESI-MS) *m/z* calculated for $C_{17}H_{28}O_2$ $[M+Na]^+$ 287.1982, found 287.1984.

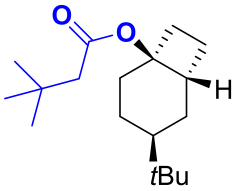

***Cis*-4-*tert*-butylbicyclo[4.2.0]octan-1-yl 3,3-dimethylbutanoate (P1r-OX_6)**. Purification by flash chromatography (pentane-diethyl ether 50:1) afforded 18.2 mg of *cis*-4-*tert*-butylbicyclo[4.2.0]octan-1-yl 3,3-dimethylbutanoate (**P1r-OX_6**, 0.065 mmol, 43% yield) as colorless liquid. ^{1}H NMR (400 MHz, $CDCl_3$) δ, ppm: 2.63 – 2.54 (m, 1H), 2.40 – 2.32 (m, 1H), 2.25 – 2.17 (m, 1H), 2.14 (s, 2H), 2.04 (q, J = 10.2 Hz, 1H), 1.77 – 1.65 (m, 2H), 1.57 – 1.44 (m, 3H), 1.34 (dd, J = 12.8, 6.1 Hz, 1H), 1.22 – 1.14 (m, 1H), 1.05 (s, 9H), 1.00 – 0.92 (m, 1H), 0.87 (s, 9H). ^{13}C{^{1}H}-NMR (400 MHz, $CDCl_3$)

δ, ppm: 171.42, 77.98 48.53, 43.35, 41.48, 34.17, 32.81, 32.32, 30.89, 29.70, 27.41, 25.42, 22.30, 18.06. HRMS (ESI-MS) *m/z* calculated for $C_{18}H_{32}O_2$ $[M+Na]^+$ 303.2295, found 303.2302.

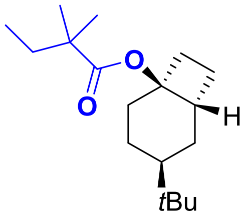

***Cis*-4-*tert*-butylbicyclo[4.2.0]octan-1-yl 2,2-dimethylbutanoate (P1r-OX$_7$)**. Purification by flash chromatography (pentane-diethyl ether 50:1) afforded 14.6 mg of *cis*-4-*tert*-butylbicyclo[4.2.0]octan-1-yl 2,2-dimethylbutanoate (**P1r-OX$_7$**, 0.052 mmol, 35% yield) as colorless liquid. ^{1}H NMR (400 MHz, $CDCl_3$) δ, ppm: 2.58 (q, J = 9.7 Hz, 1H), 2.33 – 2.27 (m, 1H), 2.18 (ddd, J = 11.1, 6.9, 2.6 Hz, 1H), 2.08 – 1.95 (m, 2H), 1.77 – 1.68 (m, 2H), 1.57 – 1.46 (m, 5H), 1.33 – 1.30 (m, 1H), 1.21 – 1.18 (m, 1H), 1.14 (d, J = 1.3 Hz, 6H), 0.87 (d, J = 2.0 Hz, 12H). $^{13}C\{^1H\}$-NMR (400 MHz, $CDCl_3$) δ, ppm: 176.97, 77.77, 43.43, 42.53, 41.26, 34.10, 33.32, 32.75, 32.30, 27.42, 25.48, 24.62, 22.35, 18.02, 9.26. HRMS (ESI-MS) *m/z* calculated for $C_{18}H_{32}O_2$ $[M+Na]^+$ 303.2295, found 303.22294.

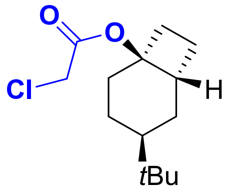

***Cis*-4-*tert*-butylbicyclo[4.2.0]octan-1-yl 2-chloroacetate (P1r-OX$_8$)**. Purification by flash chromatography (pentane-diethyl ether 50:1) afforded 20.4 mg of *cis*-4-*tert*-butylbicyclo[4.2.0]octan-1-yl 2-chloroacetate (**P1r-OX$_8$**, 0.079 mmol, 52% yield) as colorless liquid. ^{1}H NMR (400 MHz, $CDCl_3$) δ, ppm: 4.01 (s, 2H), 2.70 – 2.61 (m, 1H), 2.34 (dq, J = 15.4, 2.9, 2.0 Hz, 1H), 2.25 – 2.17 (m, 1H), 2.13 – 2.03 (m, 1H), 1.82 – 1.69 (m, 2H), 1.54 – 1.47 (m, 2H), 1.39 – 1.29 (m, 2H), 1.24 – 1.15 (m, 1H), 1.00 – 0.93 (m, 1H), 0.88 (s, 9H). $^{13}C\{^1H\}$-NMR (400 MHz, $CDCl_3$) δ, ppm: 165.94, 80.37, 43.24, 41.53, 41.28, 33.79, 32.71, 32.33, 27.41, 25.26, 22.21, 17.72. HRMS (ESI-MS) *m/z* calculated for $C_{14}H_{23}ClO_2$ $[M+Na]^+$ 281.1279, found 281.1286.

5.2 Scale-up oxidation of S1-S3

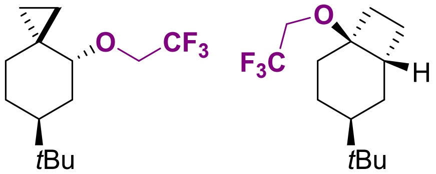

***Trans*-6-(*tert*-butyl)-4-(2,2,2-trifluoroethoxy)spiro[2.5]octane (P1u-OCH_2CF_3) and *cis*-4-(*tert*-butyl)-1-(2,2,2-trifluoroethoxy)bicyclo[4.2.0]octane (P1r-OCH_2CF_3).** Prepared following the general procedure on 1.2 mmol of **S1**. General conditions: 9.8 mg of Mn(^{CO_2Et}pdp) (0.012 mmol, 1 mol%), 200 mg of **S1** (1.2 mmol, 1.0 equiv), 2.94 ml of 0.613 M H_2O_2 solution in TFE (1.8 mmol, 1.5 equiv), 12 mL of TFE at 25 °C for 30 min. Purification by flash chromatography over silica gel in pure pentane afforded 52.3 mg of *trans*-6-(*tert*-butyl)-4-(2,2,2-trifluoroethoxy)spiro[2.5]octane (**P1u-OCH_2CF_3**, 0.16 mmol, 13% yield**)** and 163.6 mg of *cis*-4-(*tert*-butyl)-1-(2,2,2-trifluoroethoxy)bicyclo[4.2.0]octane (**P1r-OCH_2CF_3**, 0.62 mmol, 52% yield) as colorless liquids.

P1u-OCH_2CF_3: ^{1}H-NMR (400 MHz, $CDCl_3$) δ, ppm: 3.97 – 3.76 (m, 2H), 2.87 (s, 1H), 2.06 (tdd, J = 13.2, 4.7, 1.9 Hz, 1H), 1.98 (dq, J = 13.6, 3.1 Hz, 1H), 1.74 (dq, J = 12.4, 3.1 Hz, 1H), 1.56 – 1.48 (m, 1H), 1.33 – 1.30 (m, 1H), 1.21 – 1.11 (m, 1H), 0.89 (s, 9H), 0.74 – 0.68 (m, 1H), 0.58 (dt, J = 9.7, 5.1 Hz, 1H), 0.49 (dt, J = 8.9, 5.1 Hz, 1H), 0.26 (ddd, J = 8.9, 5.6, 4.4 Hz, 1H), 0.16 (dddd, J = 9.9, 5.9, 4.4, 1.8 Hz, 1H). $^{13}C\{^1H\}$-NMR (400 MHz, $CDCl_3$) δ, ppm: 124.24, 84.31, 65.48, 40.52, 32.06, 30.69, 30.54, 27.46, 25.79, 20.91, 11.92, 9.52. ^{19}F-NMR (400 MHz, $CDCl_3$) δ, ppm: -75.27. HRMS (ESI-MS) *m/z* calculated for $C_{14}H_{23}F_3O$ $[M+Na]^+$ 287.1593, found 287.1597.

P1r-OCH_2CF_3: ^{1}H-NMR (400 MHz, $CDCl_3$) δ, ppm: 3.81 – 3.64 (m, 2H), 2.64 – 2.55 (m, 1H), 2.07 – 1.99 (m, 1H), 1.86 (t, J = 9.7 Hz, 1H), 1.70 – 1.63 (m, 2H), 1.58 – 1.52 (m, 2H), 1.51 – 1.37 (m, 2H), 1.29 (q, J = 7.5, 7.0 Hz, 1H), 1.18 (tdd, J = 12.6, 3.7, 1.9 Hz, 1H), 1.08 – 0.96 (m, 1H), 0.87 (s, 9H). $^{13}C\{^1H\}$-NMR (400 MHz, $CDCl_3$) δ, ppm: 124.19, 77.37, 61.11, 43.50, 40.56, 32.38, 32.33, 30.19, 27.43, 25.31, 22.10, 15.78. ^{19}F-NMR (400 MHz, $CDCl_3$) δ, ppm: -75.47. HRMS (ESI-MS) *m/z* calculated for $C_{14}H_{23}F_3O$ $[M+Na]^+$ 287.1593, found 287.1596.

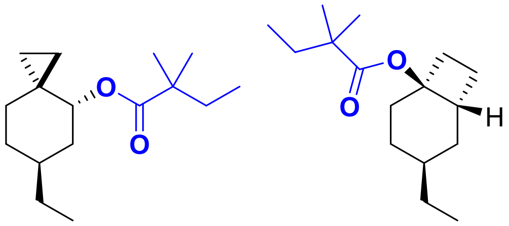

***Trans*-6-ethylspiro[2.5]octan-4-yl 2,2-dimethylbutanoate (P2u-OX_7) and *cis*-4-ethylbicyclo[4.2.0]octan-1-yl 2,2-dimethylbutanoate (P2r-OX_7).** Prepared following the general procedure on 1.08 mmol of **S2**. General conditions: 10.7 mg of Mn(^{TIPS}pdp) (0.0108 mmol, 1 mol%), 150 mg of **S2** (1.08 mmol, 1.0 equiv), 2.0 ml of 2,2-dimethylbutanoic acid (16.2 mmol, 15 equiv), 1.46 ml of 0.741 M H_2O_2 solution in HFIP (1.08 mmol, 1.0 equiv), 11 mL of HFIP at 25 °C for 30 min. Purification by flash chromatography over silica gel (pentane-diethyl ether 50:1) afforded 72.3 mg of *trans*-6-ethylspiro[2.5]octan-4-yl 2,2-dimethylbutanoate (**P2u-OX_7**, 0.286 mmol, 26% yield) as colorless liquid. 29.5 (0.286 mmol, 26% yield) mg of an inseparable mixture of **P2u-OX_7** and **P2r-OX_7** was also collected. **P2r-OX_7** was characterized by GC-MS analysis.

P2u-OX$_7$: ^{1}H-NMR (400 MHz, $CDCl_3$) δ, ppm: 4.28 – 4.24 (m, 1H), 2.09 (tdd, J = 13.3, 3.8, 1.7 Hz, 1H), 1.87 (dtd, J = 13.8, 3.4, 2.0 Hz, 1H), 1.76 (dq, J = 12.5, 3.2 Hz, 1H), 1.62 (dd, J = 15.0, 1.3 Hz, 2H), 1.29 – 1.25 (m, 2H), 1.19 (s, 6H), 1.14 (d, J = 1.9 Hz, 1H), 1.12 – 1.05 (m, 1H), 0.89 (td, J = 7.5, 6.4 Hz, 7H), 0.80 – 0.74 (m, 1H), 0.56 – 0.50 (m, 1H), 0.41 – 0.27 (m, 3H). $^{13}C\{^1H\}$-NMR (400 MHz, $CDCl_3$) δ, ppm: 177.45, 77.11, 42.90, 36.06, 33.55, 33.51, 31.14, 31.05, 29.50, 24.84, 24.76, 21.61, 11.46, 11.41, 10.90, 9.29. HRMS (ESI-MS) *m/z* calculated for $C_{16}H_{28}O_2$ $[M+Na]^+$ 275.1982, found 275.1982. GC-MS (CI-NH_3): *m/z* for $C_{16}H_{28}O_2$ $[M+H]^+$ 253.1, $[M+NH_4]^+$ 270.1.
P2r-OX$_7$: GC-MS (CI-NH_3): *m/z* for $C_{16}H_{28}O_2$ $[M+H]^+$ 253.2, $[M+NH_4]^+$ 270.1.

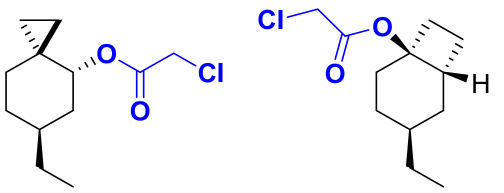

***Trans*-6-ethylspiro[2.5]octan-4-yl 2-chloroacetate (P2u-OX$_8$) and *cis*-4-ethylbicyclo[4.2.0]octan-1-yl 2-chloroacetate (P2r-OX$_8$).** Prepared following the general procedure on 1.08 mmol of **S2**. General conditions: 8.8 mg of Mn(^{CO_2Et}pdp) (0.0108 mmol, 1 mol%), 150 mg of **S2** (1.08 mmol, 1.0 equiv), 1.5 g of chloroacetic acid (16.2 mmol, 15 equiv), 1.55 ml of 0.695 M H_2O_2 solution in HFIP (1.08 mmol, 1.0 equiv), 11 mL of HFIP at 25 °C for 30 min. Purification by flash chromatography over silica gel (pentane-diethyl ether 50:1) afforded 77.4 mg (0.335 mmol, 34% yield) of an inseparable mixture of *trans*-6-ethylspiro[2.5]octan-4-yl 2-chloroacetate (**P2u-OX$_8$**) and *cis*-4-ethylbicyclo[4.2.0]octan-1-yl 2-chloroacetate (**P2r-OX$_8$**) (ratio **P2r-OX$_8$**: **P2u-OX$_8$** = 4.6). **P2u-OX$_8$** and **P2r-OX$_8$** were characterized by ^{1}H-NMR, selective TOCSY (see section 6.4 for NMR spectra) and GC-MS analysis.
P3u-OX$_8$: GC-MS (CI-NH_3): *m/z* for $C_{12}H_{19}ClO_2$ $[M+H]^+$ 231.1, $[M+NH_4]^+$ 248.1.
P3r-OX$_8$: GC-MS (CI-NH_3): *m/z* for $C_{12}H_{19}ClO_2$ $[M+H]^+$ 231.0, $[M+NH_4]^+$ 248.1.

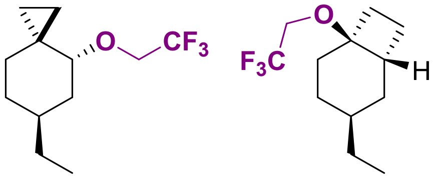

***Trans*-6-ethyl-4-(2,2,2-trifluoroethoxy)spiro[2.5]octane (P2u-OCH$_2$CF$_3$) and *cis*-4-ethyl-1-(2,2,2-trifluoroethoxy)bicyclo[4.2.0]octane (P2r-OCH$_2$CF$_3$).** Prepared following the general procedure on 1.45 mmol of **S2**. General conditions: 9.8 mg of Mn(^{CO_2Et}pdp) (0.012 mmol, 1 mol%), 200 mg of **S2** (1.45 mmol, 1.0 equiv), 3.10 ml of 0.934 M H_2O_2 solution in TFE (2.9 mmol, 2.0 equiv), 15 mL of TFE at 25 °C for 30 min. Purification by flash chromatography over silica gel in pure pentane afforded 115.2 mg of *cis*-4-ethyl-1-(2,2,2-trifluoroethoxy)bicyclo[4.2.0]octane (**P2r-OCH$_2$CF$_3$**, 0.49 mmol, 34% yield) as colorless liquid and 69.8 mg (0.30 mmol, 21% yield) of an inseparable mixture of **P2u-OCH$_2$CF$_3$** and **P2r-OCH$_2$CF$_3$**. **P2u-OCH$_2$CF$_3$** was characterized by GC-MS analysis of the mixture.
P2u-OCH$_2$CF$_3$: GC-MS (CI-NH_3): *m/z* for $C_{12}H_{19}F_3O$ $[M+H]^+$ 237.0, $[M+NH_4]^+$ 254.1.

P2r-OCH$_2$CF$_3$: ^{1}H-NMR (400 MHz, CDCl$_3$) δ, ppm: 3.83 – 3.63 (m, 2H), 2.60 – 2.50 (m, 1H), 2.00 (dtd, J = 16.0, 4.1, 2.0 Hz, 1H), 1.88 (q, J = 9.8 Hz, 1H), 1.68 (td, J = 8.9, 1.5 Hz, 2H), 1.59 – 1.49 (m, 3H), 1.40 (qd, J = 10.4, 7.7 Hz, 1H), 1.31 – 1.21 (m, 3H), 1.13 (ddd, J = 14.0, 11.7, 6.2 Hz, 1H), 1.04 – 0.94 (m, 1H), 0.91 (t, J = 7.3 Hz, 3H). ^{13}C{^{1}H}-NMR (400 MHz, CDCl$_3$) δ, ppm: 124.22, 77.62, 61.13, 40.26, 34.91, 31.70, 30.95, 30.57, 29.77, 27.20, 16.02, 11.41. ^{19}F-NMR (400 MHz, CDCl$_3$) δ, ppm: -75.51. HRMS (ESI-MS) *m/z* calculated for $C_{12}H_{19}F_3O$ $[M+Na]^+$ 259.1280, found 259.1279.

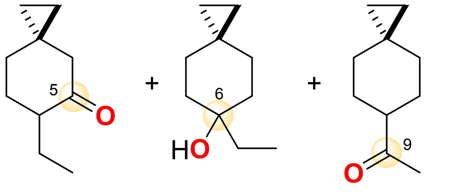

P2-O(5) **P2-OH (6)** **P2-O (9)**

The reaction mixture derived from the oxidation of **S2** in the conditions reported in Table S6, entry 1 was oxidized by Jones reagent according to a reported procedure.[8] GC-MS analysis of the crude gave the following spectrometric data:
P2-O(5): GC-MS (CI-NH$_3$): *m/z* for $C_{10}H_{16}O$ $[M+H]^+$ 153.0, $[M+NH_4]^+$ 170.0.
P2-OH (6): *m/z* for $C_{10}H_{18}O$ $[M-H_2O]^+$ 154.0, $[M-H_2O+NH_4]^+$ 171.9.
P2-O (9): *m/z* for $C_{10}H_{16}O$ $[M+H]^+$ 153.0, $[M+NH_4]^+$ 170.0.

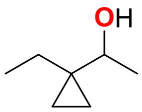

1-(1-ethylcyclopropyl)ethan-1-ol (P3u-OH). Prepared following the general procedure on 1.53 mmol of **S3**. General conditions: 12 mg of Mn(^{Me_2N}pdp) (0.0153 mmol, 1 mol%), 150 mg of **S3** (1.53 mmol, 1.0 equiv), 3.28 mL of 0.7 M H_2O_2 solution in NFTBA (2.3 mmol, 1.5 equiv), 20 mL of NFTBA at 0 °C for 30 min. Purification by flash chromatography over silica gel (pentane-diethyl ether 15:1) afforded 43.2 mg of 1-(1-ethylcyclopropyl)ethan-1-ol **(P3u-OH**, 0.378 mmol, 25% yield) as colorless liquid. The obtained poor yield as compared to that observed with GC analysis (50%) is due to the high volatility of the product. ^{1}H-NMR (400 MHz, CDCl$_3$) δ, ppm: 3.43 (qd, J = 6.3, 2.5 Hz, 1H), 1.66 (dd, J = 14.4, 7.3 Hz, 1H), 1.36 (dd, J = 14.6, 7.2 Hz, 1H), 1.21 (d, J = 6.4 Hz, 3H), 0.91 (t, J = 7.5 Hz, 4H), 0.45 – 0.27 (m, 4H). ^{13}C{^{1}H}-NMR (400 MHz, CDCl$_3$) δ, ppm: 73.05, 26.10, 24.80, 19.59, 10.93, 9.41, 8.52. HRMS (ESI-MS) *m/z* calculated for $C_7H_{14}O$ $[M+Na]^+$ 137.0937, found 137.0940.

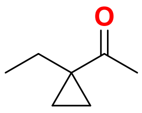

1-(1-ethylcyclopropyl)ethan-1-ol (P3-O). Prepared by Jones oxidation of **P3u-OH** according to a reported procedure.[8] The isolation of **P3-O** wasn't accomplished due to the high volatility of the product. GC-MS (CI-NH$_3$): *m/z* for $C_7H_{12}O$ $[M+H]^+$ 113.0, $[M+NH_4]^+$ 130.1.

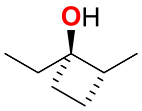

1-ethyl-2-methylcyclobutan-1-ol (P3r-OH). Prepared following the general procedure on 1.53 mmol of **S3**. General conditions: 15 mg of Mn(^{TIPS}pdp) (0.0153 mmol, 1 mol%), 150 mg of **S3** (1.53 mmol, 1.0 equiv), 90 mg of Ac-Gly-OH (0.76 mmol, 50 mol%), 2.44 ml of 0.94 M H_2O_2 solution in HFIP (2.3 mmol, 1.5 equiv), 15 mL of HFIP at 25 °C for 30 min. Purification by flash chromatography over silica gel (pentane-diethyl ether 15:1) afforded 65 mg of 1-ethyl-2-methylcyclobutan-1-ol (**P3r-OH**, 0.57 mmol, 37% yield) as colorless liquid. ^{1}H-NMR (400 MHz, $CDCl_3$) δ, ppm: 2.33 (ddt, J = 16.1, 9.2, 7.0 Hz, 1H), 2.05 – 1.98 (m, 1H), 1.90 – 1.81 (m, 1H), 1.81 – 1.71 (m, 1H), 1.61 (s, 1H), 1.58 – 1.51 (m, 2H), 1.13 – 1.03 (m, 1H), 1.00 – 0.93 (m, 6H). ^{13}C{^{1}H}-NMR (400 MHz, $CDCl_3$) δ, ppm: 76.98, 43.52, 32.78, 26.12, 20.31, 13.96, 6.97. HRMS (ESI-MS) *m/z* calculated for $C_7H_{14}O$ $[M+Na]^+$ 137.0937, found 137.0942.

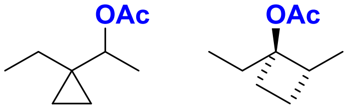

1-(1-ethylcyclopropyl)ethyl acetate (P3u-OAc) and 1-ethyl-2-methylcyclobutyl acetate (P3r-OAc). Prepared following the general procedure on 1.53 mmol of **S3**. General conditions: 15.1 mg of Mn(^{TIPS}pdp) (0.0153 mmol, 1 mol%), 150 mg of **S1** (1.53 mmol, 1.0 equiv), 2.6 ml of AcOH (4.59 mmol, 30 equiv), 1.83 ml of 0.838 M H_2O_2 solution in HFIP (1.53 mmol, 1.0 equiv), 10 mL of HFIP at 25 °C for 30 min. Purification by flash chromatography over silica gel (pentane-diethyl ether 50:1) afforded 65.9 mg of 1-ethyl-2-methylcyclobutyl acetate (**P3r-OAc**, 0.421 mmol, 28% yield) as colorless liquid. ^{1}H-NMR (400 MHz, $CDCl_3$) δ, ppm: 2.66 – 2.53 (m, 1H), 2.19 – 1.92 (m, 7H), 1.67 (dq, J = 14.8, 7.5 Hz, 1H), 1.24 – 1.15 (m, 1H), 1.12 (d, J = 7.0 Hz, 3H), 0.86 (t, J = 7.4 Hz, 3H). ^{13}C{^{1}H}-NMR (400 MHz, $CDCl_3$) δ, ppm: 169.78, 85.11, 41.13, 30.52, 23.08, 21.74, 15.29, 7.17. HRMS (ESI-MS) *m/z* calculated for $C_9H_{16}O_2$ $[M+Na]^+$ 179.1048, found 179.1051. The isolation of 1-(1-ethylcyclopropyl)ethyl acetate (**P3u-OAc**) wasn't accomplished due to the high volatility of the product. GC-MS (CI-NH_3): *m/z* for $C_9H_{16}O_2$ $[M+H]^+$ 157.1, $[M+NH_4]^+$ 174.1.

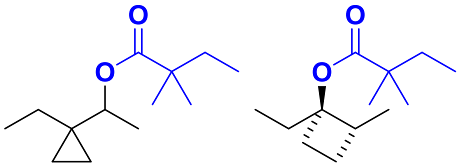

1-(1-ethylcyclopropyl)ethyl 2,2-dimethylbutanoate (P3u-OX$_7$) and 1-ethyl-2-methylcyclobutyl 2,2-dimethylbutanoate (P3r-OX$_7$). Prepared following the general procedure on 1.43 mmol of **S3**. General conditions: 14.2 mg of Mn(^{TIPS}pdp) (0.0143 mmol, 1 mol%), 140 mg of **S3** (1.43 mmol, 1.0 equiv), 5.4 ml of 2,2-dimethylbutanoic acid (43.1 mmol, 30 equiv), 4.03 ml of 0.709 M H_2O_2 solution in HFIP (1.53 mmol, 2.0 equiv), 15 mL of HFIP at 25 °C for 30 min. Purification by flash chromatography over silica gel (pentane-diethyl ether 50:1) afforded 69 mg of 1-(1-ethylcyclopropyl)ethyl 2,2-dimethylbutanoate (**P3u-OX$_7$**, 0.325 mmol, 23% yield) and 30.3 mg of 1-

ethyl-2-methylcyclobutyl 2,2-dimethylbutanoate (**P3r-OX$_7$**, 0.143 mmol, 10% yield) as colorless liquids.

P3u-OX$_7$: ^{1}H-NMR (400 MHz, $CDCl_3$) δ, ppm: 4.57 (q, J = 6.5 Hz, 1H), 1.70 (dq, J = 14.7, 7.4 Hz, 1H), 1.57 (d, J = 7.5 Hz, 2H), 1.23 (d, J = 6.4 Hz, 4H), 1.16 (d, J = 1.5 Hz, 6H), 0.94 (t, J = 7.5 Hz, 3H), 0.86 (t, J = 7.5 Hz, 3H), 0.50 – 0.40 (m, 2H), 0.39 – 0.30 (m, 2H). ^{13}C{^{1}H}-NMR (400 MHz, $CDCl_3$) δ, ppm: 177.45, 75.09, 42.68, 33.33, 25.94, 24.74, 24.67, 24.01, 17.04, 11.04, 9.76, 9.66, 9.24. HRMS (ESI-MS) *m/z* calculated for $C_9H_{16}O_2$ $[M+Na]^+$ 235.1669, found 235.1660.

P3r-OX$_7$: ^{1}H-NMR (400 MHz, $CDCl_3$) δ, ppm: 2.61 – 2.47 (m, 1H), 2.12 (dqd, J = 14.6, 7.3, 1.0 Hz, 1H), 2.05 – 1.94 (m, 3H), 1.73 – 1.63 (m, 1H), 1.58 – 1.54 (m, 1H), 1.25 – 1.18 (m, 1H), 1.16 – 1.12 (m, 9H), 0.87 (dt, J = 14.8, 7.4 Hz, 7H). ^{13}C{^{1}H}-NMR (400 MHz, $CDCl_3$) δ, ppm: 176.79, 84.76, 42.59, 41.18, 33.27, 30.35, 24.87, 24.58, 23.07, 21.76, 15.42, 9.29, 7.02. HRMS (ESI-MS) *m/z* calculated for $C_9H_{16}O_2$ $[M+Na]^+$ 235.1669, found 235.1672.

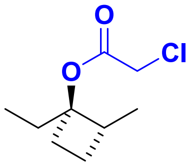

1-ethyl-2-methylcyclobutyl 2-chloroacetate (P3r-OX$_8$). Prepared following the general procedure on 1.0 mmol of **S3**. General conditions: 8 mg of Mn(^{CO_2Et}pdp) (0.01 mmol, 1 mol%), 100 mg of **S3** (1.0 mmol, 1.0 equiv), 2.8 g of chloroacetic acid (30 mmol, 30 equiv), 2.76 ml of 0.723 M H_2O_2 solution in HFIP (2.0 mmol, 2.0 equiv), 10 mL of HFIP at 25 °C for 30 min. Purification by flash chromatography over silica gel (pentane-diethyl ether 50:1) afforded 74.3 mg of 1-ethyl-2-methylcyclobutyl 2-chloroacetate (**P3r-OX$_8$**, 0.39 mmol, 39% yield) as colorless liquid. ^{1}H-NMR (400 MHz, $CDCl_3$) δ, ppm: 4.03 (s, 2H), 2.65 (tq, J = 9.4, 6.9 Hz, 1H), 2.20 – 2.05 (m, 3H), 2.00 (ddt, J = 10.8, 8.9, 4.9 Hz, 1H), 1.70 (dq, J = 14.9, 7.5 Hz, 1H), 1.23 (dd, J = 10.4, 9.2 Hz, 1H), 1.14 (d, J = 7.0 Hz, 3H), 0.88 (td, J = 7.4, 2.0 Hz, 3H). ^{13}C{^{1}H}-NMR (400 MHz, $CDCl_3$) δ, ppm: 165.74, 87.47, 41.41, 41.13, 30.26, 23.02, 21.55, 15.14, 7.04. GC-MS (CI-NH_3): *m/z* for $C_9H_{15}ClO_2$ $[M+H]^+$ 191.0, $[M+NH_4]^+$ 208.0.

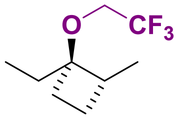

1-ethyl-2-methyl-1-(2,2,2-trifluoroethoxy)cyclobutane (P3r-OCH$_2$CF$_3$). Prepared following the general procedure on 1.7 mmol of **S3**. General conditions: 14 mg of Mn(^{CO_2Et}pdp) (0.017 mmol, 1 mol%), 167 mg of **S3** (1.7 mmol, 1.0 equiv), 2.28 ml of 1.12 M H_2O_2 solution in TFE (2.5 mmol, 1.5 equiv), 16 mL of TFE at 25 °C for 30 min. Purification by flash chromatography over silica gel in pure pentane afforded 96 mg of 1-ethyl-2-methyl-1-(2,2,2-trifluoroethoxy)cyclobutane (**P3r-OCH$_2$CF$_3$**, 0.49 mmol, 29% yield) as colorless liquid. ^{1}H-NMR (400 MHz, $CDCl_3$) δ, ppm: 3.68 (q, J = 8.7 Hz, 2H), 2.55 – 2.43 (m, 1H), 2.01 – 1.83 (m, 3H), 1.71 (dqd, J = 14.6, 7.2, 1.4 Hz, 1H), 1.51 (dt, J = 14.8, 7.4 Hz, 1H), 1.18 – 1.06 (m, 1H), 1.02 (d, J = 7.0 Hz, 3H), 0.92 (t, J = 7.3 Hz, 3H). ^{13}C{^{1}H}-NMR (400 MHz, $CDCl_3$) δ, ppm: 124.07, 82.49, 60.48, 38.61, 29.15, 22.43, 20.47, 15.35, 6.49. ^{19}F-NMR (400 MHz, $CDCl_3$) δ, ppm: -75.45. GC-MS (CI-NH_3): *m/z* for $C_9H_{15}F_3O$ $[M+H]^+$ 196.9, $[M+NH_4]^+$ 214.0.

GC-MS analysis of the reaction mixtures derived from the oxidation of **S4** in the conditions displayed in **Scheme S6** gave the following spectrometric data:

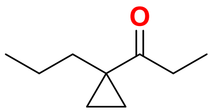

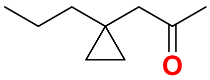

1-(1-propylcyclopropyl)propan-1-one (**P4-O**): GC-MS (CI-NH_3): *m/z* for $C_9H_{16}O$ $[M+H]^+$ 141.1, $[M+NH_4]^+$ 158.1.

1-(1-propylcyclopropyl)propan-2-one (**P4-O$_\beta$):** GC-MS (CI-NH_3): *m/z* for $C_9H_{16}O$ $[M+H]^+$ 141.1, $[M+NH_4]^+$ 158.1.

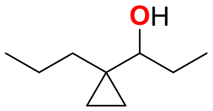

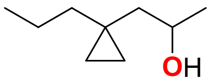

1-(1-propylcyclopropyl)propan-1-one (**P4u-OH**): GC-MS (CI-NH_3): *m/z* for $C_9H_{18}O$ $[M+H]^+$ 143.2, $[M+NH_4]^+$ 160.1.

1-(1-propylcyclopropyl)propan-2-one (**P4-OH$_\beta$):** GC-MS (CI-NH_3): *m/z* for $C_9H_{18}O$ $[M+H]^+$ 143.0, $[M+NH_4]^+$ 160.1.

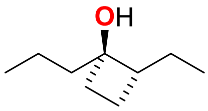

2-ethyl-1-propylcyclobutan-1-ol (**P4r-OH**): GC-MS (CI-NH_3): *m/z* for $C_9H_{18}O$ $[M+H]^+$ 143.0, $[M+NH_4]^+$ 160.1.

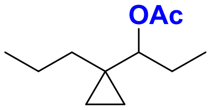

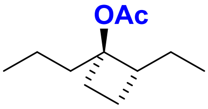

1-(1-propylcyclopropyl)propyl acetate (**P4u-OAc**): GC-MS (CI-NH_3): *m/z* for $C_{11}H_{20}O_2$ $[M+H]^+$ 185.1, $[M+NH_4]^+$ 202.1.

2-ethyl-1-propylcyclobutyl acetate (**P4r-OAc**): GC-MS (CI-NH_3): *m/z* for $C_{11}H_{20}O_2$ $[M+H]^+$ 185.1, $[M+NH_4]^+$ 202.1.

GC-MS analysis of the reaction mixtures derived from the oxidation of **S5** in the conditions displayed in **Scheme S7** gave the following spectrometric data:

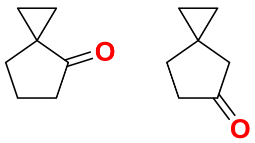

Spiro[2.4]heptan-4-one (**P5-O**): GC-MS (CI-NH_3): *m/z* for $C_7H_{10}O$ $[M+H]^+$ 111.0, $[M+NH_4]^+$ 128.0.
Spiro[2.4]heptan-5-one (**P5-O$_β$**)**:** GC-MS (CI-NH_3): *m/z* for $C_7H_{10}O$ $[M+H]^+$ 110.9, $[M+NH_4]^+$ 128.0.

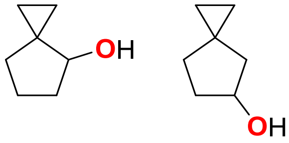

Spiro[2.4]heptan-4-ol (**P5u-OH**): GC-MS (CI-NH_3): *m/z* for $C_7H_{12}O$ $[M+H]^+$ 113.0, $[M+NH_4]^+$ 129.9.
Spiro[2.4]heptan-5-ol (**P5-OH$_β$**)**:** GC-MS (CI-NH_3): *m/z* for $C_7H_{12}O$ $[M+H]^+$ 113.0, $[M+NH_4]^+$ 130.0.

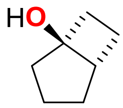

Bicyclo[3.2.0]heptan-1-ol (**P5r-OH**): GC-MS (CI-NH_3): *m/z* for $C_7H_{12}O$ $[M+H]^+$ 113.0, $[M+NH_4]^+$ 130.0.

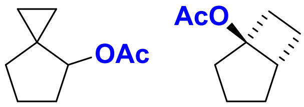

Spiro[2.4]heptan-4-yl acetate (**P5u-OAc**): GC-MS (CI-NH_3): *m/z* for $C_7H_{12}O$ $[M+H]^+$ 155.0, $[M+NH_4]^+$ 172.0.
Bicyclo[3.2.0]heptan-1-yl acetate (**P5-OAc**)**:** GC-MS (CI-NH_3): *m/z* for $C_9H_{14}O_2$ $[M+H]^+$ 155.0, $[M+NH_4]^+$ 172.0.

6. NMR spectra

6.1. NMR spectra of the substrates

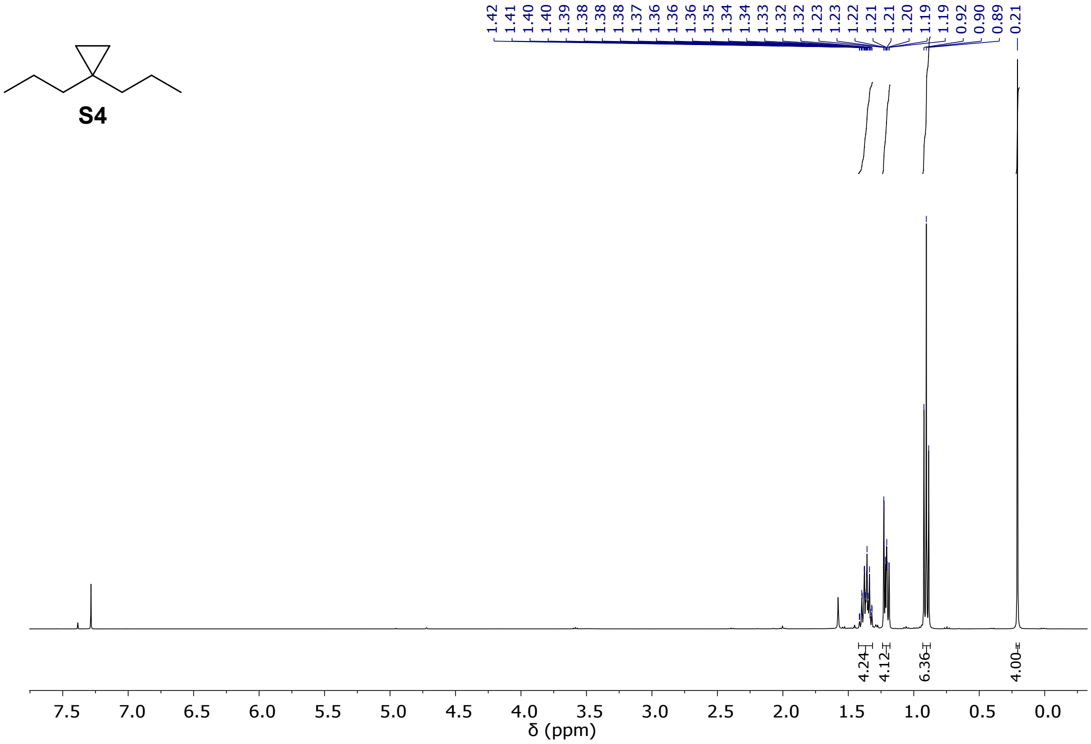

Figure S2. ^{1}H-NMR spectrum (400 MHz, $CDCl_3$) of 1,1-dipropylcyclopropane (**S4**).

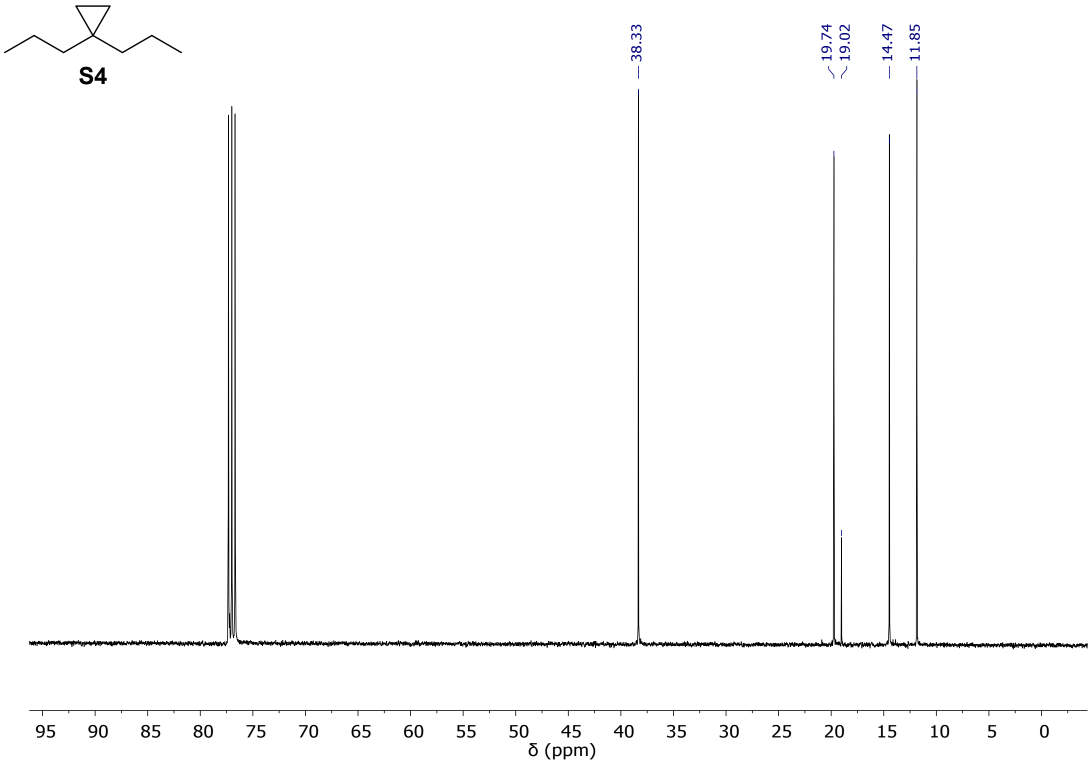

Figure S3. $^{13}C\{^{1}H\}$-NMR spectrum (400 MHz, $CDCl_3$) of 1,1-dipropylcyclopropane (**S4**).

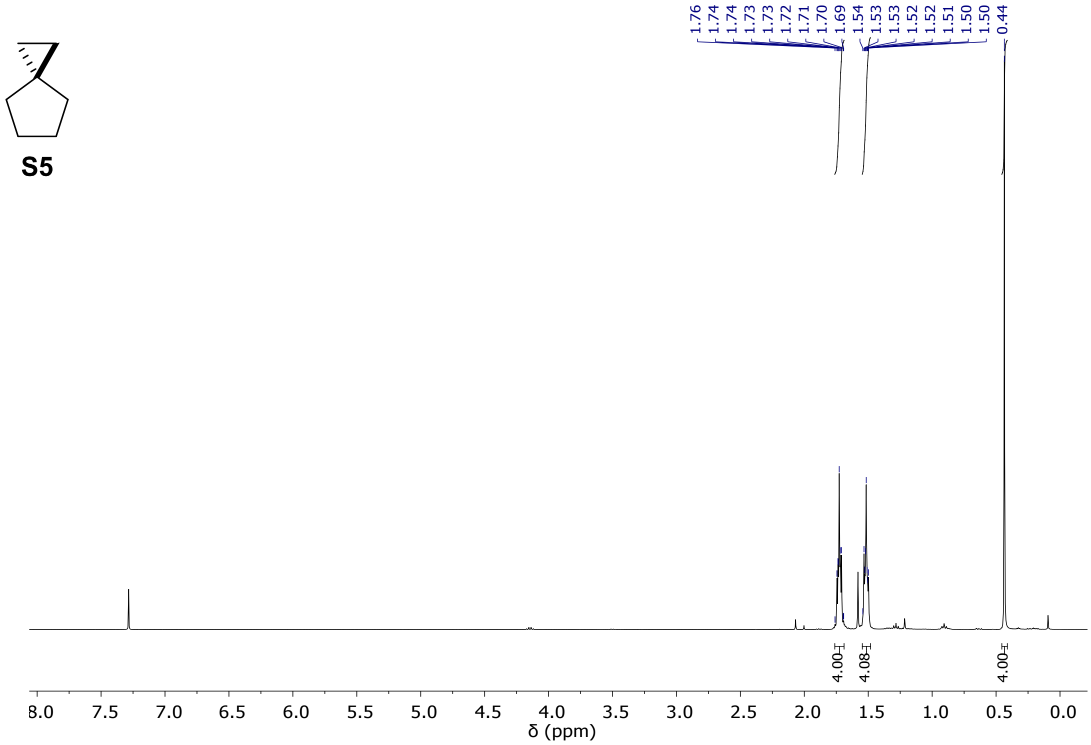

Figure S4. ^{1}H-NMR spectrum (400 MHz, $CDCl_3$) of spiro[2.4]heptane (**S5**).

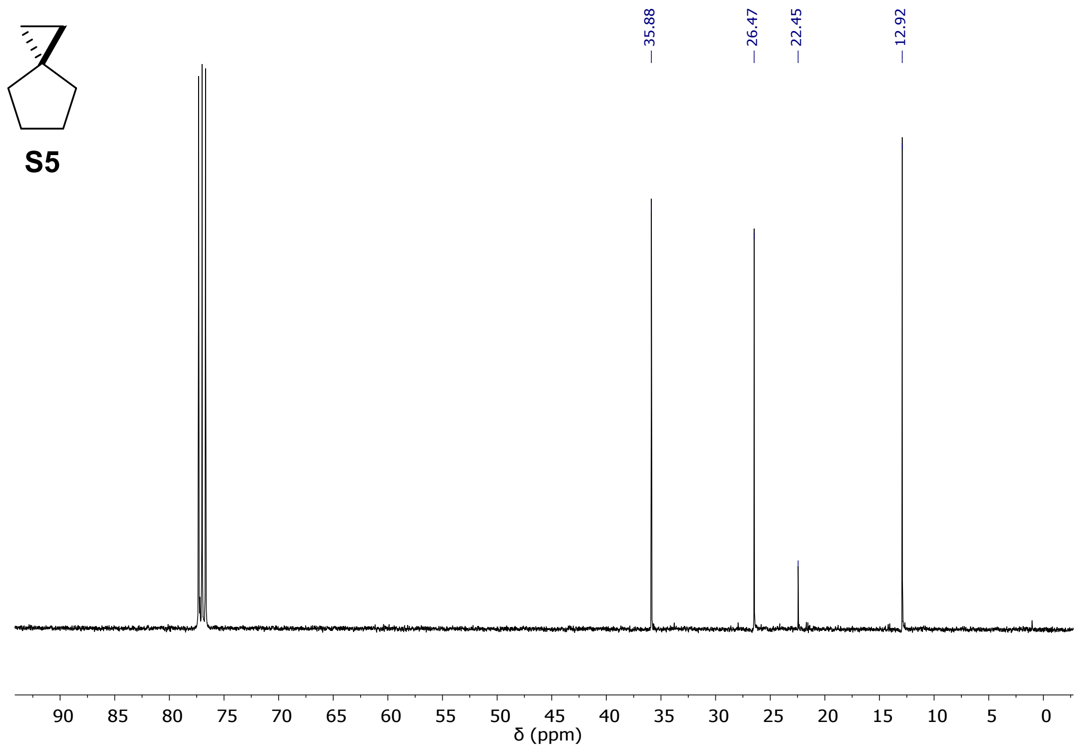

Figure S5. $^{13}C\{^{1}H\}$-NMR spectrum (400 MHz, $CDCl_3$) of spiro[2.4]heptane (**S5**).

6.2. NMR spectra of the ligands

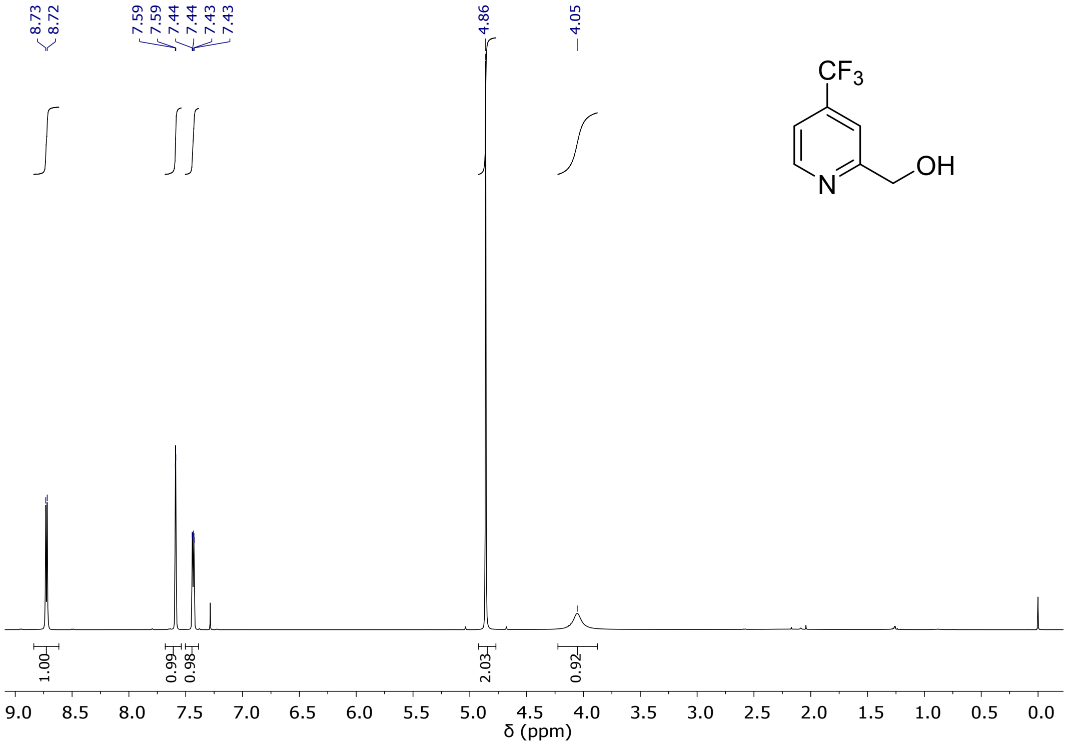

Figure S6. ^{1}H-NMR spectrum (400 MHz, $CDCl_3$) of (4-(trifluoromethyl)pyridin-2-yl)methanol.

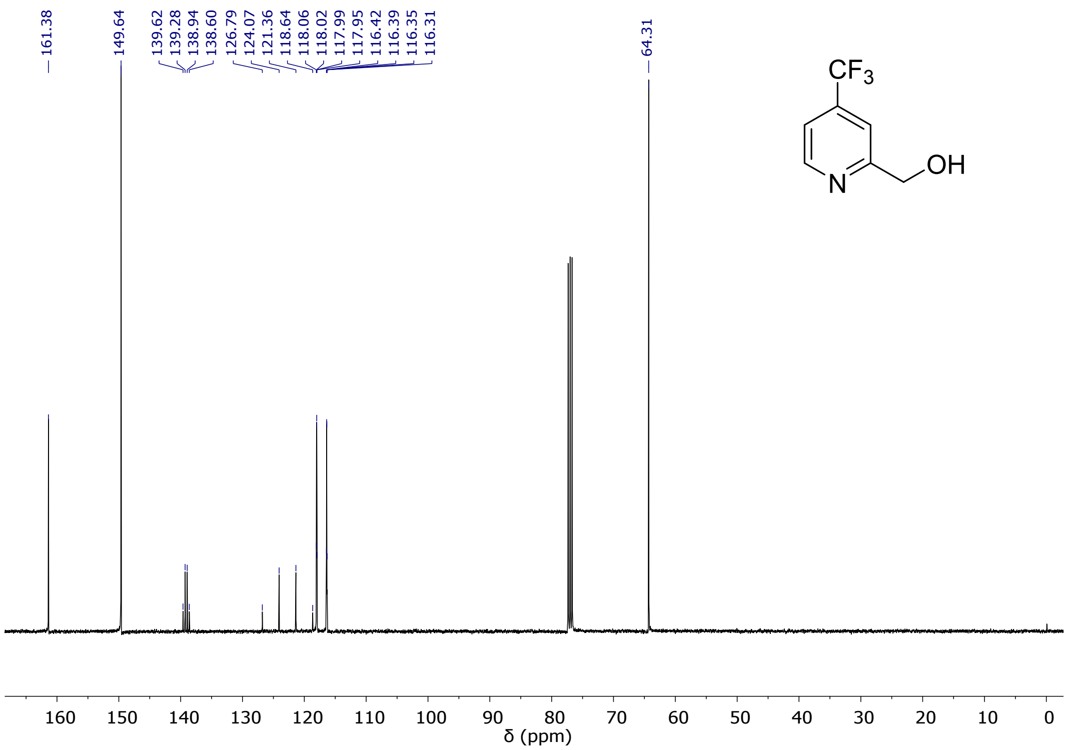

Figure S7. $^{13}C\{^{1}H\}$-NMR spectrum (400 MHz, $CDCl_3$) of (4-(trifluoromethyl)pyridin-2-yl)methanol.

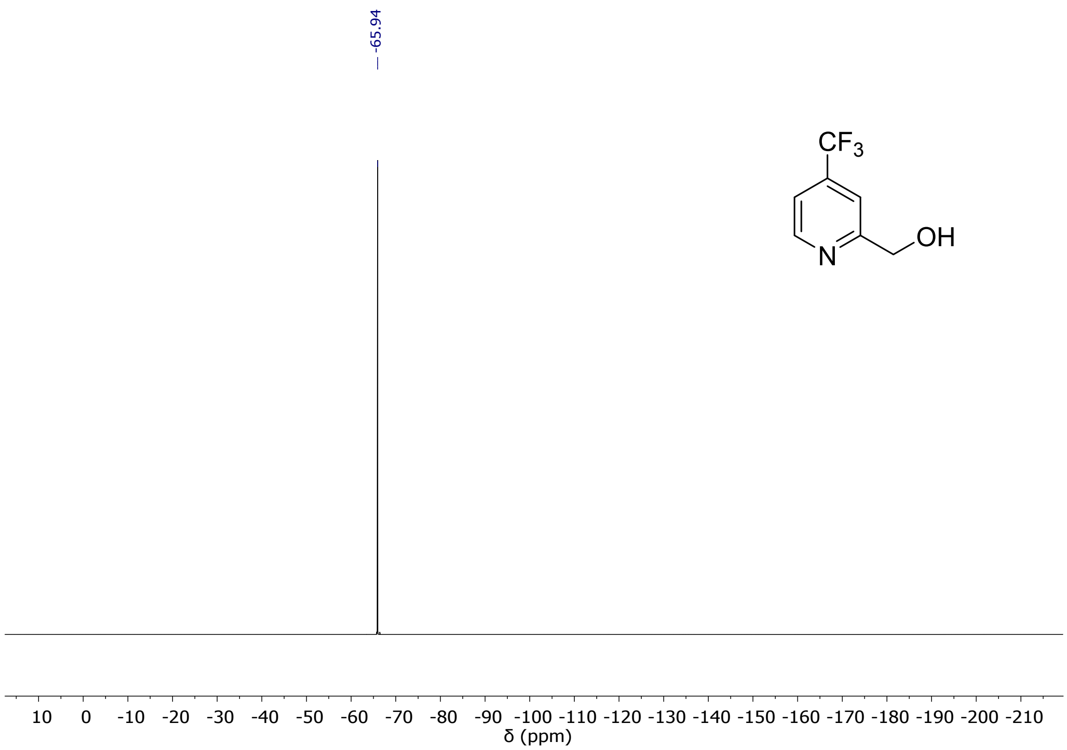

Figure S8. $^{19}F\{^{1}H\}$-NMR spectrum (400 MHz, $CDCl_3$) of (4-(trifluoromethyl)pyridin-2-yl)methanol.

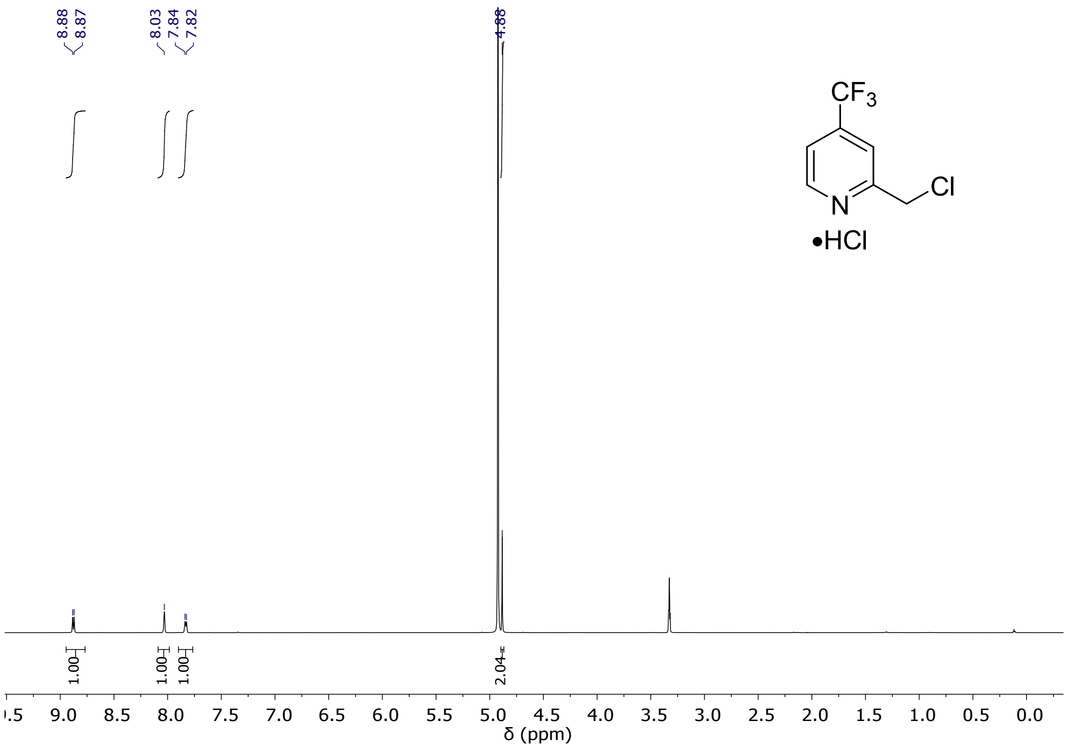

Figure S9. ^{1}H-NMR spectrum (400 MHz, CD_3OD) of 2-(chloromethyl)-4-(trifluoromethyl)pyridine hydrochloride.

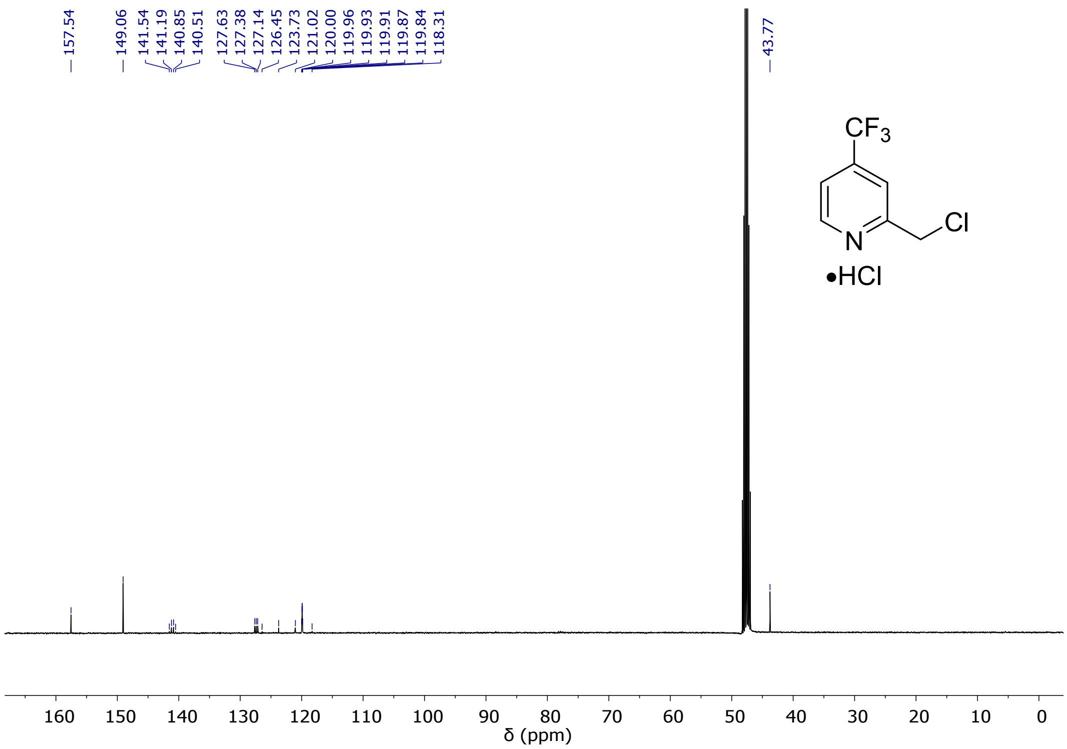

Figure S10. $^{13}C\{^{1}H\}$-NMR spectrum (400 MHz, CD_3OD) of 2-(chloromethyl)-4-(trifluoromethyl)pyridine hydrochloride.

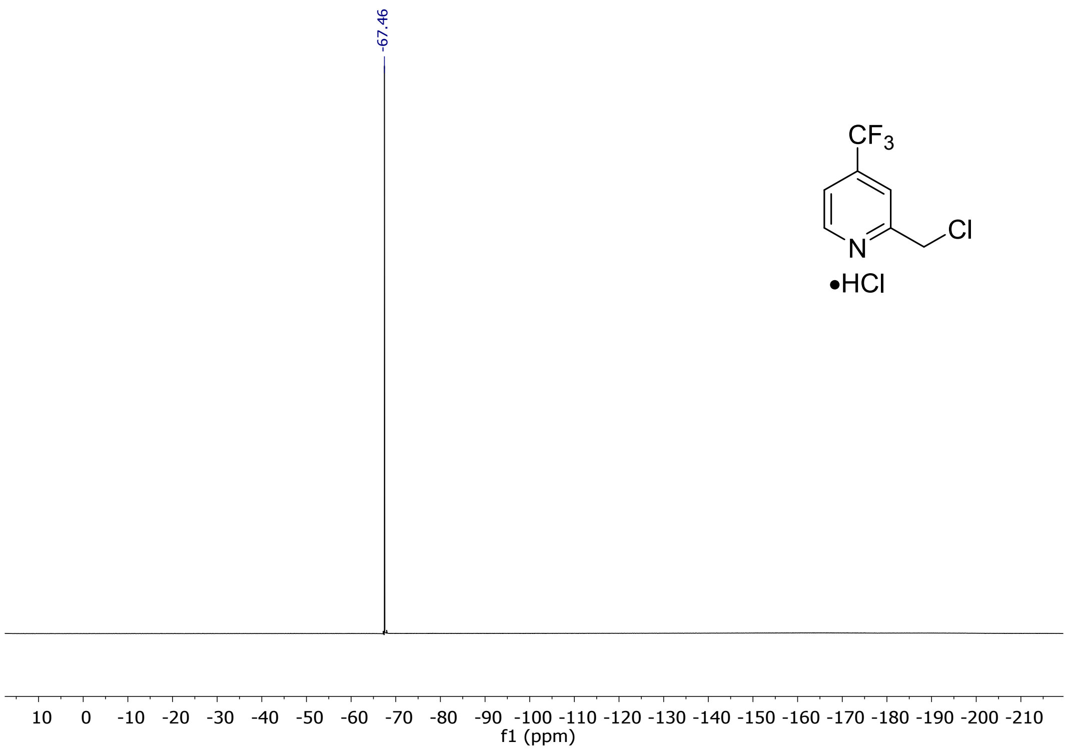

Figure S11. $^{19}F\{^{1}H\}$-NMR spectrum (400 MHz, CD_3OD) of 2-(chloromethyl)-4-(trifluoromethyl)pyridine hydrochloride.

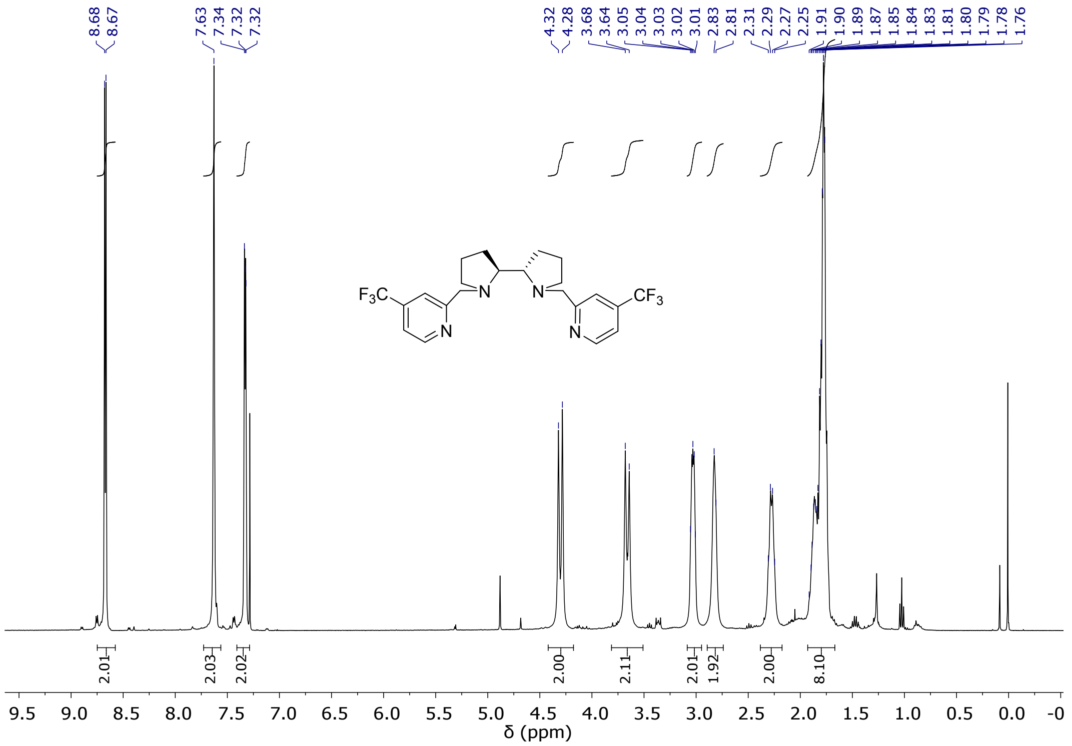

Figure S12. ^{1}H-NMR spectrum (400 MHz, $CDCl_3$) of ***(R,R)*-^{CF_3}pdp**.

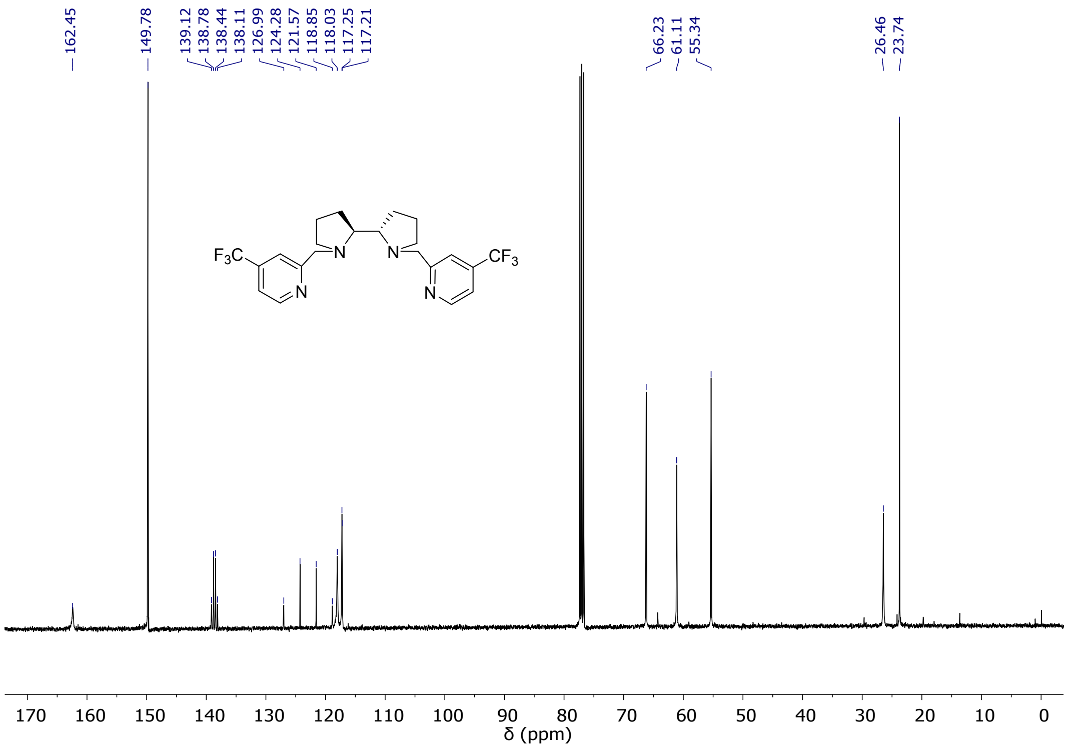

Figure S13. $^{13}C\{^{1}H\}$-NMR spectrum (400 MHz, $CDCl_3$) of ***(R,R)*-^{CF_3}pdp**.

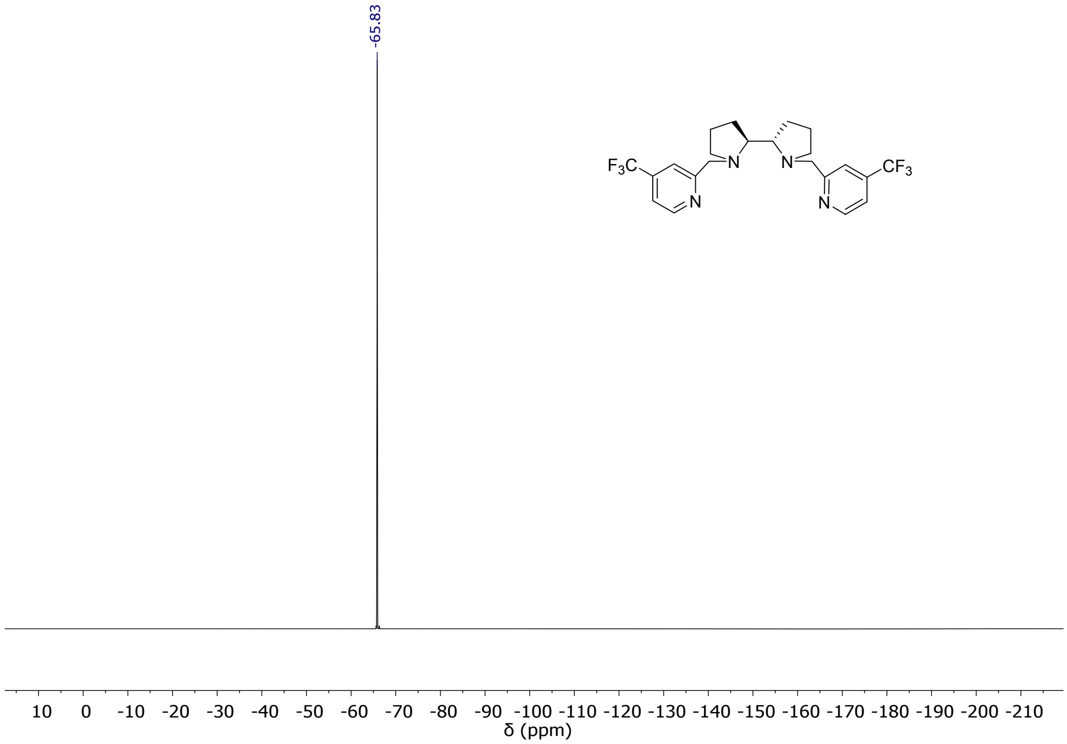

Figure S14. ^{19}F{^{1}H}-NMR spectrum (400 MHz, $CDCl_3$) of **(*R*,*R*)-^{CF_3}pdp**.

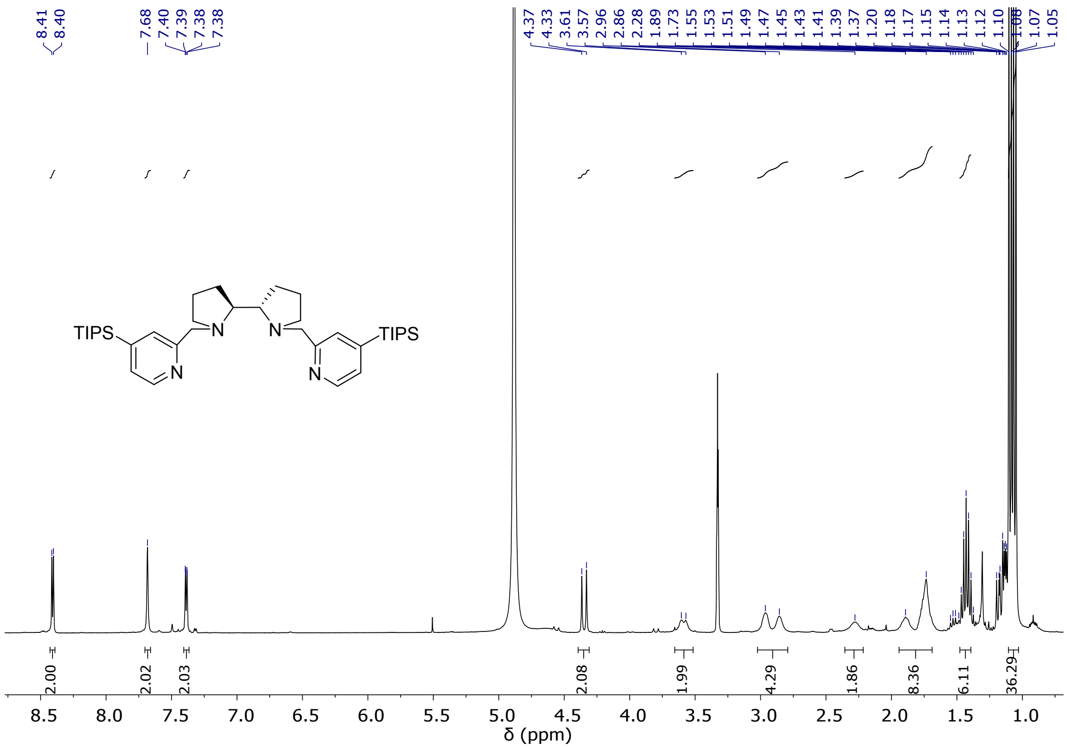

Figure S15. ^{1}H-NMR spectrum (400 MHz, CD_3OD) of **(*R*,*R*)-$^{p\text{-}TIPS}$pdp**.

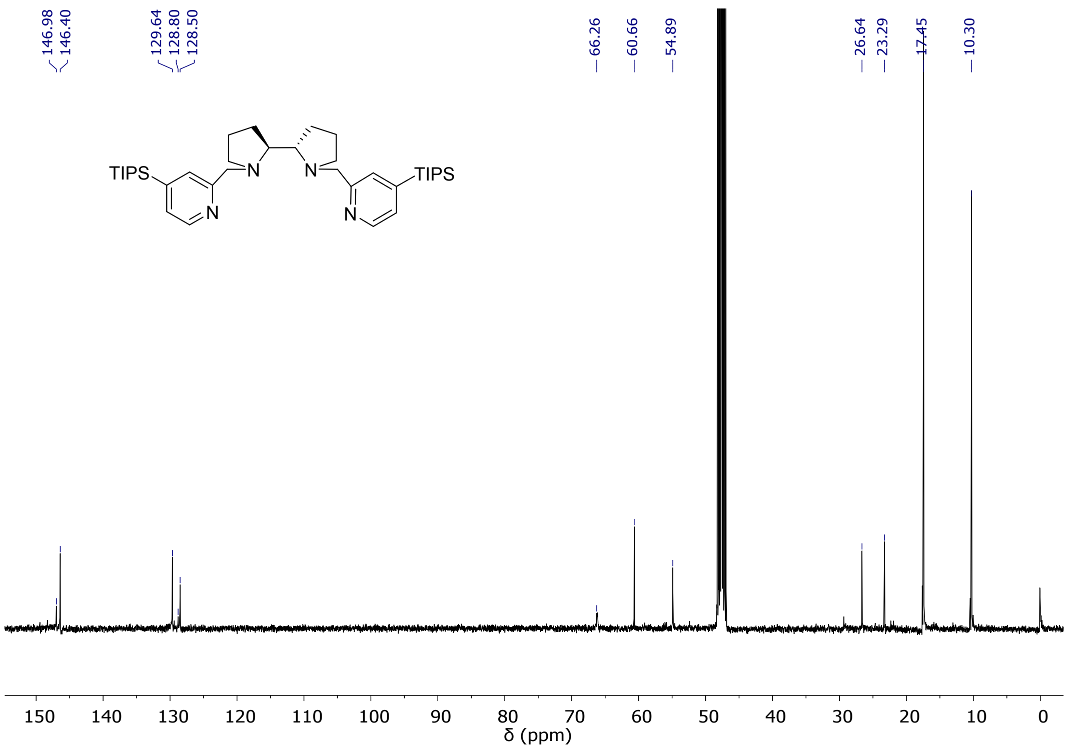

Figure S16. $^{13}C\{^{1}H\}$-NMR spectrum (400 MHz, CD_3OD) of **(*R*,*R*)-*p*-TIPSpdp**.

6.3. NMR spectra of the isolated products

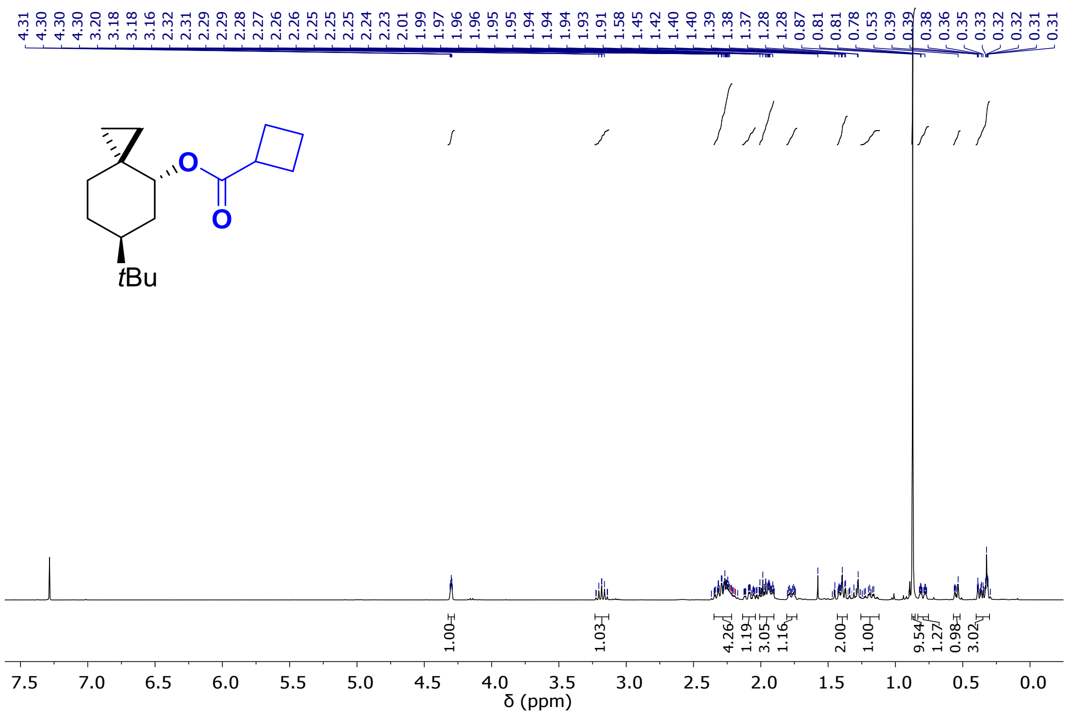

Figure S17. ^{1}H-NMR spectrum (400 MHz, $CDCl_3$) of *trans*-6-(*tert*-butyl)spiro[2.5]octan-4-yl cyclobutanecarboxylate (**P1u-OX$_4$**).

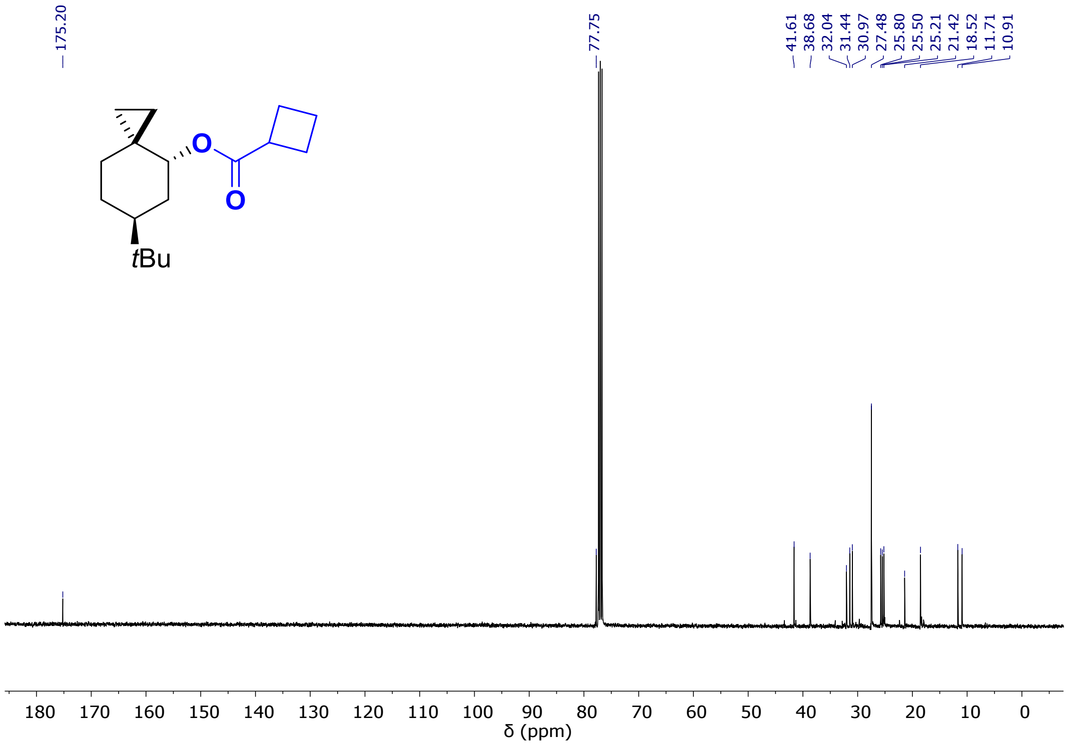

Figure S18. $^{13}C\{^1H\}$-NMR spectrum (400 MHz, $CDCl_3$) of *trans*-6-(*tert*-butyl)spiro[2.5]octan-4-yl cyclobutanecarboxylate (**P1u-OX$_4$**).

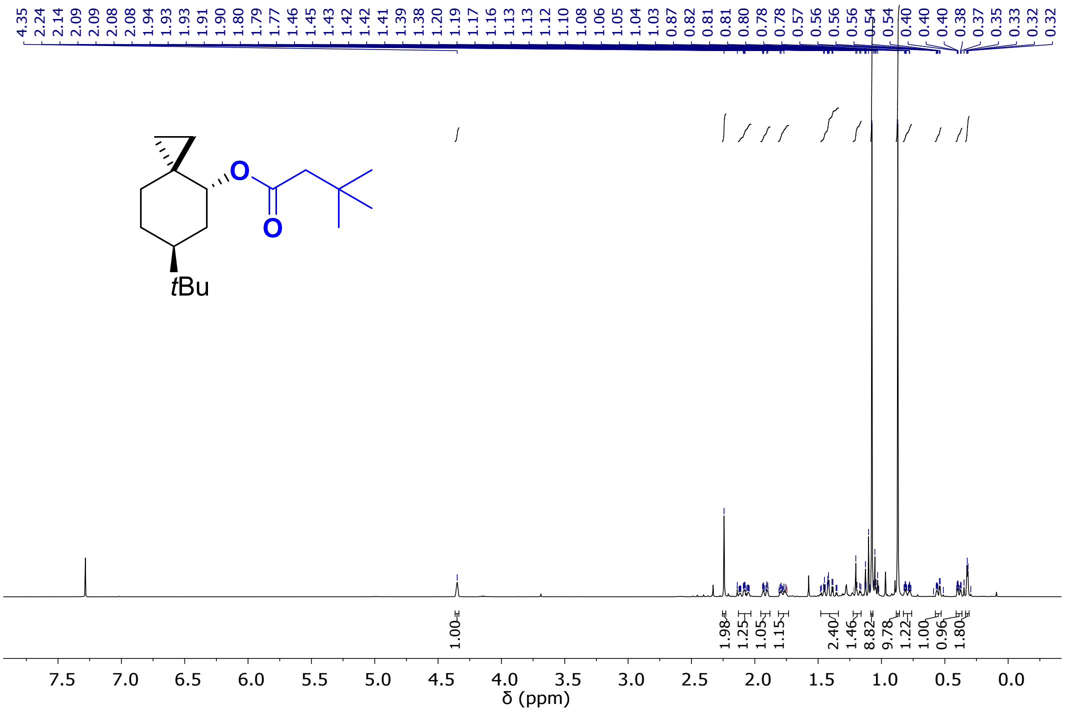

Figure S19. ^{1}H-NMR spectrum (400 MHz, $CDCl_3$) of *trans*-6-(*tert*-butyl)spiro[2.5]octan-4-yl 3,3-dimethylbutanoate (**P1u-OX$_6$**).

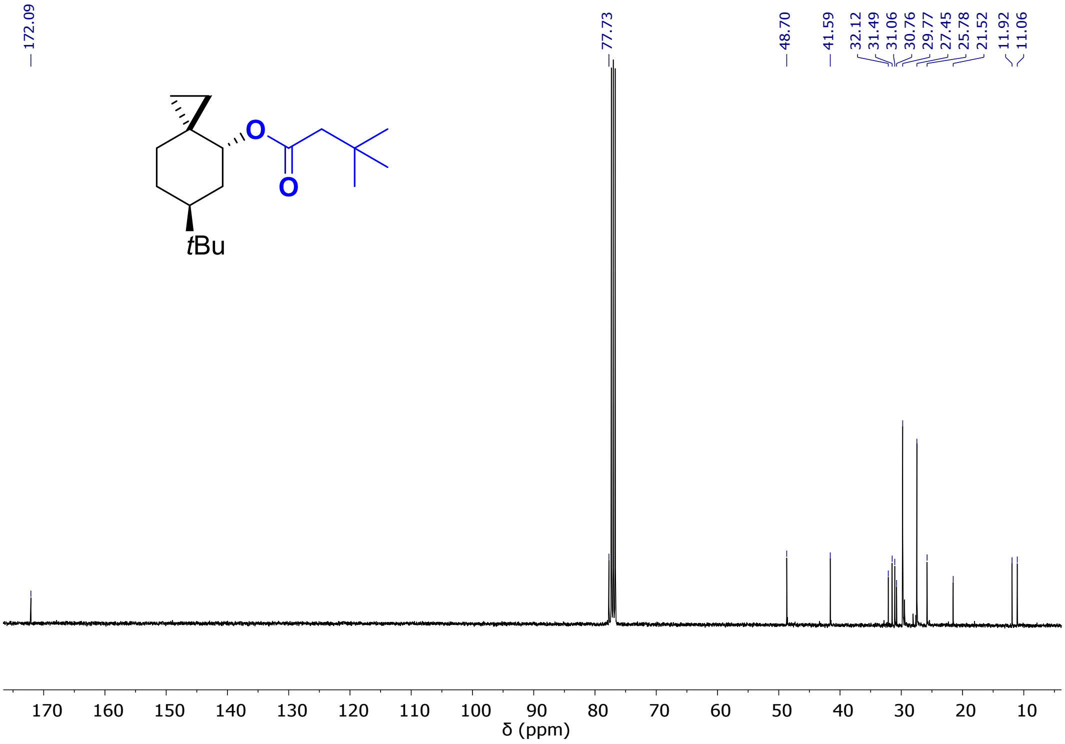

Figure S20. $^{13}C\{^1H\}$-NMR spectrum (400 MHz, $CDCl_3$) of *trans*-6-(*tert*-butyl)spiro[2.5]octan-4-yl 3,3-dimethylbutanoate (**P1u-OX$_6$**).

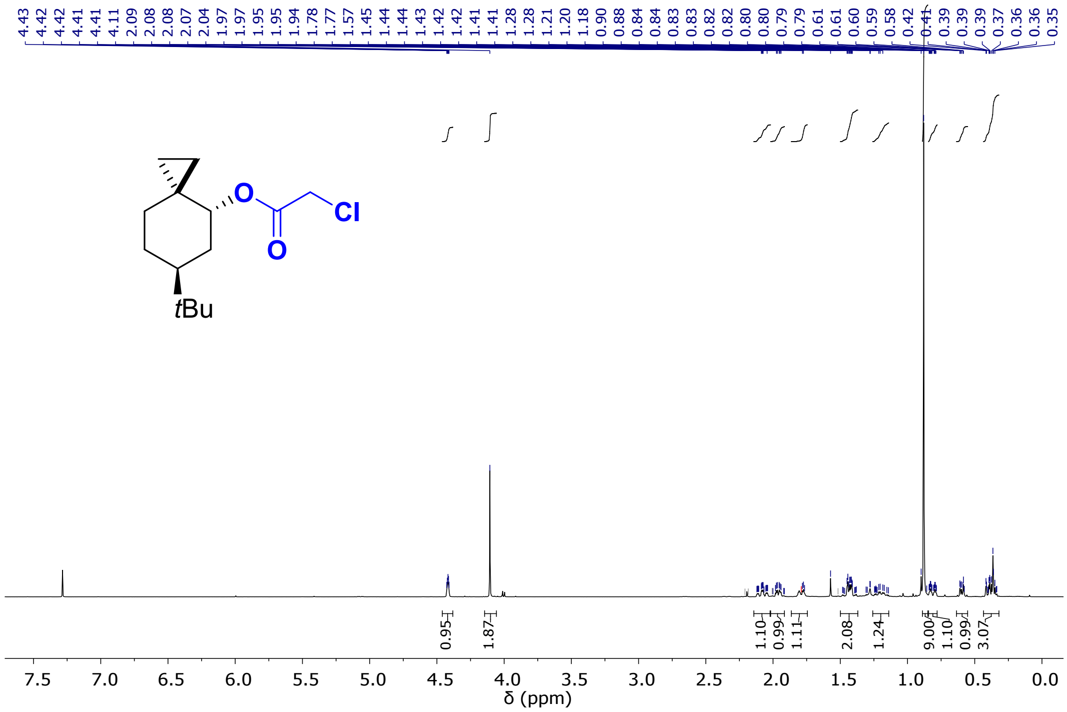

Figure S21. ^{1}H-NMR spectrum (400 MHz, $CDCl_3$) of *trans*-6-(*tert*-butyl)spiro[2.5]octan-4-yl 2-chloroacetate (**P1u-OX$_8$**).

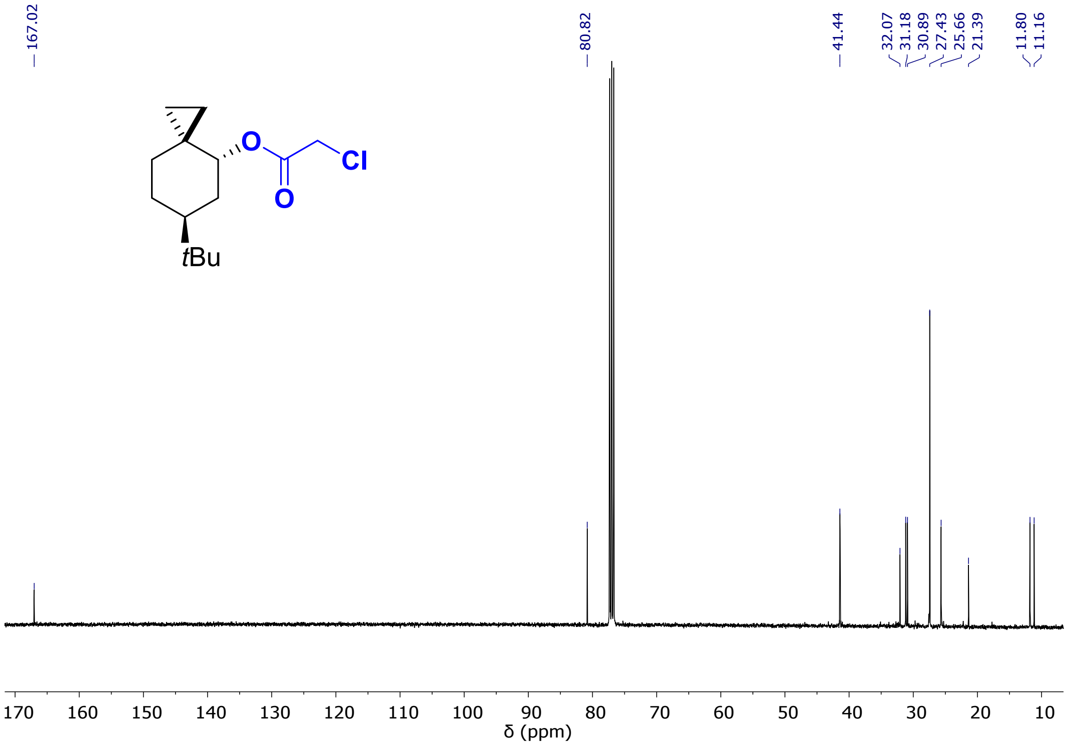

Figure S22. ^{13}C{^{1}H}-NMR spectrum (400 MHz, $CDCl_3$) of *trans*-6-(*tert*-butyl)spiro[2.5]octan-4-yl 2-chloroacetate (**P1u-OX$_8$**).

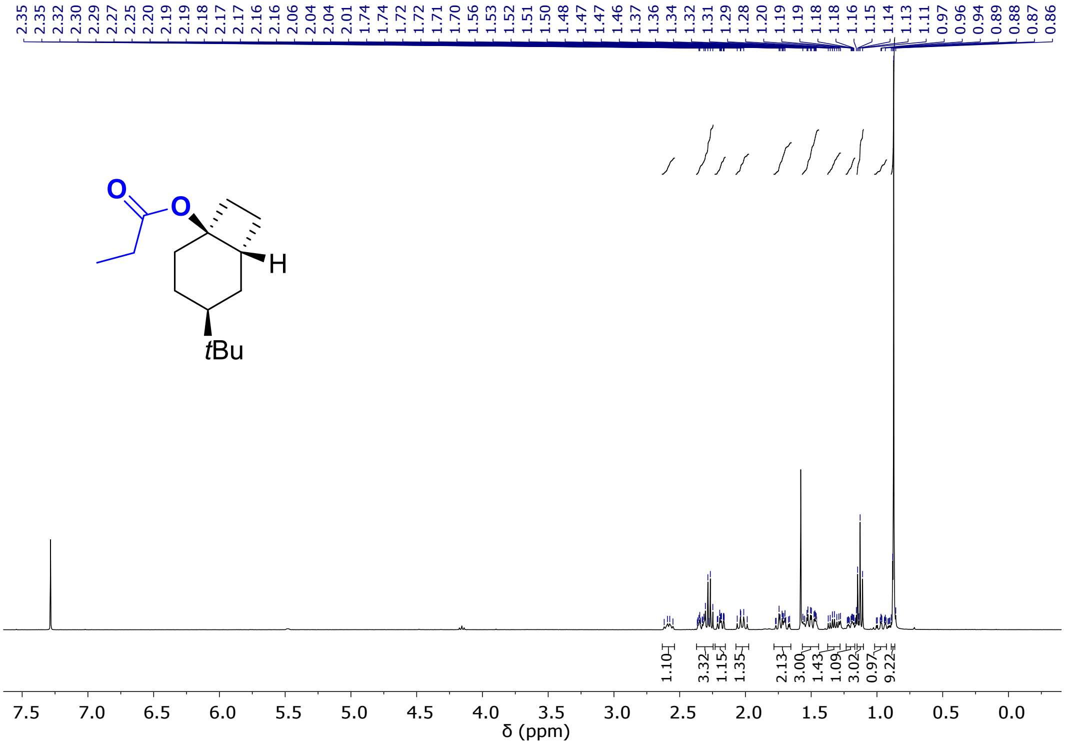

Figure S23. ^{1}H-NMR spectrum (400 MHz, $CDCl_3$) of *cis*-4-*tert*-butylbicyclo[4.2.0]octan-1-yl propionate (**P1r-OX$_2$**).

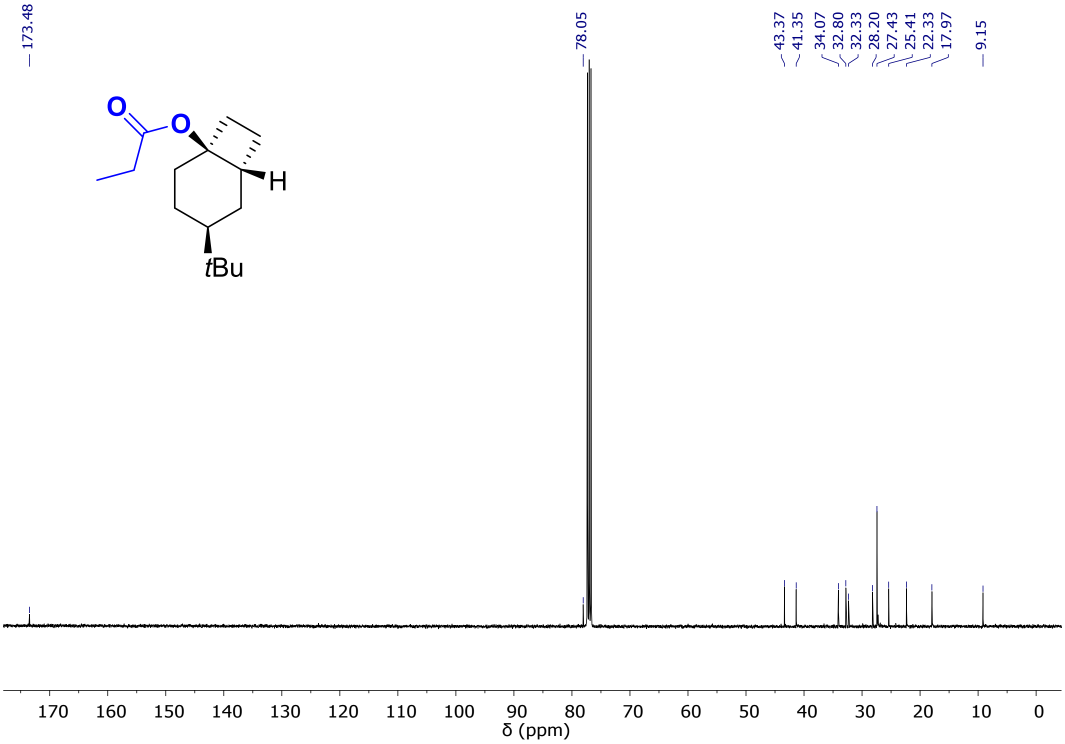

Figure S24. ^{13}C{^{1}H}-NMR spectrum (400 MHz, $CDCl_3$) of *cis*-4-*tert*-butylbicyclo[4.2.0]octan-1-yl propionate (**P1r-OX$_2$**).

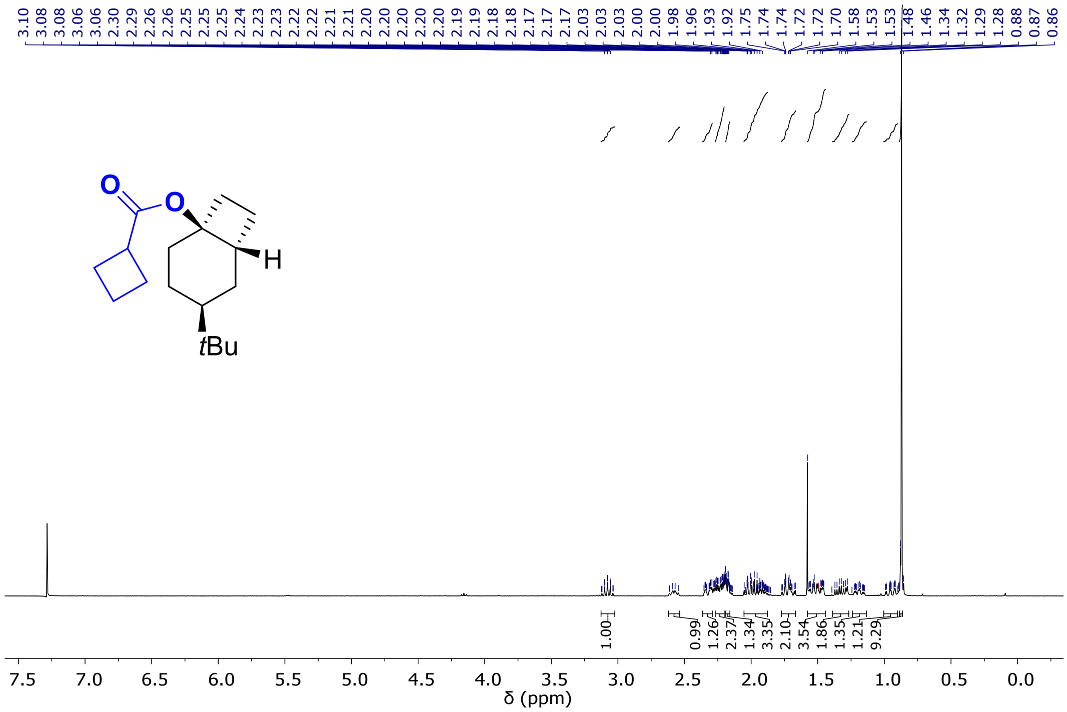

Figure S25. ^{1}H-NMR spectrum (400 MHz, $CDCl_3$) of *cis*-4-*tert*-butylbicyclo[4.2.0]octan-1-yl cyclobutanecarboxylate (**P1r-OX$_4$**).

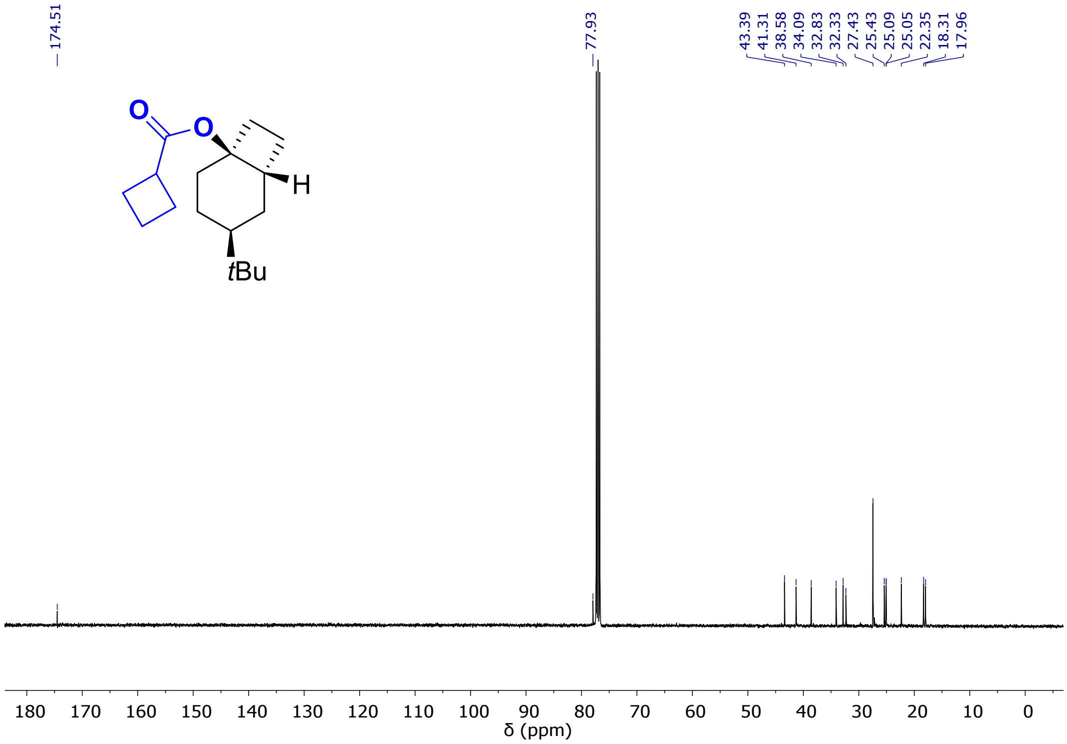

Figure S26. ^{13}C{^{1}H}-NMR spectrum (400 MHz, $CDCl_3$) of *cis*-4-*tert*-butylbicyclo[4.2.0]octan-1-yl cyclobutanecarboxylate (**P1r-OX$_4$**).

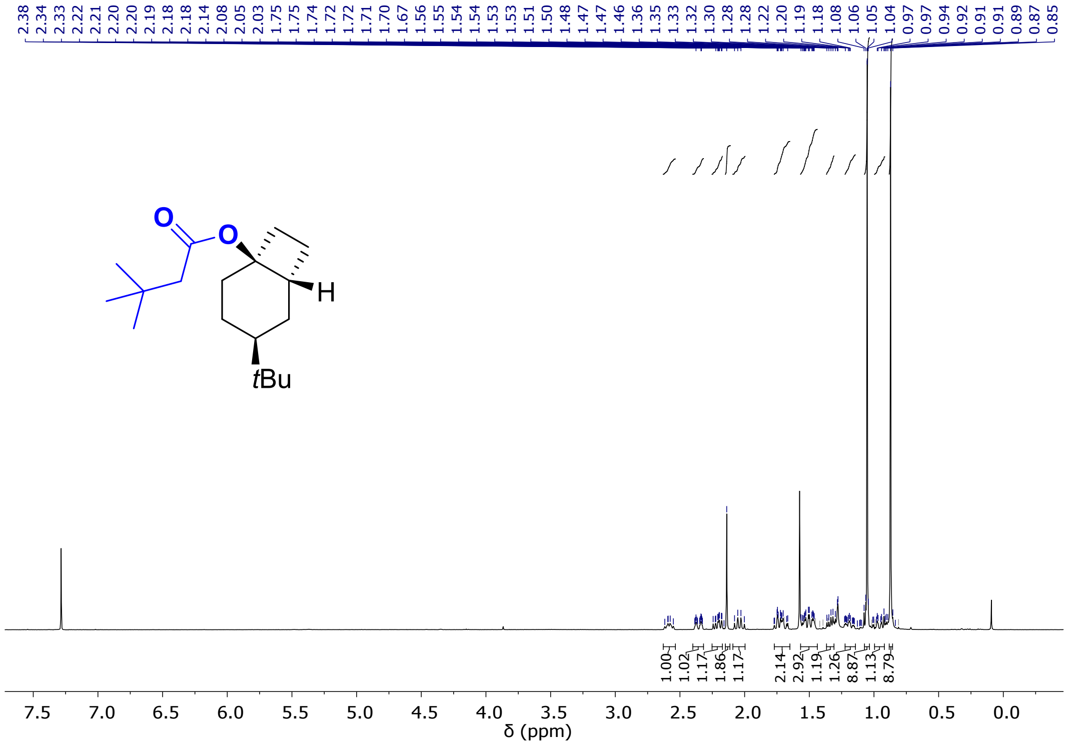

Figure S27. ^{1}H-NMR spectrum (400 MHz, $CDCl_3$) of *cis*-4-*tert*-butylbicyclo[4.2.0]octan-1-yl 3,3-dimethylbutanoate (**P1r-OX$_6$**).

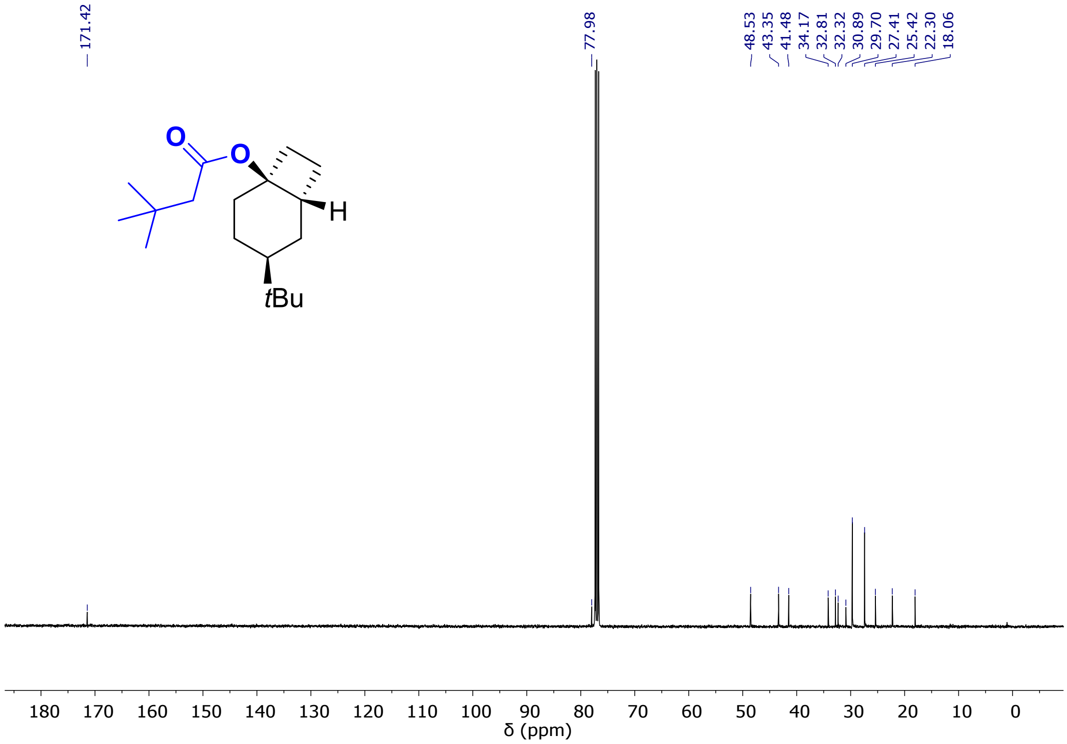

Figure S28. $^{13}C\{^{1}H\}$-NMR spectrum (400 MHz, $CDCl_3$) of *cis*-4-*tert*-butylbicyclo[4.2.0]octan-1-yl 3,3-dimethylbutanoate (**P1r-OX$_6$**).

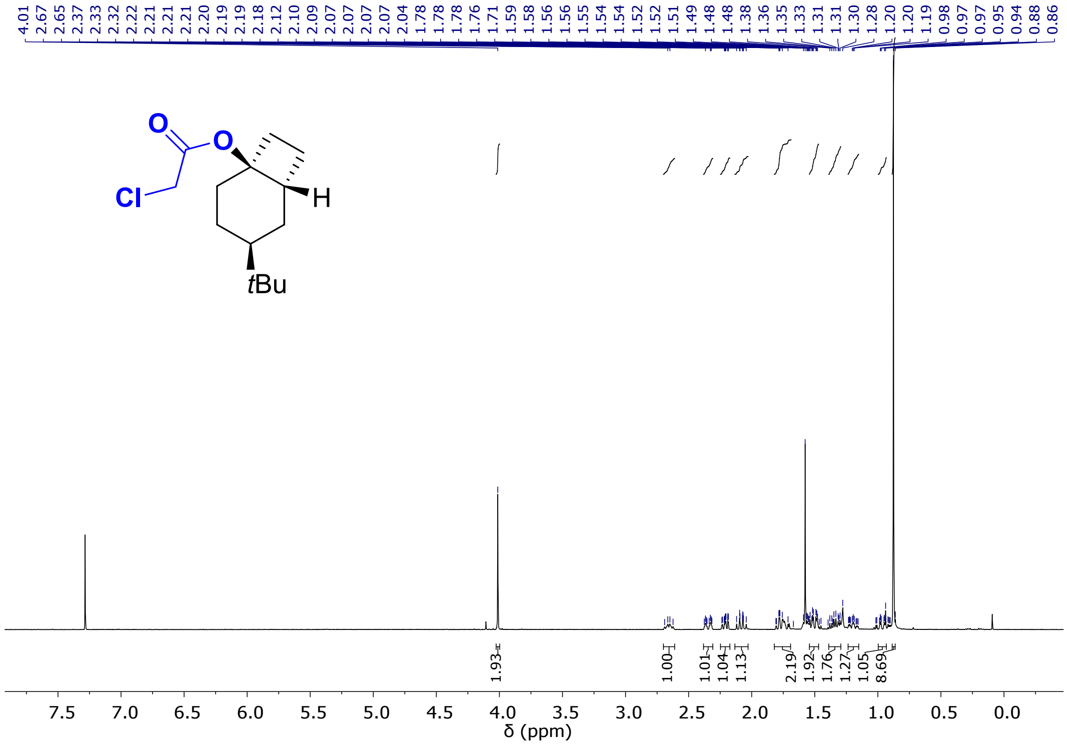

Figure S29. ^{1}H-NMR spectrum (400 MHz, $CDCl_3$) of *cis*-4-*tert*-butylbicyclo[4.2.0]octan-1-yl 2-chloroacetate (**P1r-OX$_8$**).

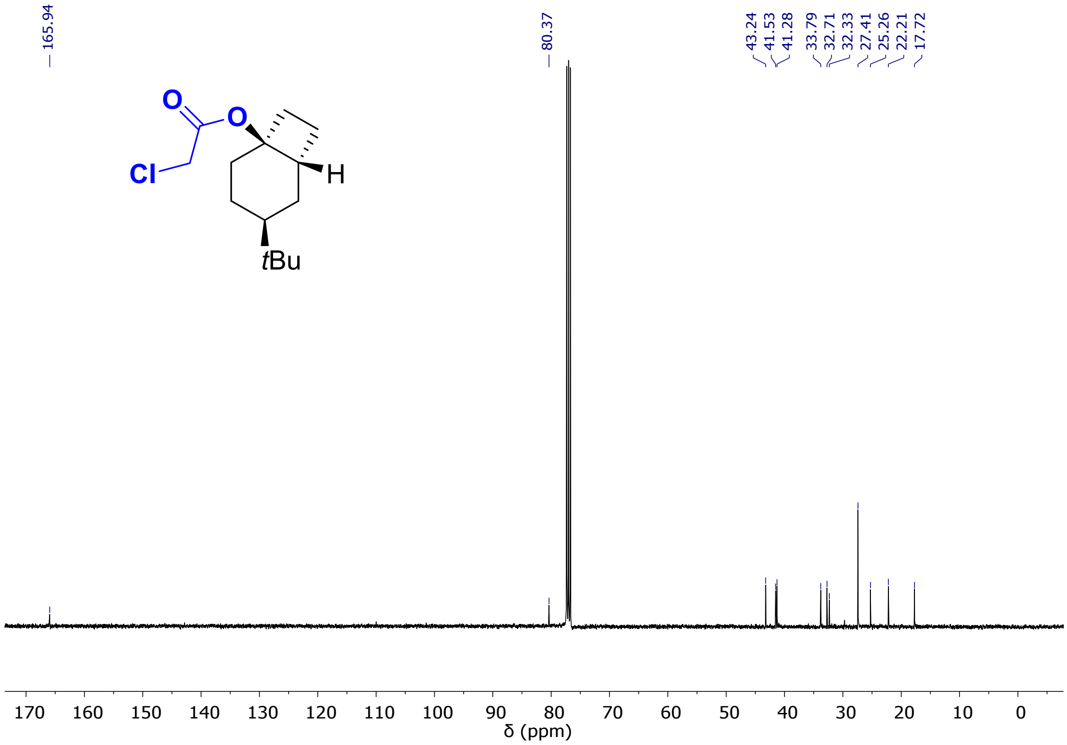

Figure S30. $^{13}C\{^{1}H\}$-NMR spectrum (400 MHz, $CDCl_3$) of *cis*-4-*tert*-butylbicyclo[4.2.0]octan-1-yl 2-chloroacetate (**P1r-OX$_8$**).

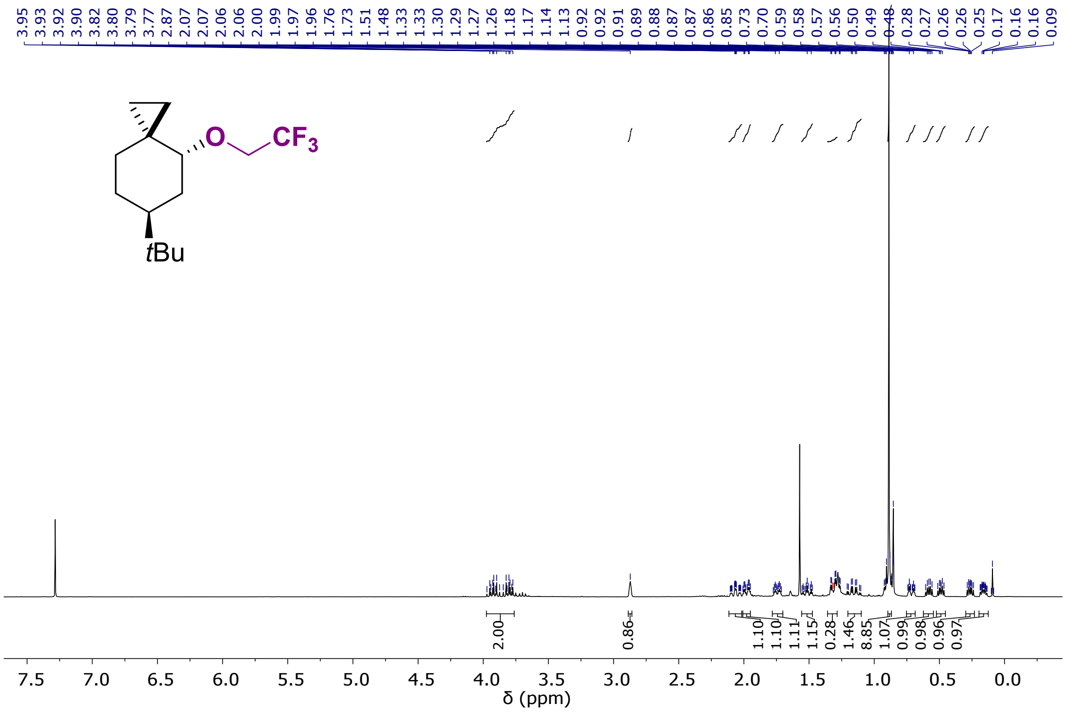

Figure S31. ^{1}H-NMR spectrum (400 MHz, $CDCl_3$) of *trans*-6-(*tert*-butyl)-4-(2,2,2-trifluoroethoxy)spiro[2.5]octane (**P1u-OCH$_2$CF$_3$**).

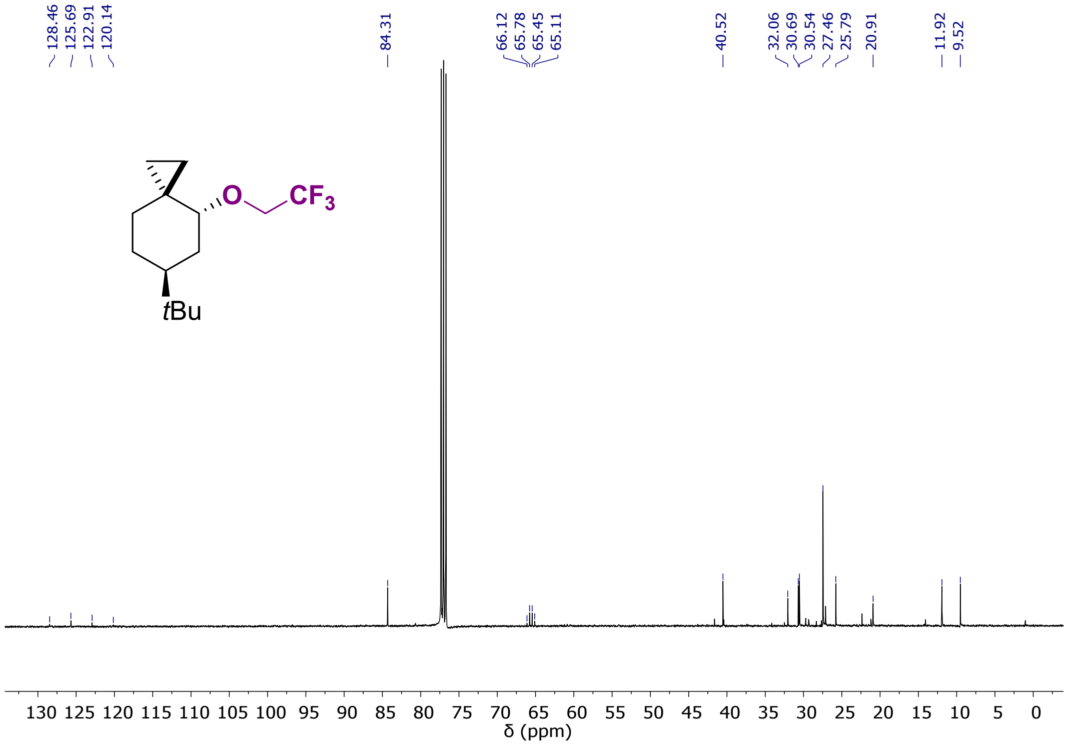

Figure S32. $^{13}C\{^{1}H\}$-NMR spectrum (400 MHz, $CDCl_3$) of *trans*-6-(*tert*-butyl)-4-(2,2,2-trifluoroethoxy)spiro[2.5]octane (**P1u-OCH$_2$CF$_3$**).

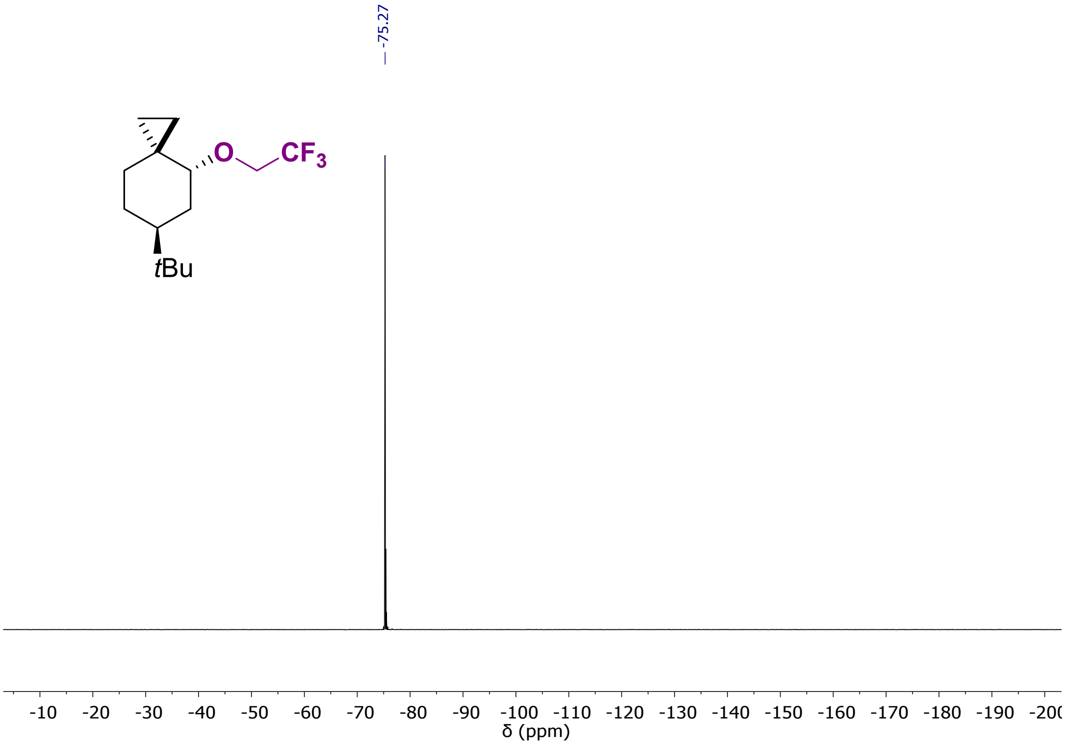

Figure S33. $^{19}F\{^{1}H\}$-NMR spectrum (400 MHz, $CDCl_3$) of *trans*-6-(*tert*-butyl)-4-(2,2,2-trifluoroethoxy)spiro[2.5]octane (**P1u-OCH$_2$CF$_3$**).

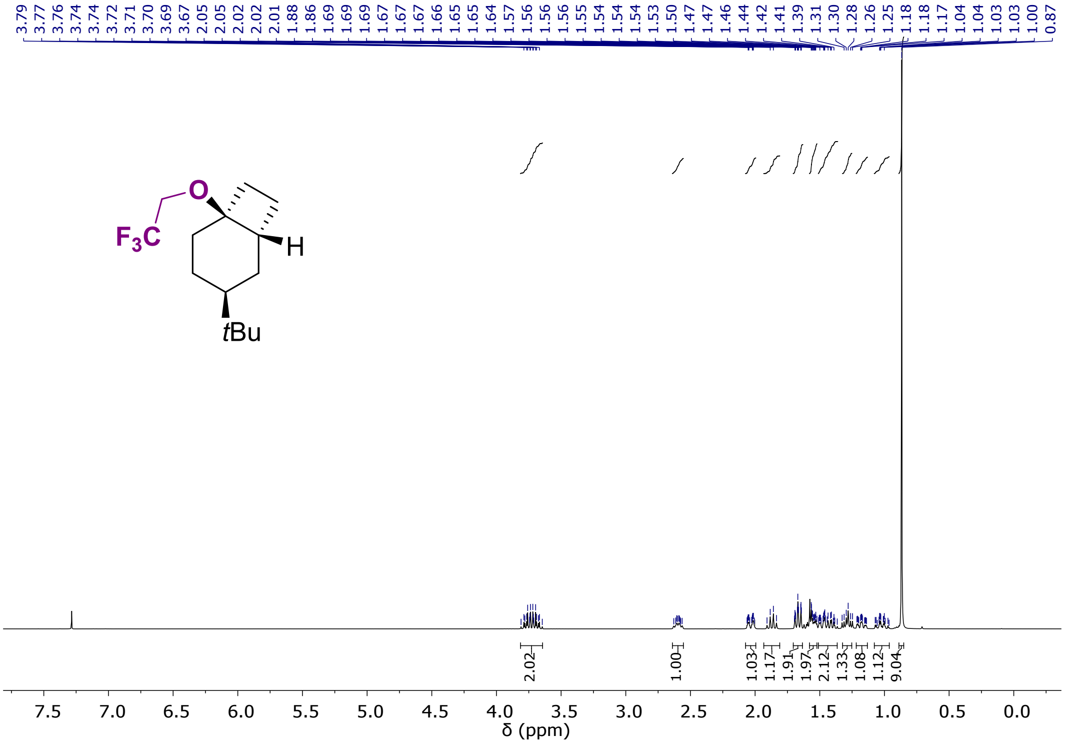

Figure S34. ^{1}H-NMR spectrum (400 MHz, $CDCl_3$) of *cis*-4-(*tert*-butyl)-1-(2,2,2-trifluoroethoxy)bicyclo[4.2.0]octane (**P1r-OCH$_2$CF$_3$**).

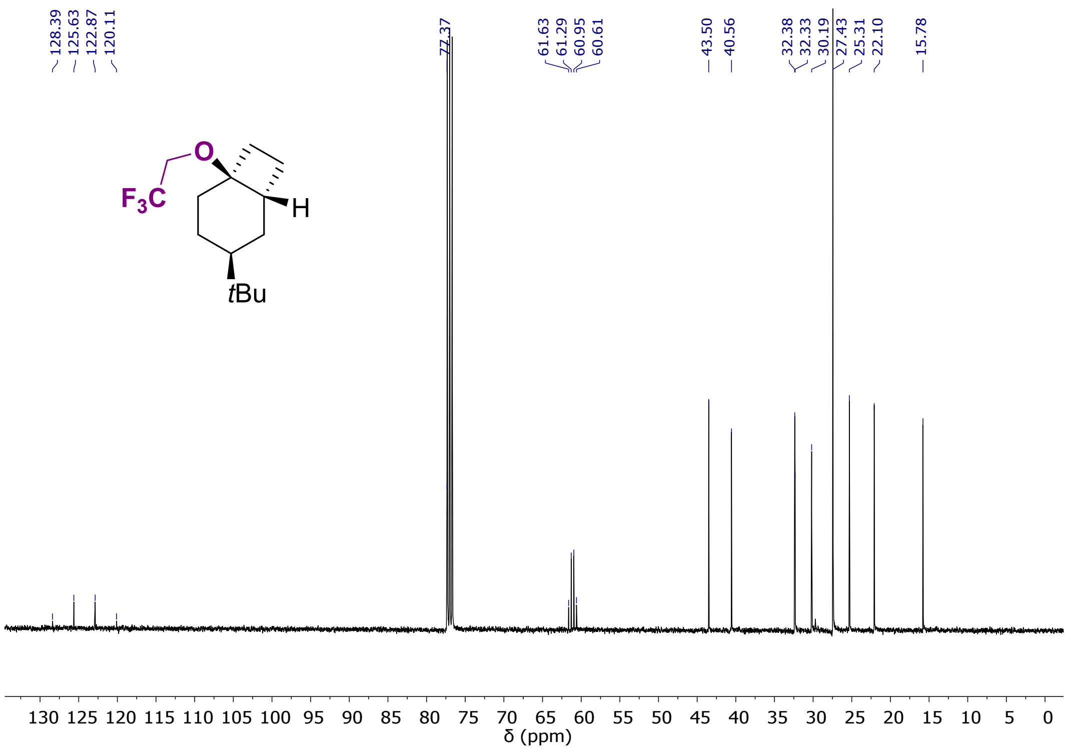

Figure S35. $^{13}C\{^1H\}$-NMR spectrum (400 MHz, $CDCl_3$) of *cis*-4-(*tert*-butyl)-1-(2,2,2-trifluoroethoxy)bicyclo[4.2.0]octane (**P1r-OCH$_2$CF$_3$**).

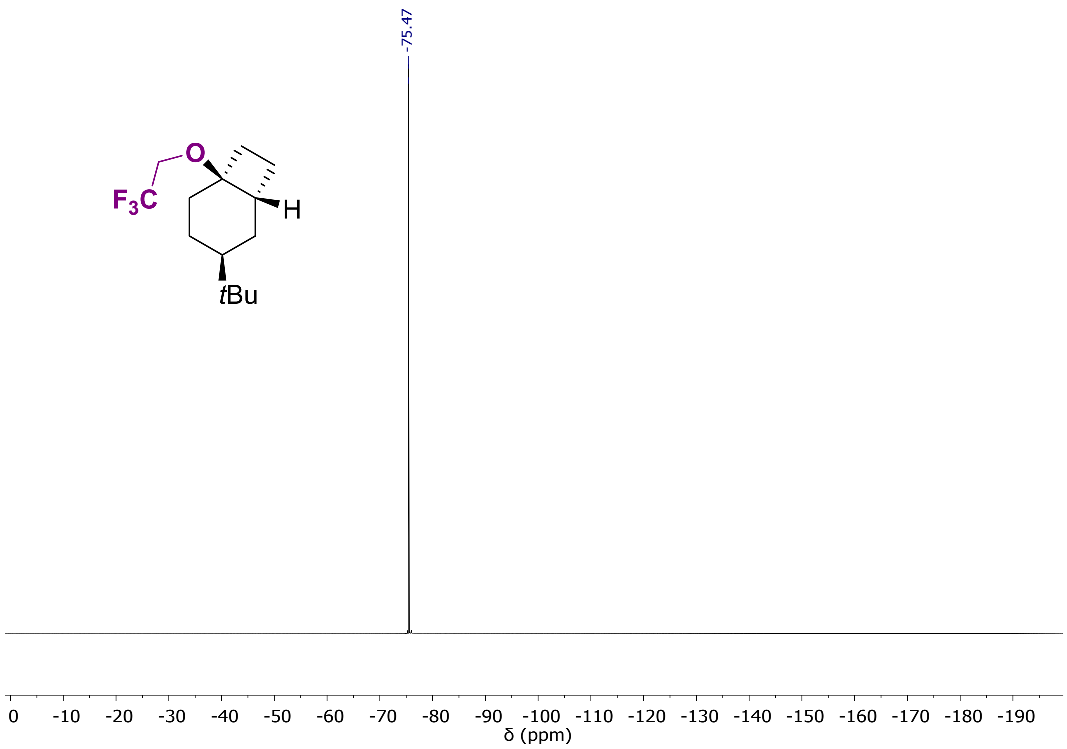

Figure S36. $^{19}F\{^1H\}$-NMR spectrum (400 MHz, $CDCl_3$) of *cis*-4-(*tert*-butyl)-1-(2,2,2-trifluoroethoxy)bicyclo[4.2.0]octane (**P1r-OCH$_2$CF$_3$**).

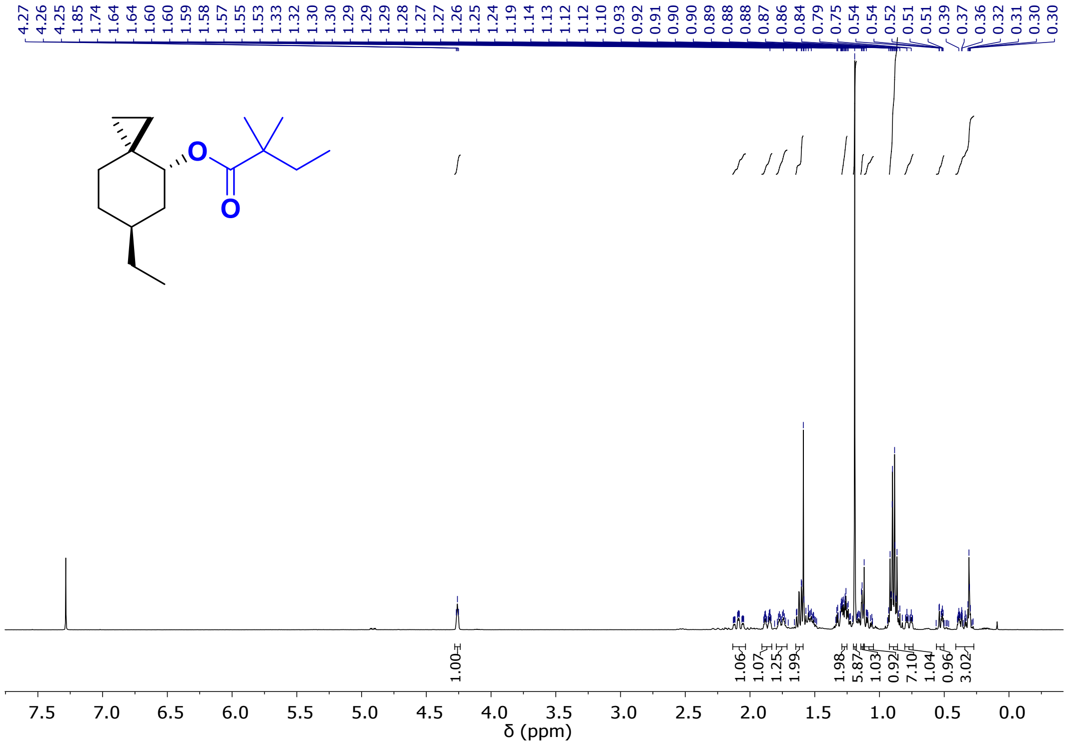

Figure S37. ^{1}H-NMR spectrum (400 MHz, $CDCl_3$) of *trans*-6-ethylspiro[2.5]octan-4-yl 2,2-dimethylbutanoate (**P2u-OX$_7$**).

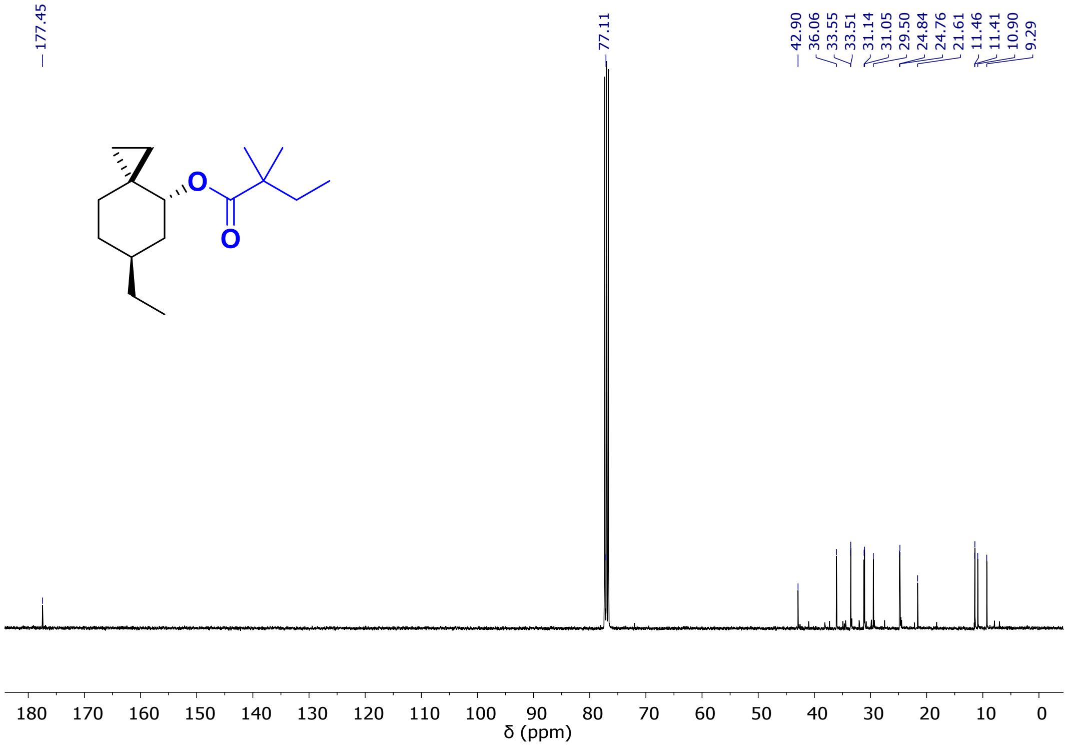

Figure S38. ^{13}C{^{1}H}-NMR spectrum (400 MHz, $CDCl_3$) of *trans*-6-ethylspiro[2.5]octan-4-yl 2,2-dimethylbutanoate (**P2u-OX$_7$**).

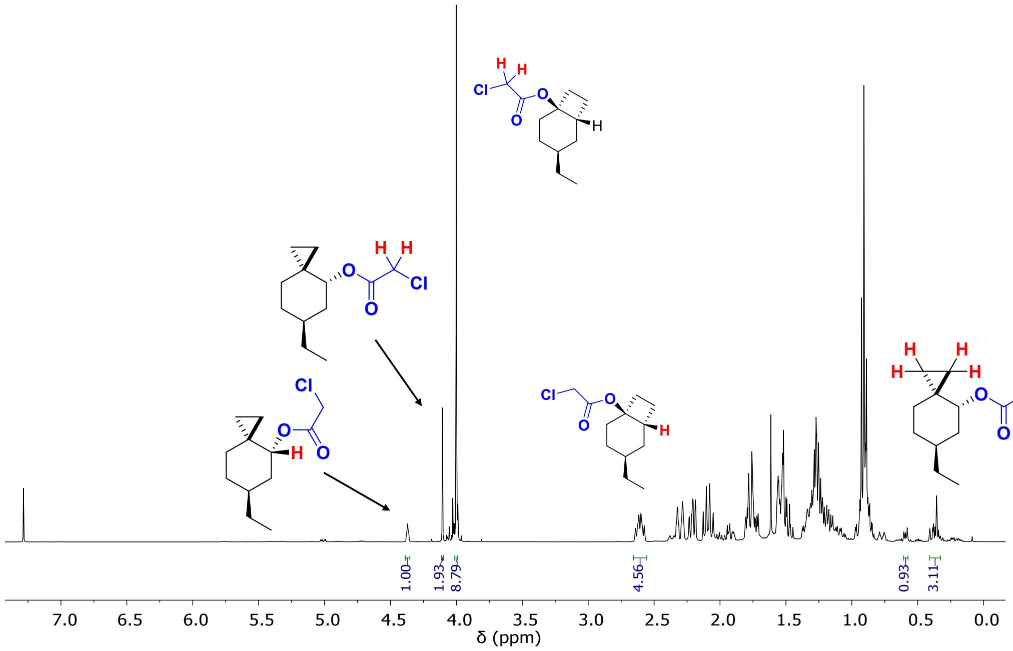

Figure S39. ^{1}H-NMR spectrum (400 MHz, $CDCl_3$) of the *trans*-6-ethylspiro[2.5]octan-4-yl 2-chloroacetate (**P2u-OX$_8$**) and *cis*-4-ethylbicyclo[4.2.0]octan-1-yl 2-chloroacetate (**P2r-OX$_8$**) mixture.

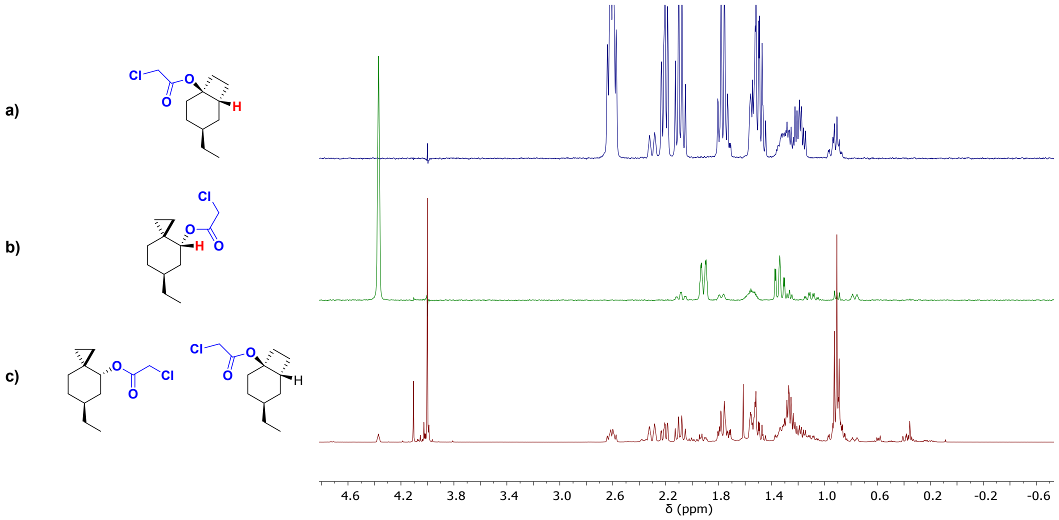

Figure S40. Selective TOCSY NMR spectrum (400 MHz, $CDCl_3$) of the *trans*-6-ethylspiro[2.5]octan-4-yl 2-chloroacetate (**P2u-OX$_8$**) and *cis*-4-ethylbicyclo[4.2.0]octan-1-yl 2-chloroacetate (**P2r-OX$_8$**) mixture. **a)** Irradiation at δ = 4.37. **b)** Irradiation at δ = 2.60. **c)** ^{1}H-NMR spectrum of the mixture.

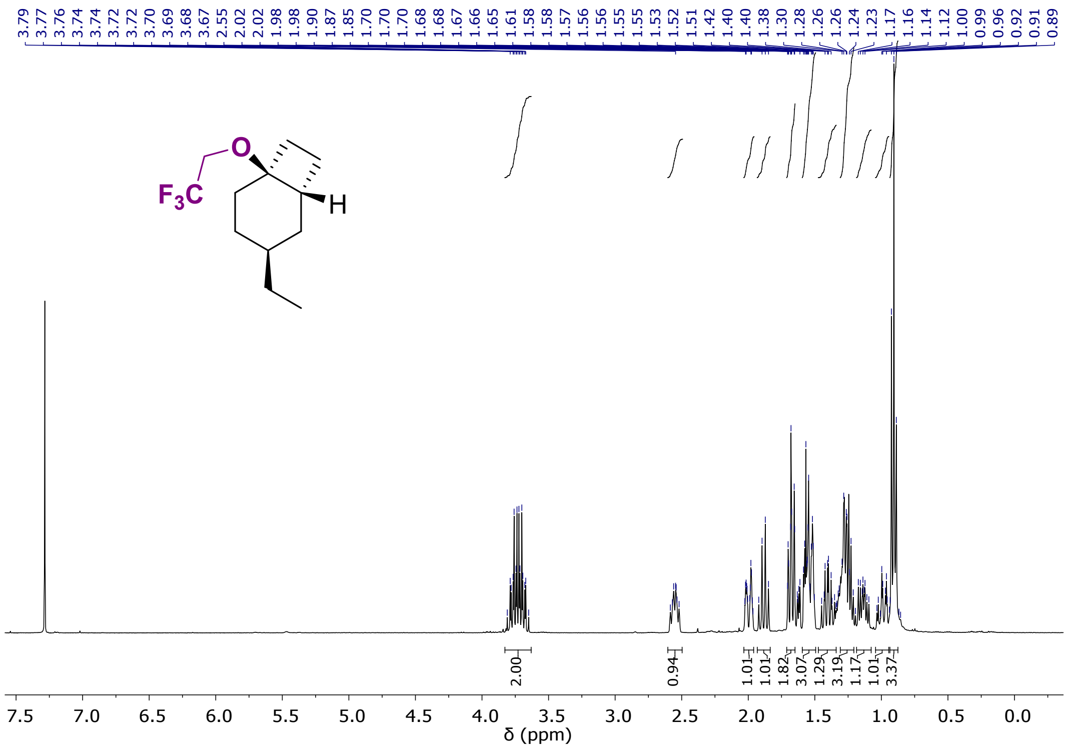

Figure S41. ^{1}H-NMR spectrum (400 MHz, $CDCl_3$) of *cis*-4-ethyl-1-(2,2,2-trifluoroethoxy)bicyclo[4.2.0]octane (**P2r-OCH_2CF_3**).

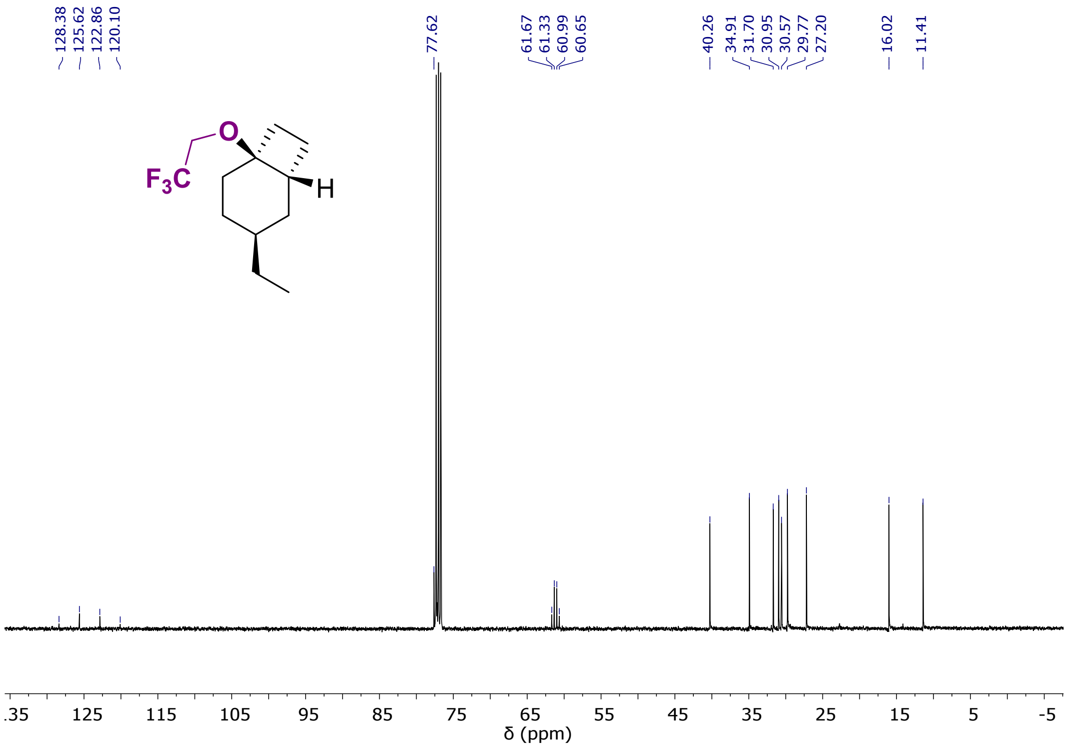

Figure S42. $^{13}C\{^{1}H\}$-NMR spectrum (400 MHz, $CDCl_3$) of *cis*-4-ethyl-1-(2,2,2-trifluoroethoxy)bicyclo[4.2.0]octane (**P2r-OCH_2CF_3**).

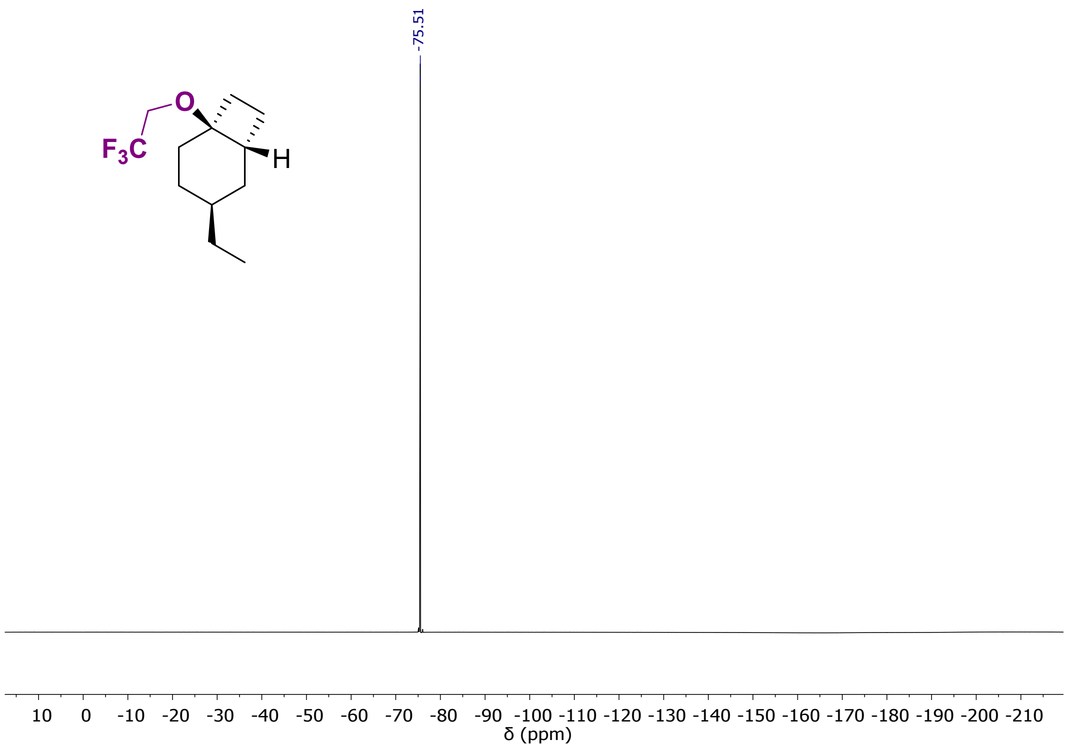

Figure S43. $^{19}F\{^{1}H\}$-NMR spectrum (400 MHz, $CDCl_3$) of *cis*-4-ethyl-1-(2,2,2-trifluoroethoxy)bicyclo[4.2.0]octane (**P2r-OCH$_2$CF$_3$**).

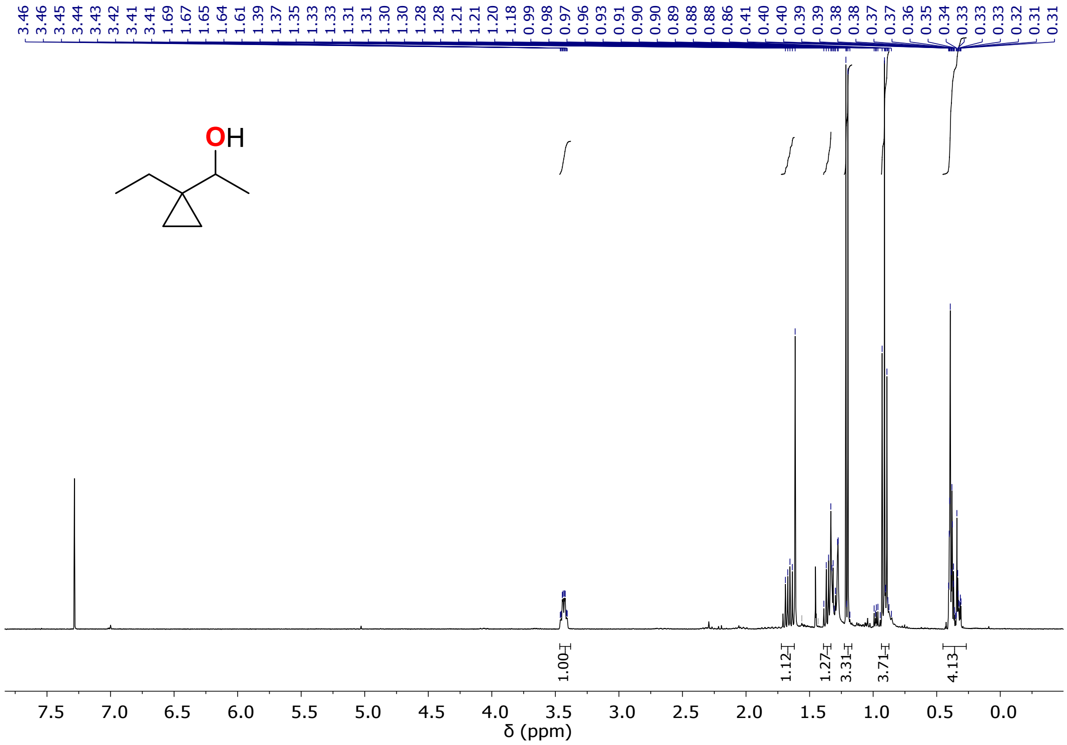

Figure S44. ^{1}H-NMR spectrum (400 MHz, $CDCl_3$) of 1-(1-ethylcyclopropyl)ethan-1-ol **(P3u-OH)**.

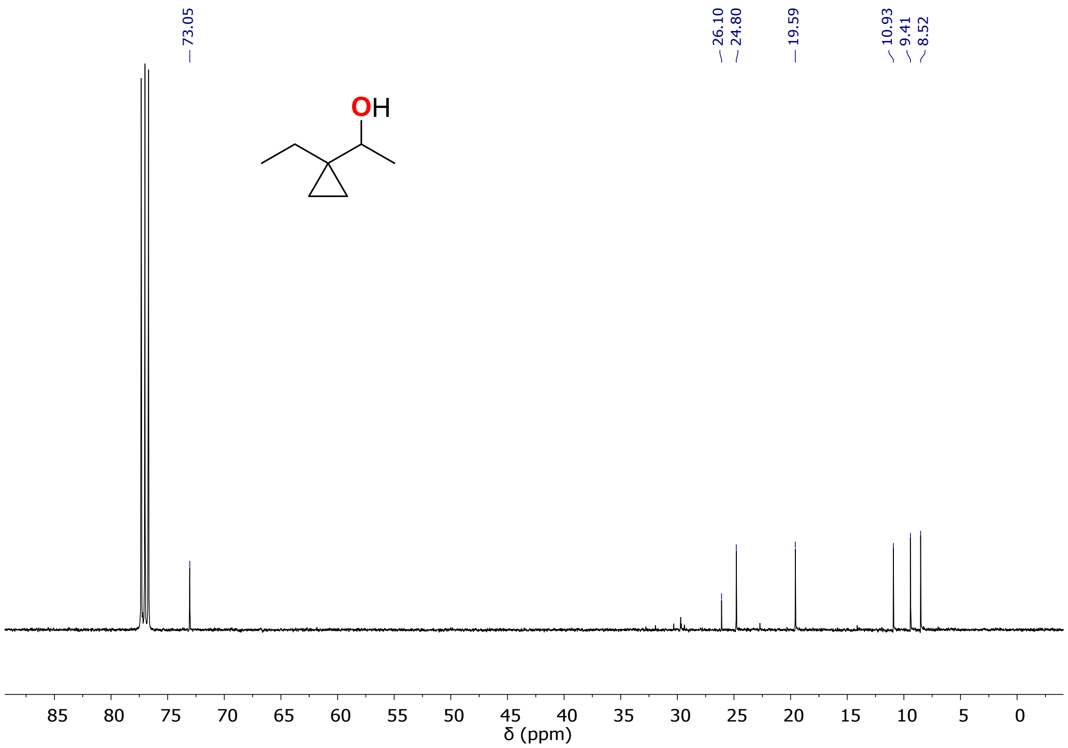

Figure S45. $^{13}C\{^{1}H\}$-NMR spectrum (400 MHz, $CDCl_3$) of 1-(1-ethylcyclopropyl)ethan-1-ol **(P3u-OH)**.

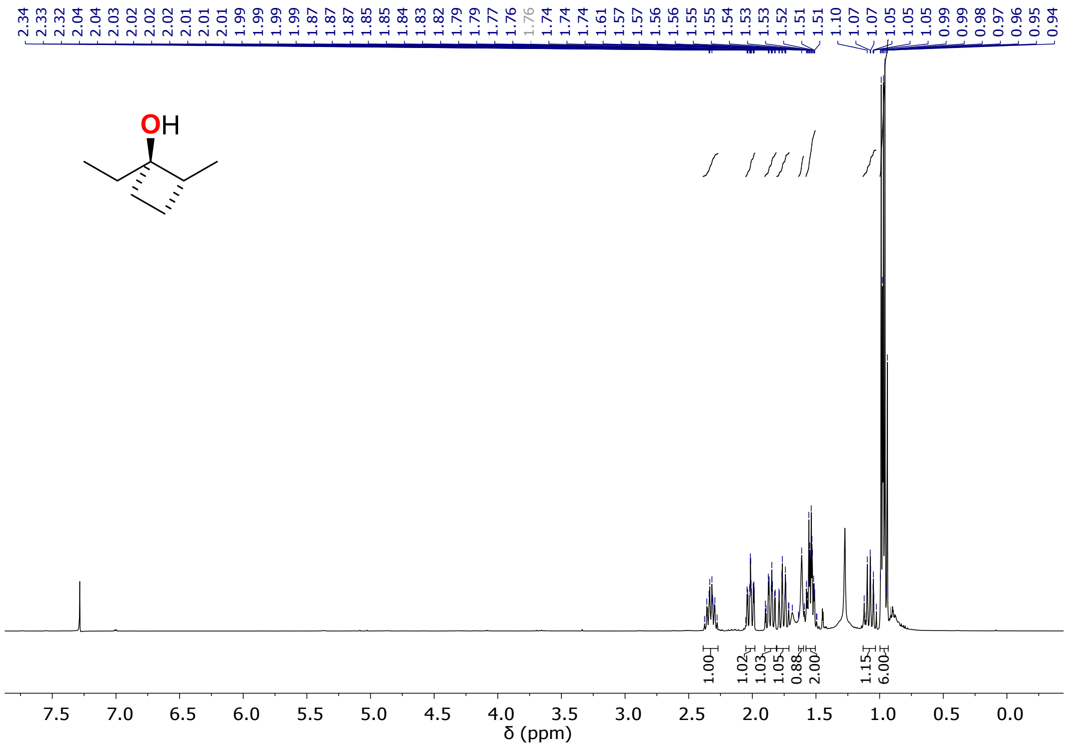

Figure S46. ^{1}H-NMR spectrum (400 MHz, $CDCl_3$) of 1-ethyl-2-methylcyclobutan-1-ol (**P3r-OH**).

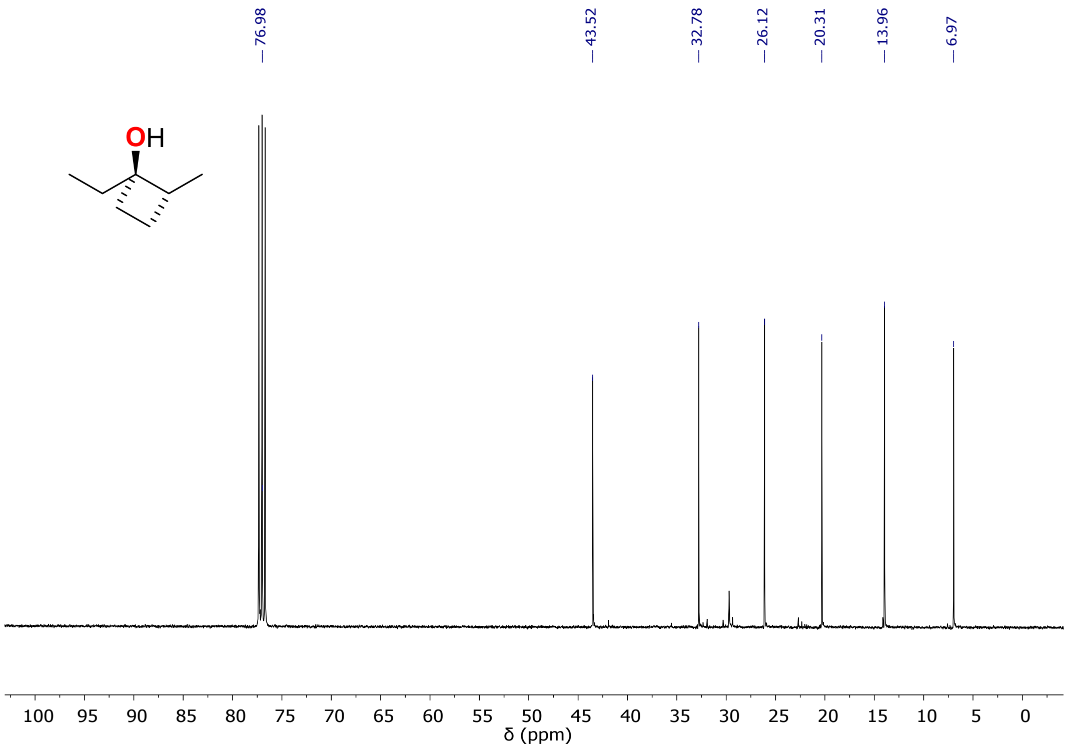

Figure S47. $^{13}C\{^{1}H\}$-NMR spectrum (400 MHz, $CDCl_3$) of 1-ethyl-2-methylcyclobutan-1-ol (**P3r-OH**).

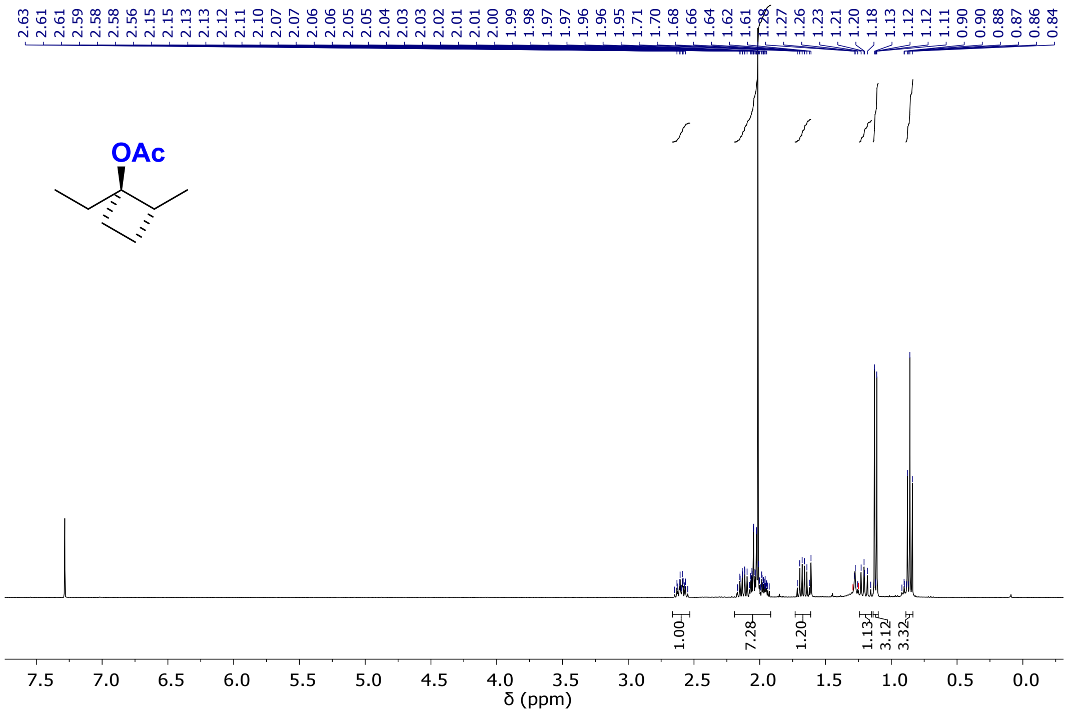

Figure S48. ^{1}H-NMR spectrum (400 MHz, $CDCl_3$) of 1-ethyl-2-methylcyclobutyl acetate (**P3r-OAc**).

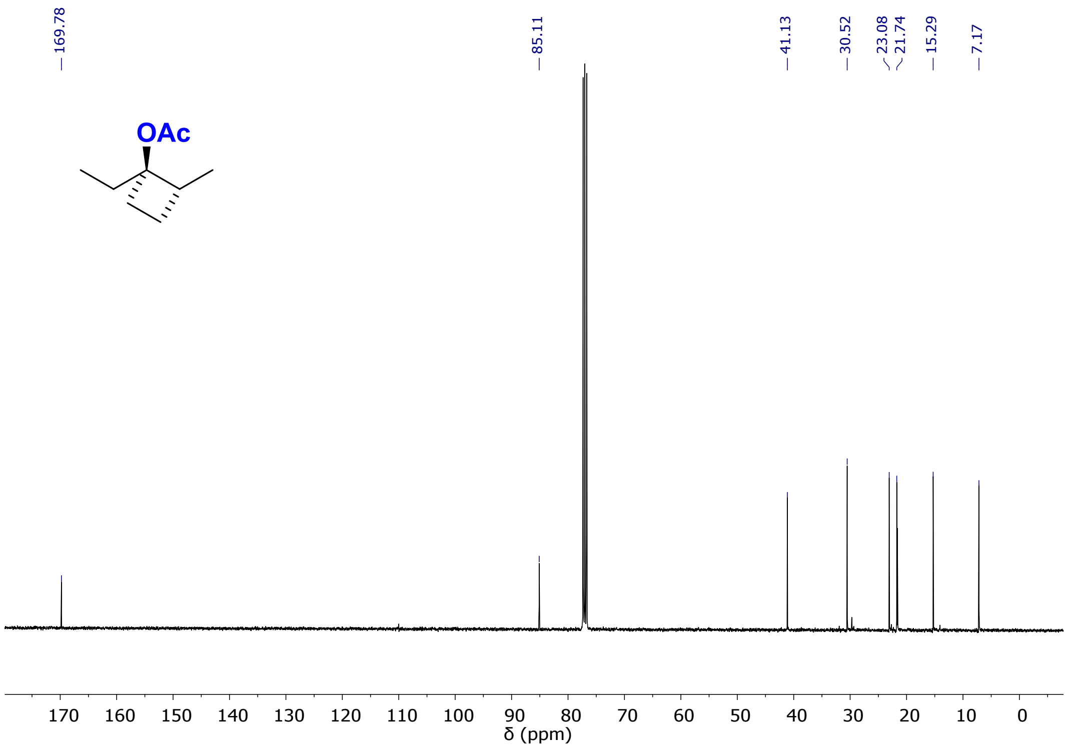

Figure S49. $^{13}C\{^1H\}$-NMR spectrum (400 MHz, $CDCl_3$) of 1-ethyl-2-methylcyclobutyl acetate (**P3r-OAc**).

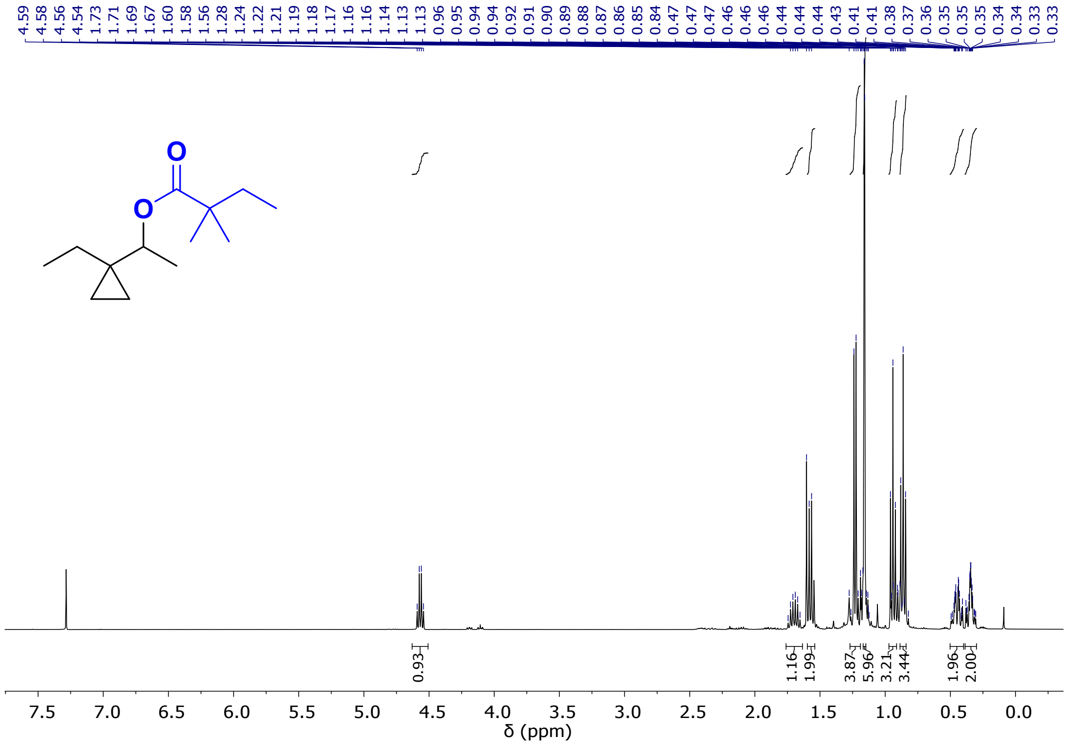

Figure S50. ^1H-NMR spectrum (400 MHz, $CDCl_3$) of 1-(1-ethylcyclopropyl)ethyl 2,2-dimethylbutanoate (**P3u-OX$_7$**).

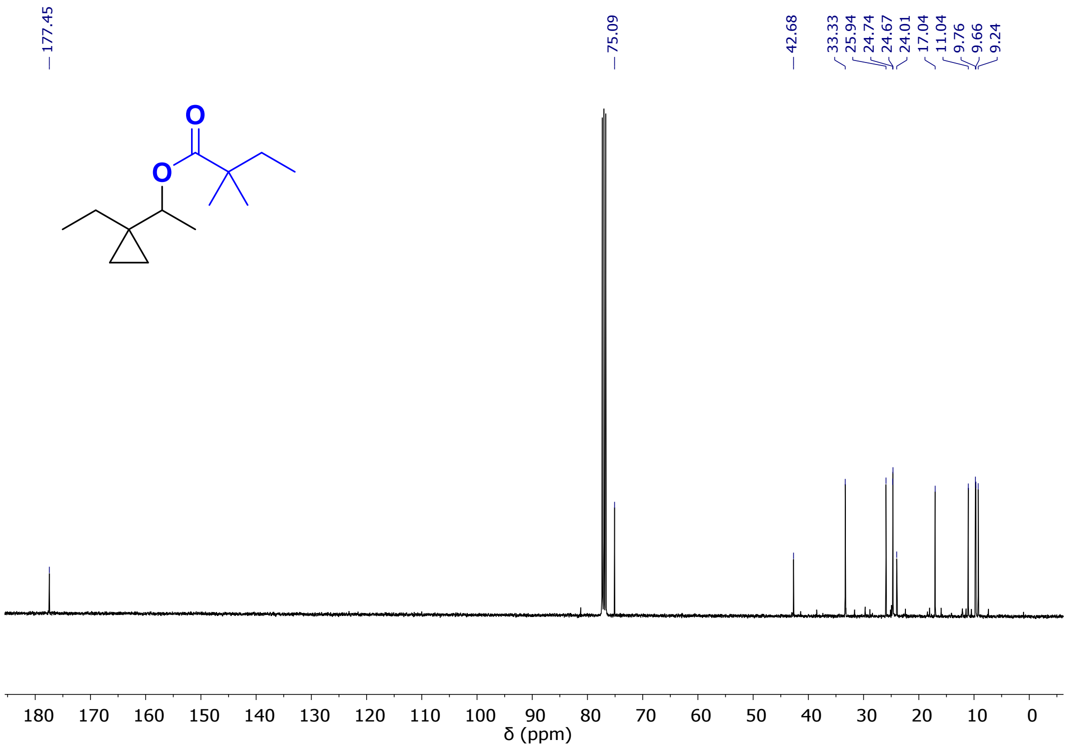

Figure S51. $^{13}C\{^{1}H\}$-NMR spectrum (400 MHz, $CDCl_3$) of 1-(1-ethylcyclopropyl)ethyl 2,2-dimethylbutanoate (**P3u-OX$_7$**).

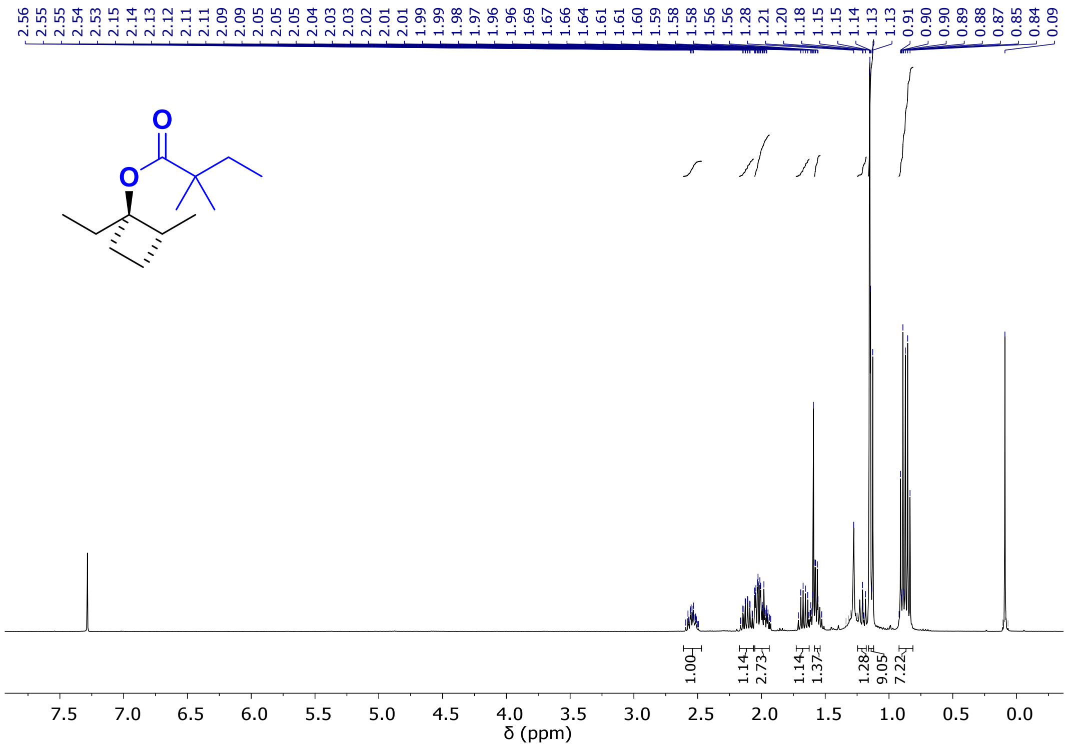

Figure S52. ^{1}H-NMR spectrum (400 MHz, $CDCl_3$) of 1-ethyl-2-methylcyclobutyl 2,2-dimethylbutanoate (**P3r-OX$_7$**).

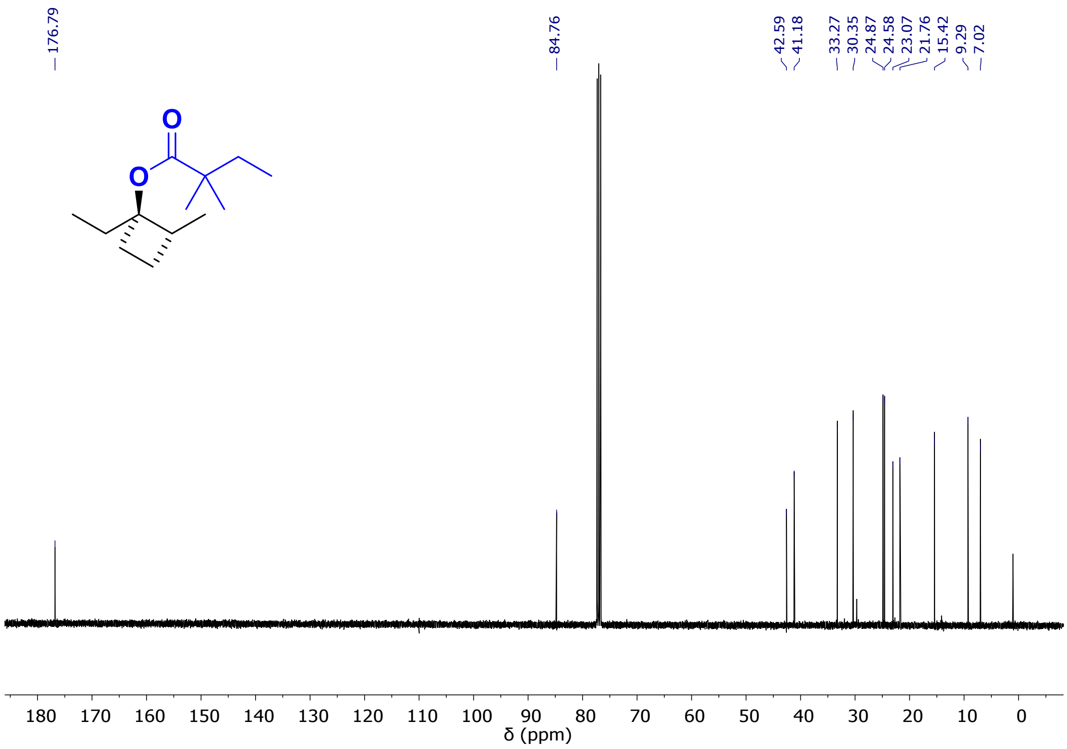

Figure S53. $^{13}C\{^{1}H\}$-NMR spectrum (400 MHz, $CDCl_3$) of 1-ethyl-2-methylcyclobutyl 2,2-dimethylbutanoate (**P3r-OX$_7$**).

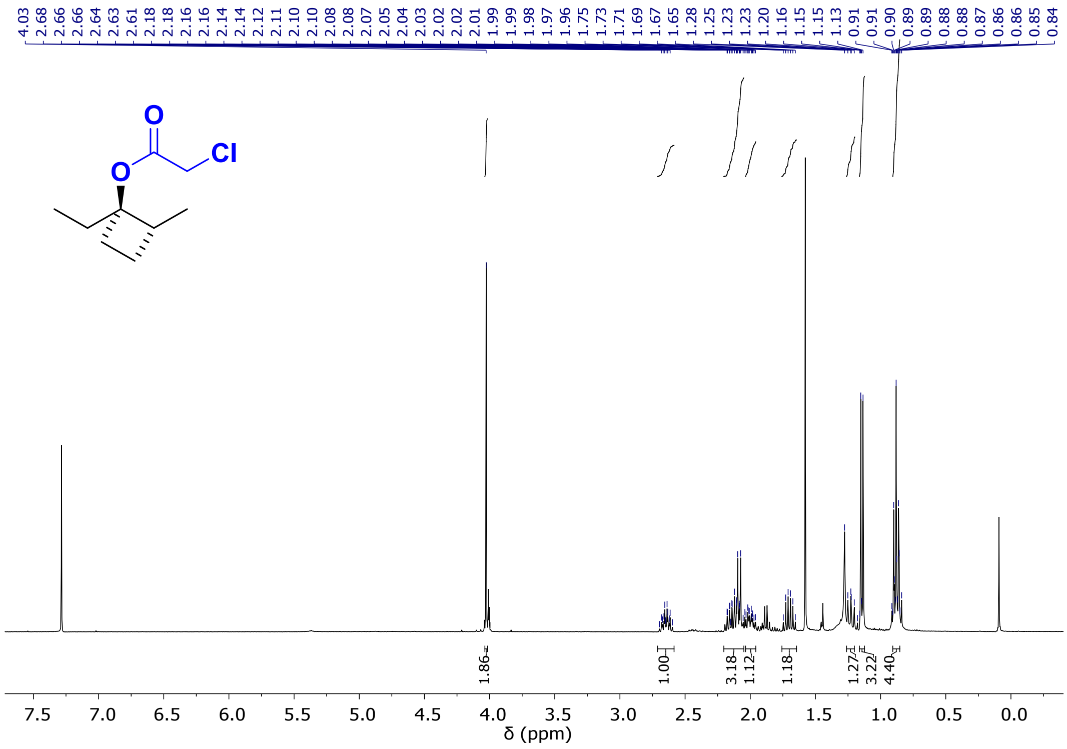

Figure S54. ^{1}H-NMR spectrum (400 MHz, $CDCl_3$) of 1-ethyl-2-methylcyclobutyl 2-chloroacetate (**P3r-OX$_8$**).

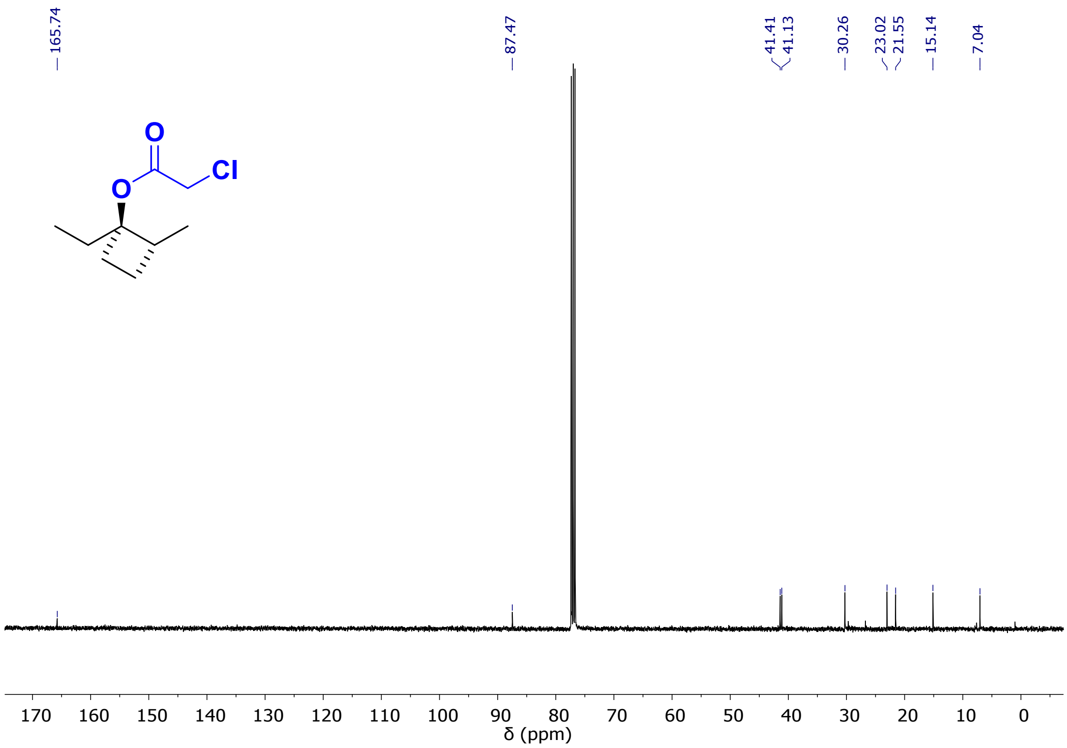

Figure S55. $^{13}C\{^{1}H\}$-NMR spectrum (400 MHz, $CDCl_3$) of 1-ethyl-2-methylcyclobutyl 2-chloroacetate (**P3r-OX$_8$**).

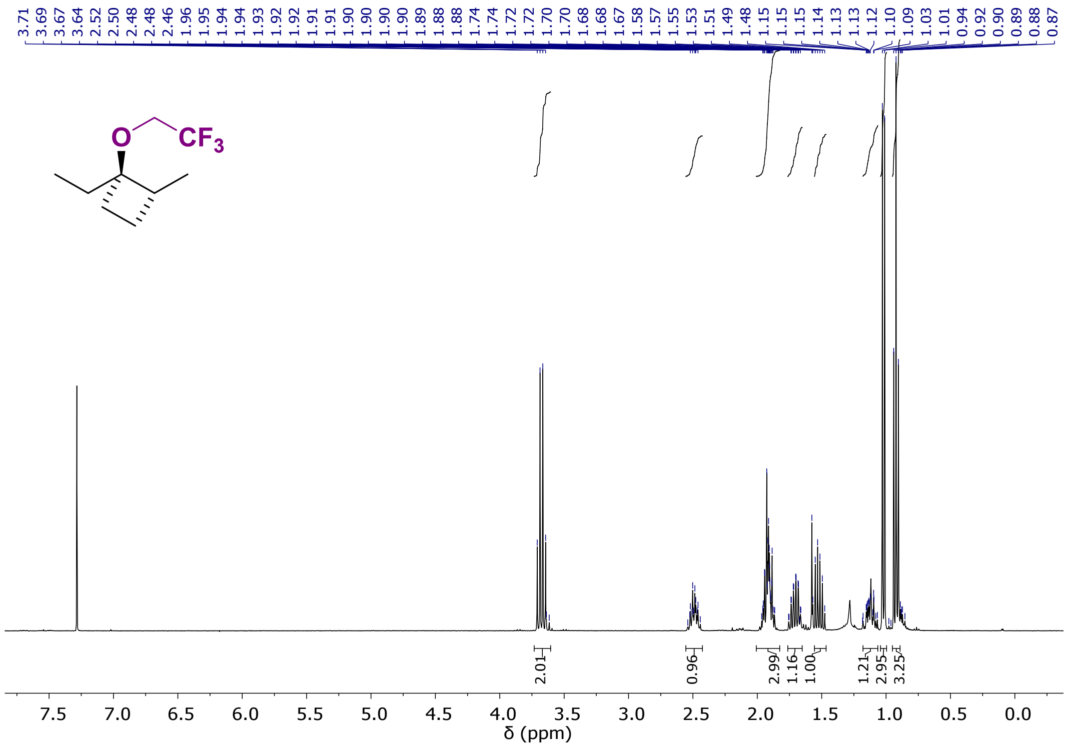

Figure S56. ^{1}H-NMR spectrum (400 MHz, $CDCl_3$) of 1-ethyl-2-methyl-1-(2,2,2-trifluoroethoxy)cyclobutane (**P3r-OCH$_2$CF$_3$**).

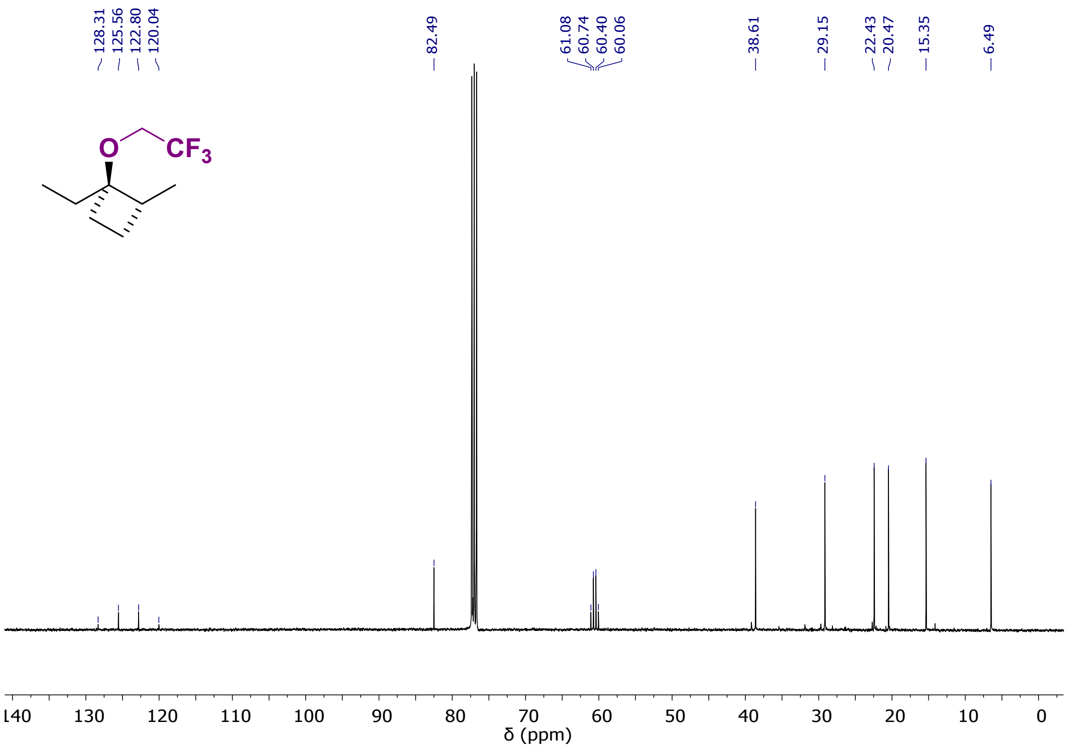

Figure S57. $^{13}C\{^1H\}$-NMR spectrum (400 MHz, $CDCl_3$) of 1-ethyl-2-methyl-1-(2,2,2-trifluoroethoxy)cyclobutane (**P3r-OCH$_2$CF$_3$**).

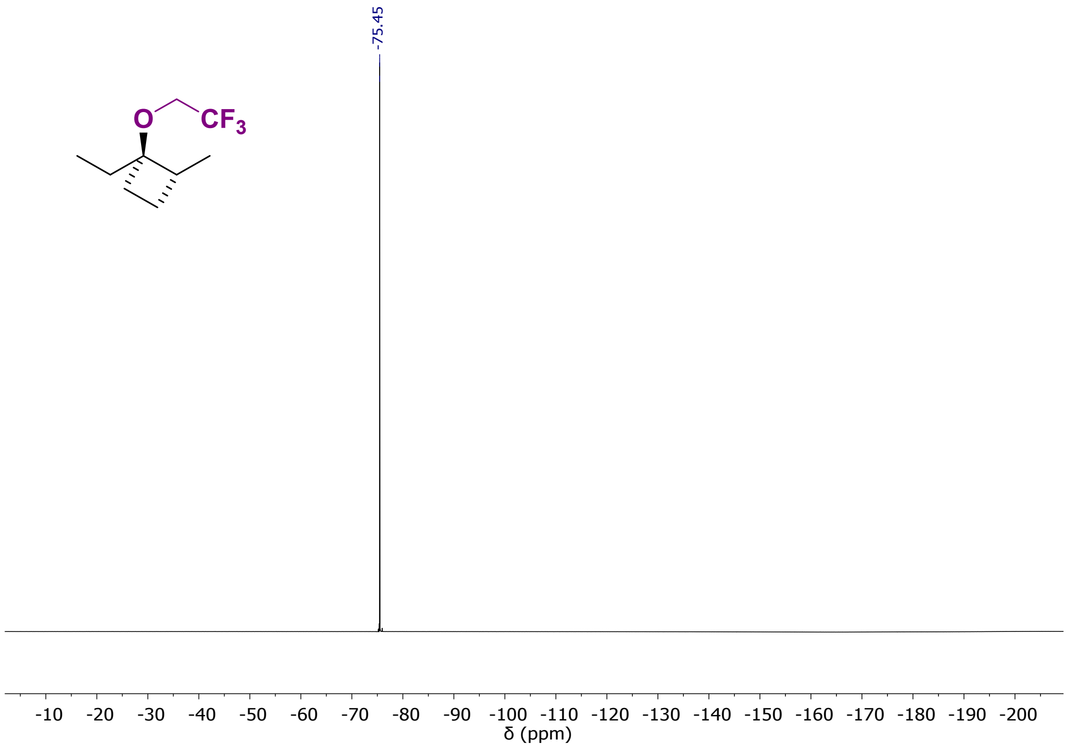

Figure S58. $^{19}F\{^1H\}$-NMR spectrum (400 MHz, $CDCl_3$) of 1-ethyl-2-methyl-1-(2,2,2-trifluoroethoxy)cyclobutane (**P3r-OCH$_2$CF$_3$**).

7. HRMS spectra of the catalysts

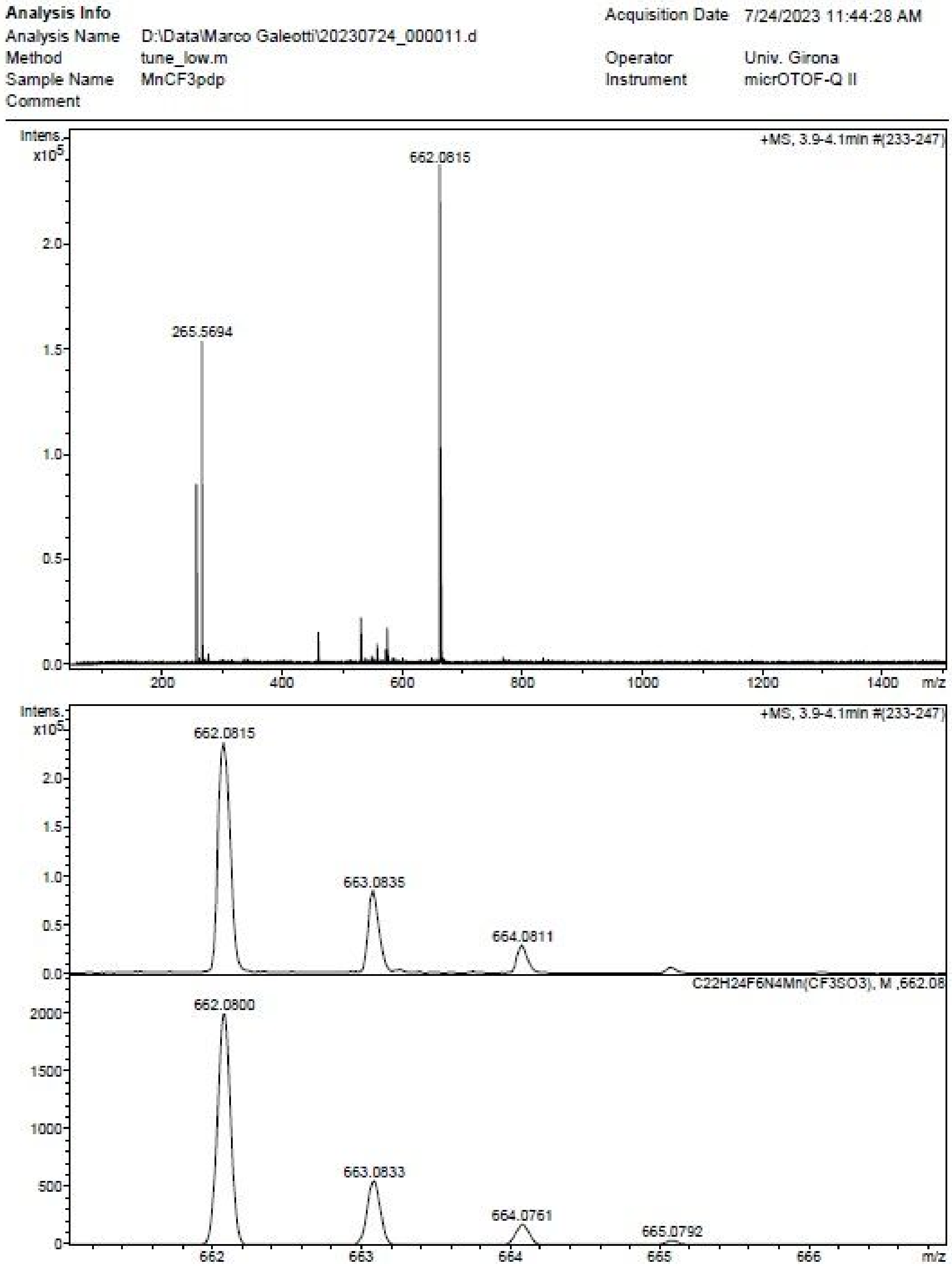

Figure S59. HRMS (ESI-MS) spectrum Mn(^{CF_3}pdp) (**5**).

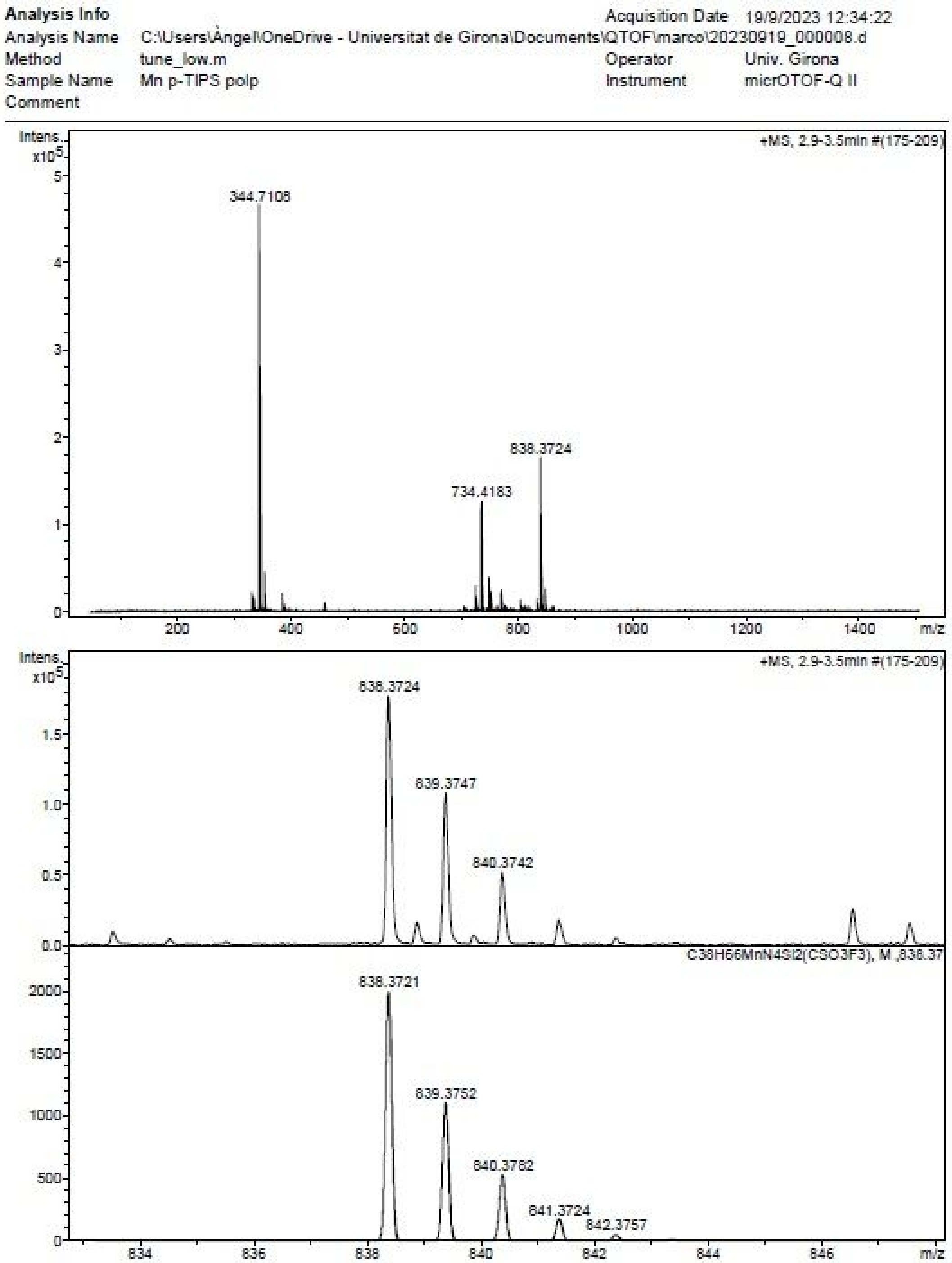

Figure S60. HRMS (ESI-MS) spectrum Mn($^{p\text{-TIPS}}$pdp) (**6**).

8. References

1 Galeotti, M.; Vicens, L.; Salamone, M.; Costas, M.; Bietti, M. *J. Am. Chem. Soc.* **2022**, *144*, 7391-7401.

2 Luo, W.; Lin, L.; Zhang, Y.; Liu, X.; Feng, X. *Org. Lett.* **2017**, *19*, 3374-3377.

3 Wolfgang R. R.; Christian, U. *Liebigs Annalen* **1995**, *7*, 1361-1366.

4 Bailey, W. F.; Gagnier, R. P.; Patricia, J. J. *J. Org. Chem.* **1984**, *49*, 2098-2107.

5. Milan, M.; Bietti, M.; Costas, M. *ACS Cent. Sci.* **2017**, *3*, 196-204.

6 Ottenbacher, R. V.; Bryliakov, K. P.; Talsi, E. P. *Adv. Synth. Catal.* **2011**, *353*, 885-889.

7 Cussó, O.; Garcia-Bosch, I.; Font, D.; Ribas, X.; Lloret-Fillol, J.; Costas, M. *Org. Lett.* **2013**, *15*, 6158-6161.

8 Eisenbraun, E. J. *Org. Synth.* **1973**, *5*, 310-312.
